# Fungal spores as a source of sodium salt particles in the Amazon basin

**DOI:** 10.1038/s41467-018-07066-4

**Published:** 2018-11-19

**Authors:** Swarup China, Susannah M. Burrows, Bingbing Wang, Tristan H. Harder, Johannes Weis, Meryem Tanarhte, Luciana V. Rizzo, Joel Brito, Glauber G. Cirino, Po-Lun Ma, John Cliff, Paulo Artaxo, Mary K. Gilles, Alexander Laskin

**Affiliations:** 1 William R. Wiley Environmental Molecular Sciences Laboratory, Pacific Northwest National Laboratory, Richland, WA 99354 USA; 2 Atmospheric Sciences and Global Change Division, Pacific Northwest National Laboratory, Richland, WA 99354 USA; 3 State Key Laboratory of Marine Environmental Science, College of Ocean and Earth Sciences, Xiamen University, Xiamen, 361102 China; 4 Chemical Sciences Division, Lawrence Berkeley National Laboratory, Berkeley, CA 94720 USA; 5 Department of Chemistry, University of California, Berkeley, Berkeley, CA 94720 USA; 6 Max Planck Institute for Chemistry, Mainz, 55128 Germany; 7 Department of Environmental Sciences, Universidade Federal de Sao Paulo, Diadema, 09961 SP Brazil; 8 Institute of Physics, University of São Paulo, Rua do Matão 1371, CEP 05508-090 São Paulo, SP Brazil; 9 Geosciences Institute, Federal University of Para, Belem, 66075-110 Brazil; 10 Department of Chemistry, Purdue University, West Lafayette, IN 47907 USA; 11 Present Address: Physikalisches Institut, Universität Würzburg, Am Hubland, 97074 Würzburg, Germany; 12 Present Address: IMT Lille Douai, SAGE, Univ. Lille, 59000 Lille, France

## Abstract

In the Amazon basin, particles containing mixed sodium salts are routinely observed and are attributed to marine aerosols transported from the Atlantic Ocean. Using chemical imaging analysis, we show that, during the wet season, fungal spores emitted by the forest biosphere contribute at least 30% (by number) to sodium salt particles in the central Amazon basin. Hydration experiments indicate that sodium content in fungal spores governs their growth factors. Modeling results suggest that fungal spores account for ~69% (31–95%) of the total sodium mass during the wet season and that their fractional contribution increases during nighttime. Contrary to common assumptions that sodium-containing aerosols originate primarily from marine sources, our results suggest that locally-emitted fungal spores contribute substantially to the number and mass of coarse particles containing sodium. Hence, their role in cloud formation and contribution to salt cycles and the terrestrial ecosystem in the Amazon basin warrant further consideration.

## Abstract (web summary)

Salt particles in the Amazon basin are typically attributed to marine aerosols transported from the Atlantic Ocean. Here the authors show the potential importance of fungal spores as a source of sodium-salt particles in the Amazon rainforest.

## Introduction

Atmospheric conditions in the Amazon rainforest are relatively pristine and in terms of the low particle count and cloud characteristics resemble conditions in remote marine regions^1–3^. Specifically, the size distribution and concentration of coarse mode particles in Amazonia are similar to concentrations of coarse mode particles in marine air above the ocean under calm wind conditions of 1–10 m s^−1^ (Supplementary Fig. 1). During the wet season in the Amazon basin, marine aerosol from the Atlantic Ocean is considered the dominant source of coarse mode particles^1,4^. Previous studies assumed the chemical composition of coarse mode aerosols was strongly impacted by long-range transport of soil dust and marine aerosol advected into the central Amazon by large-scale tropospheric circulation^5^. However, local sources of sodium salt-containing coarse particles such as primary biological aerosol particles (PBAPs) and their potential impact have not been considered. This study shows that locally emitted fungal spores of coarse super-micrometer size contribute considerably to sodium salt particles in the Amazon basin during the wet season. While previous studies reported non-marine sources of sodium salts in fine particles, sodium-containing coarse particles have been attributed to sea salt. Specifically, Ooki et al.^6^ observed anthropogenic sodium in fine mode (<1.1 µm) aerosol particles in urban air. Ooki et al^6^ suggested using potassium as a tracer for anthropogenic sodium in submicron size (0.43 µm) particles. Mamane^7^ detected the presence of sodium and potassium in <1 µm particles from waste incinerator emissions. A study in the metropolitan area of São Paulo found K/Na ratios of 1.4 and 0.9 for coarse (>2.5 µm) and fine (<2.5 µm) particles, respectively, which were attributed to waste incineration and vehicles^8^.

PBAPs, i.e., particles emitted directly from the biosphere, include fungal spores, pollen, bacteria, algae, protozoa, and fragments of plants and living or dead organisms. Fungal species actively discharge their spores via liquid jets into the air^9,10^, a process occurring preferentially under humid atmospheric conditions^10^. Total global emissions of fungal spores are highly uncertain^11–13^; estimates vary from 8 Tg yr^−1^ to 186 Tg yr^−1^, to as large as 1000 Tg yr^−1^. Fungal spores and fragments are the most abundant classes of biological particles in the Amazon basin. They contribute up to 25% during daytime (and 45% during nighttime) to the total number of coarse mode particles (1 to 10 µm diameter)^1^.

Biological particles, as well as fluorescent particles (inferred to be biological), have been detected in the free troposphere^14,15^. In Amazonia, concentrations of fluorescent biological particles during the wet season of ~7.3 × 10^−4^ m^−3^ are reported, with the highest concentrations occurring during nighttime^16^. Chemical compositions of biological particles are highly variable and, due to the inherent challenges involved in analytically distinguishing between biological and other carbonaceous particles, their exact origins remain insufficiently characterized^1,13^.

Once in the troposphere, biological particles can act as cloud condensation nuclei and ice nuclei, thus impacting warm and cold cloud formation and evolution^13,17^. During their airborne lifetime, Amazonian particles typically experience several cycles of cloud processing^1^ which alters their physio-chemical properties and further thwarts their identification. In particular, biological spores rupture at high humidity due to osmotic pressure and subsequently release submicron-sized fragments^18,19^. These fragments contain inorganic salts with the same elements (e.g., Na, Mg, K, Cl) as sea salt. While fragmentation reduces the fraction of original supermicron-sized biological particles, it increases their contribution to the number concentration of submicron particles. Hence, fragmentation due to atmospheric processing further complicates estimation of their number and size distributions in the accumulation mode^18^.

This study highlights the role of fungal spores emitted by the forest biosphere in the Amazon basin and their potential impacts on the terrestrial ecosystem. Samples of airborne biological particles were collected during the beginning of the wet season (January and February, 2015) at the ZF2 tower, a pristine rainforest site in Central Amazonia located 40 km North of Manaus^4^. We applied multimodal chemical imaging techniques, such as scanning electron microscopy (SEM) coupled with energy dispersive X-ray (EDX) microanalysis, scanning transmission X-ray microscopy with near-edge X-ray absorption fine structure analysis (STXM/NEXAFS), and nano secondary ion mass spectrometry (NanoSIMS) to analyze size and composition of the biological particles. Results show that locally emitted fungal spores (primary biogenic aerosol particles) in the forest biosphere contribute considerably to total sodium salt particles observed in the central Amazon basin during the wet season. In situ water vapor experiments using environmental microreactors complemented with micro-spectroscopy analysis show that sodium-containing fungal spores have higher hygroscopic growth than sodium-free spores. Finally, model simulations examining the potential contributions of fungal spores in the Amazon basin atmosphere suggest that they substantially contribute to the total sodium budget during the wet season.

## Results

### Chemical imaging and micro-spectroscopy of fungal spores

**Fig. 1:**
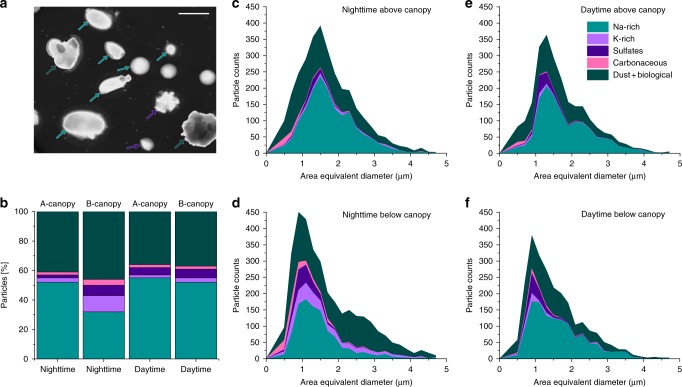
Size distribution and particle-type classes. **a** Representative SEM image (forward scattered transmitted electron imaging mode) of particles collected at the ZF2 site in Amazonia. Dark green arrows indicate Na-rich particles, blue arrows indicate mixed sulfate and Na-containing particles with minor contributions of Na, and light green color arrows indicate other types of particles, mostly internally mixed dust and biological particles. Scale bar is 5 µm. **b** Nightime and daytime number fraction of different particle classes for particles collected above canopy (A-canopy) and below canopy (B-canopy). **c** Size distribution of different particle classes during the nighttime above the canopy. **d** Size distribution of different particle classes during the nighttime below the canopy. **e** Size distribution of different particle classes during the daytime above the canopy. **f** Size distribution of different particle classes during the daytime below the canopy

**Fig. 2:**
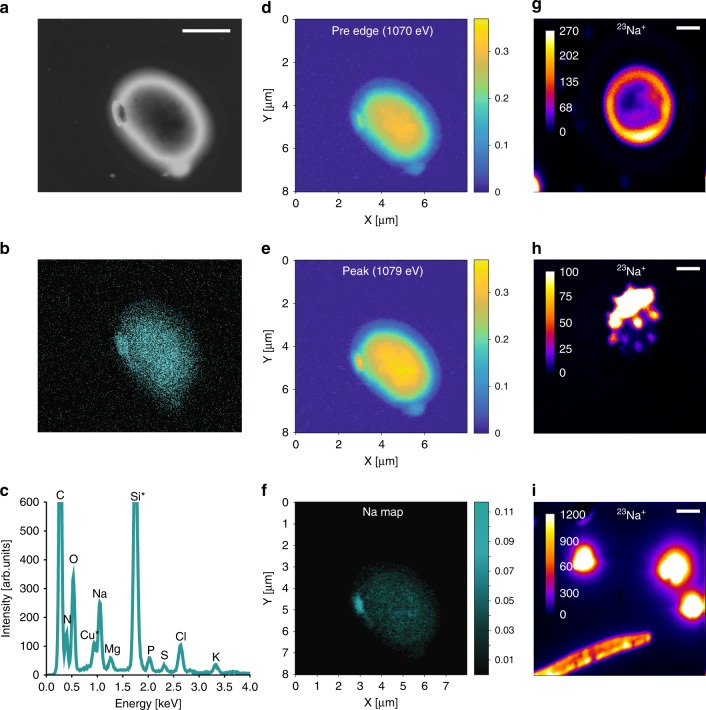
Chemical imaging of Na-containing biological particles. **a** SEM image **b** elemental Na map and **c** EDX spectra of the biological particle. The Cu and Si in the EDX spectra are background peaks originating from the substrate and various instrument parts inside the SEM chamber. STXM images of the same biological particle: **d** pre edge (1070 eV), **e** Na peak (1079 eV) and **f** Na optical density map. Color bars indicate optical density. **g**–**i** Representative NanoSIMS images of ^23^Na^+^ of selected biological particles showing various distributions of sodium within particles contours. Scale bars are 2 µm

SEM images of representative coarse mode particles collected in the Amazon basin are illustrated in Fig. 1a. X-ray microanalysis using computer-controlled (CC) SEM/EDX reveals that nearly half (~48%) of the coarse mode particles (1.0–3.2 µm aerodynamic diameter) are Na rich (Fig. 1b). Dust (containing Al, Si, Fe) or internally mixed biological-dust particles (C, N, P, Na, K, Al, Si, and Fe) are the second most abundant type of particles. Coarse mode sulfate and purely carbonaceous particles contribute 5 and 3%, respectively. Overall, no considerable differences in the size distributions of different particle classes were observed during night or day or within, above, or below the canopy (Fig. 1c–f). X-ray microanalysis indicates that the spores are comprised primarily of C, N, and O (carbonaceous in nature) with substantial amounts of other elements, e.g., Na, Mg, P, S, Cl, and K (Fig. 2a–c), consistent with previous reports^18,20,21^.

The fraction of fungal spores was quantified based on their unique characteristic morphologies (spherical, rod-like, or spheroidal in shape), size (1–6 µm), and chemical composition (mostly carbonaceous and containing phosphorous) by electron microscopy imaging and X-ray microanalysis of over 3500 individual particles. Details of the fungal spore identification method are provided elsewhere^18^. The particle-type classification scheme is summarized in Supplementary Fig. 2. During sampling, the number fraction of biological particles was higher below the canopy (60% ± 2%) than above the canopy (38% ± 2%) and higher during night (52% ± 2%) than day (37% ± 2%). The coarse mode mass fractions of phosphorous and potassium, primarily derived from biological particles^10,22^, were larger below than above the canopy and larger during night than day^23^. The higher fraction of biological particles below the canopy is consistent with a local source, and with observations from other tropical rainforests such as Borneo, Malaysia^24^. The increased nighttime number fraction of spores could result from enhanced nighttime active wet discharge of ascospores and basidiospores associated with elevated humidity^10^. Our observation of higher fractions of biological particles during nighttime below the canopy is consistent with previous studies in the Amazon rainforest^16,23,25^.

Chemical imaging of the biological particles using X-ray absorption micro-spectroscopy confirms the presence of sodium. Figure 2d–f shows representative STXM images of a biological particle at the Na pre edge (1070 eV) and peak (1079 eV) as well as an optical density map. The sodium atomic weight fraction is size dependent; smaller particles contain a higher fraction of sodium than larger particles (Supplementary Fig. 3). The frequent occurrence of substantial sodium and chlorine masses in biological spores is remarkable. Since biological spores have never been considered as sources of sodium and chlorine, common analytical methods could mistakenly assign them as transported sea salt.

NanoSIMS analysis of fungal spores confirms the presence of sodium-containing particles (Fig. 2g–i) and provides spatial distributions of sodium and other elements within individual particles^26^. For example, the particle in Fig. 2g shows a stronger sodium signal around the particle boundary, and that in Fig. 2h displays complex morphology and heterogeneity in its sodium spatial distribution, while the distribution of sodium ions in the elongated fungal spore in Fig. 2i appears homogeneous. These images suggest that sodium distributions within individual fungal spores are diverse, presumably depend upon spore type, and may be influenced by the particle’s origin and aging history.

X-ray microanalysis indicates that the average percentage of total spores containing sodium (minimum detection of 3 wt%) is higher above the canopy (daytime: 60% ± 6%; nighttime: 39% ± 3%) than below the canopy (daytime: 46% ± 4%; nighttime: 22% ± 2%). These differences may partially be influenced by changes in physio-chemical properties of spores that transform during atmospheric processing. For example, when exposed to high humidity conditions, spores rupture and release submicrometer-to-micrometer size fragments. A major fraction (~40–60%) of these fragments contains Na and Cl, and appears morphologically similar to dry sea salt particles (Supplementary Fig. 4). Subsequently, when exposed to high humidity, these hygroscopic fragments grow and become supermicron^18^.

### Hygroscopicity of fungal spores

We investigated the hygroscopicity of spores using microreactors in environmental SEM^18^ and in situ STXM^27^. At 94% relative humidity (RH), sodium-containing fungal spores had area growth factors of 2.4 versus 1.1 for sodium-free spores (Supplementary Figs. 5, 6). Similarly, mass growth factors of 3.6 and 1.5 were determined for sodium-containing and sodium-free fungal spores, respectively, at 96% RH. Remarkably, under high humidity conditions (RH~94%) the growth factors of sodium-containing biological particles and NaCl particles are strikingly similar. Using standard analytical methods or inspecting their morphology^18^, these processed particles would not readily be recognized as spores. Due to the wide range of sources relevant to biological particle emissions, which is further complicated by fragmentation during atmospheric processing, detecting and apportioning aged spores is a daunting task. Hence, microscopy methods underestimate the contribution of biological particles to the total atmospheric aerosol. As a result, the fractions of sodium-containing spores reported here represent lower limits for sodium-bearing fungal spores.

### Presence of sodium in fungal spores

Previous studies highlighted the presence of biogenic potassium-rich particles in the Amazonia in the accumulation mode (0.1–1 µm diameter)^22^. However, biological particles have never been indicated as a significant source of sodium-containing particles in the Amazonia. This is despite the fact that sodium in biological particles has been reported previously.^20,28^ For example, laser-induced breakdown spectroscopy measurements showed the presence of sodium in fungal spores^28^ and an X-ray microanalysis study observed sodium in particles of biogenic origin^29^. The presence of sodium is common in halophilic fungi which cannot grow without NaCl^30^. Active discharge, uptake, and efflux processes are likely responsible for the sodium content in the spores and sodium content may vary with different classes and genera of fungi. During active discharge, fungi forcibly eject spores into the atmosphere, together with osmotic fluid containing hexoses, mannitol, phosphate, sodium, and potassium^22,31^. Fungi require K^+^ for electrical and osmotic equilibria of the cells and in several fungal spores the role of K^+^ is well understood^32^. Previous studies suggested that K^+^ can be partially replaced by Na^+^ and Na^+^ can enhance the growth of fungi^33^ and plants^34^ under K^+^-deficient conditions. The growth of fungi and uptake of Na^+^ varies with their physiological conditions and uptake rates depend on the species and their genes^35^. For example, specific genes (e.g., acu1 and acu2) are responsible for high-affinity Na^+^ uptake of Ustilago maydis. When fungi contain excess Na^+^, they activate Na^+^-efflux ATPase (adenosine triphosphatase) which acts as a key enzyme for the biological evolution of fungi^36^. We suggest that uptake and efflux of Na^+^ varies with different classes and genera of the fungal community. For example, previous studies in Amazonia shows several genera (e.g., Agaricus; Amanita; Aspergillus; Boletus; Cladonia; Mortierella; Puccinia; Lepsita; and Rhizopus) within one class of fungi (Lecanonomycetes)^37,38^. Furthermore, the transpiration of plants^39^ and nutrient uptake^40^ can also influence the sodium content of spores. Further studies are needed to better comprehend the sodium content in fungal spores by linking chemical composition, molecular biology, and diversity of fungal spores.

In contrast to previous reports^1,5^, our analyses suggest that locally emitted biological particles contribute substantially to the total concentration of sodium salt particles in the Amazon basin during the wet season. Backward trajectories calculated with NOAA HYSPLIT (National Oceanic and Atmospheric Administration Hybrid Single Particle Lagrangian Integrated Trajectory) model suggest that marine air masses would have traveled ~1500 km over 2.5–3 days to reach central Amazonia (Supplementary Fig. 7). The rain records along backward trajectories also indicate that multiple precipitation events occurred during transport which would likely wash out most of the sea salt particles before they could arrive at the sampling site. Hence, these findings support the dominance of locally emitted spores in the Amazon basin during this study. Furthermore, the presence of a substantial amount (48% ± 3%) of coarse mode sodium-rich particles below the ~20 m high forest canopy suggests that local biological emissions contribute significantly to the concentration of sodium-salt particles in the Amazonia.

### Model estimate of fungal spores to sodium budget

To evaluate the geographic distribution and frequency of high fungal spores contributions to airborne sodium mass over the Amazon basin, we conducted model simulations in the Community Earth System Model (CESM1.2.2, see Methods). Briefly, atmospheric emissions and transport simulations were performed using nudging^41^ to recreate the observed meteorology of 2014–2015. To calculate the particulate sodium content from the simulated aerosol mass concentrations, we estimated the sodium dry mass content to be 13% for sodium-rich fungal spores and 30% for sea salt^42^ (see Methods). As an upper bound, we assumed that 70% of fungal spores are sodium rich. We explored the sensitivity of the analysis to this assumption by recalculating key results under the assumption that either 30% or 50% of fungal spores are sodium rich.

**Fig. 3:**
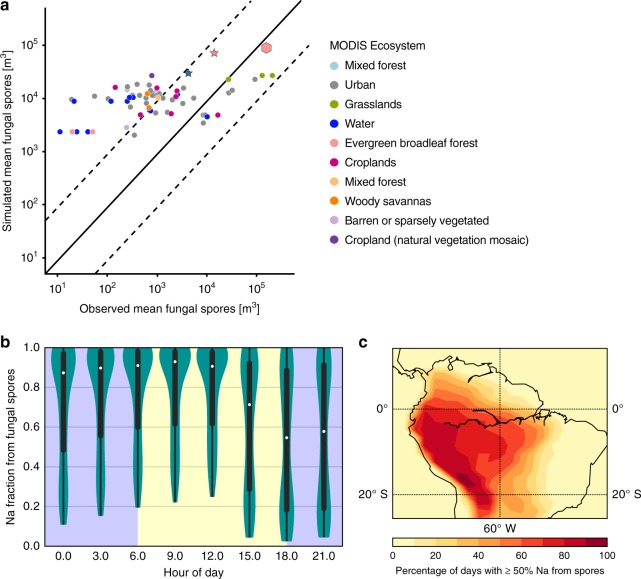
Simulation of sodium contribution from biological particles to total sodium budget in the Amazon area. **a** Comparison of annual-mean model-simulated fungal spore concentrations with previously published observed concentrations at various locations. All model-simulated values are annual means of fungal concentrations simulated using nudged meteorology. Observations are averages over the measurement period for each campaign (mostly between 2 months and 3 years), as previously reported in the literature (a full reference list for the observed spore counts is provided in Supplementary Table 2, together with a map of their geographic locations Supplementary Fig. 8). Dotted lines represent an over- or underestimate of a factor of 10 (10:1 and 1:10, respectively). The pink hexagonal point represents the measured fungal spore concentration in this study, compared with the mean simulated spore concentration from the same time period (26 Jan to 8 Feb 2015). The stars indicate points from literature measurements from tropical rainforests. **b** Distribution of the simulated daily mean sodium fraction contributed by fungal spores during the wet season (Jan–Jun, 2015). Simulated values are obtained from the model grid point nearest to the ZF2 tower (2.84° S 60.0° W), in the model’s lowest (near-surface) layer. The violin plot displays the kernel density estimation of the underlying distribution, along with the median (white dots), 25th–75th percentile (thick vertical bar), and range (thin vertical line). The shaded regions of the plot represent nighttime (blue shading; 1800–0600) and daytime (yellow shading, 0600–1800). **c** Percentage of days when fungal spores contributed at least 50% of estimated total sodium during the wet season, assuming that 70% of fungal spores are sodium rich, containing 13% sodium by mass, and sea salt aerosol is composed of 30% Na by mass. Results for the dry season are shown in Supplementary Fig. 12

Model-estimated and observed fungal spore concentrations in various ecosystems and geographic locations^11^ (Supplementary Fig. 8) are compared in Fig. 3a. Although estimated concentrations were typically higher than observed values, at the measurement site model-estimated fungal spore concentrations were similar to the observed concentrations. Model-estimated and measured sodium mass concentrations at coastal and island sites (Supplementary Fig. 9) are compared in Supplementary Fig. 10. The measurements used in this analysis were all collected at coastal and island locations in the Southern Hemisphere, between 20 and 70 S. Model estimates of sodium mass concentrations are approximately a factor of two higher than observations at many of the measurement sites; measurements at certain remote sites (e.g., Antarctica) are in good agreement with the estimated mass. Both simulated fungal spore and sea salt concentrations exhibited strong temporal variability (Supplementary Fig. 11). Simulated sea salt concentrations, controlled by long-range transport of marine aerosol, are higher during the dry season compared to the wet season. This may be due to stronger wet removal processes hindering long-range transport, and changes in wind patterns during the wet season.

In the model, during periods of low sea salt aerosol concentration, fungal spores typically contribute the majority of the estimated sodium mass. These conditions occur more frequently during the wet season (Fig. 3b) than during the dry season (Supplementary Fig. 12). On average, fungal spores are estimated to contribute 69% of sodium during the wet season. We calculate approximate upper and lower bounds for the fungal spore contribution to the total sodium mass by varying fungal spore mass concentrations by a factor of 10 and sea salt mass concentrations by a factor of 2. On average, fungal spores are estimated to contribute 69% (31–95%) of sodium during the wet season. However, the level of confidence in fungal spore estimates is arguably higher for the Amazon, since the emission parameterization was originally developed based on measurements from the Amazon and other tropical rainforests. Assuming a factor of two uncertainty in the simulated fungal spore concentration, the estimated contribution of fungal spores to simulated sodium mass at the measurement site would range from 51 to 84%. If the assumed percentage of fungal spores that are sodium rich were reduced to 30% or 50%, fungal spores would account for 58% (21–90%) or 65% (27–93%), respectively, of the total simulated sodium mass during the wet season (Supplementary Fig. 13). The model suggests that the fractional contribution of fungal spores to sodium will increase during nighttime and decrease during daytime (Fig. 3b). This may be the combined result of diurnal cycles in emissions, boundary-layer dynamics, and removal processes (e.g., precipitation). Figure 3c presents a regional map of the percentage of days when fungal spores contribute at least 50% of estimated total sodium during the wet season.

**Fig. 4:**
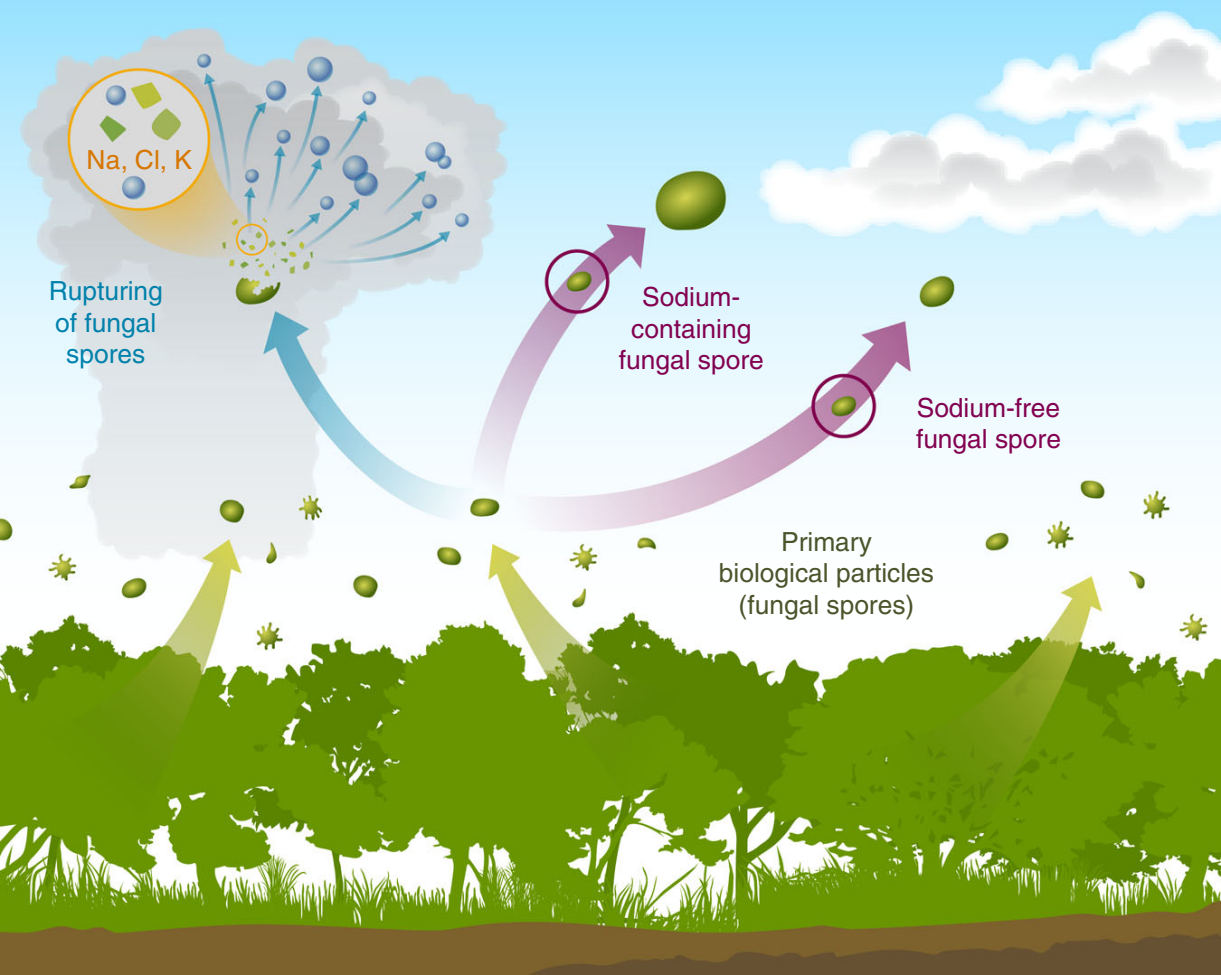
Sources and atmospheric processing of fungal spore particles in the Amazon rainforest. Sodium-containing and sodium-free fungal spore particles are emitted from the Amazon rainforest. Sodium-containing fungal spores exhibit higher hygroscopic growth compared to sodium-free fungal spores. When they are exposed to high humidity conditions, or through cloud processing, fungal spore particles rupture and release submicrometer-to-micrometer size fragments. A substantial fraction of the fragments contain Na, Cl, and K, and appear morphologically similar to dry sea salt particles. These hygroscopic salt fragments can participate in cloud formation

Overall, fungal spores dominated the number fraction of coarse mode aerosols in the Amazonia during nighttime and in below-canopy samples. Half of the coarse mode particles were sodium rich. Previously, sodium-rich particles have been solely attributed to marine aerosol from the Atlantic Ocean. Previous studies in different atmospheric environments show the presence of sodium in the fine mode and suggest potassium as a tracer for anthropogenic sodium^6–8,43,44^. Observations from our study are fundamentally different. First, our study focuses on coarse mode particles. Second, the potassium observed in our study mostly originates from biological particles,^22^ which is confirmed by their unique and distinguishable morphology. These particles were especially abundant during the wet season when biomass burning is not a dominant particle source in the basin. Remarkably, our experimental and modeling results demonstrate that fungal spores emitted from the rainforest biosphere contribute a major fraction to the sodium budget during the wet season, when most marine aerosol particles are removed by wet deposition during transport. Figure 4 illustrates the sources and atmospheric processing of fungal spores. Hygroscopicity experiments indicate that during cloud processing or high humidity spores rupture and release submicrometer-to-micrometer size salt fragments^18^, further contributing to the sodium budget in accumulation mode particles. This work highlights the potential importance of biological particles as a source of sodium-salt particles in widespread tropical forests, and motivates the necessity of studies investigating the chemistry of biological particles in other forested regions of the globe.

## Methods

### Sampling site and sample collection

Samples were collected during the wet season (January and February, 2015) at the ZF2 Tower (02° 35.3517′ S, 60° 06.8333′ W), a pristine rainforest site in Central Amazonia located 40 km North of Manaus margins. Samples were collected through a sampling inlet located at a height of 2 m (below canopy) and 39 m above ground (above canopy), during day and night with 30 L min^−1^ sampling rate. Particles were collected onto 400 mesh transmission electron microscopy (TEM) grids coated with Carbon Type-B films (Ted Pella, Inc.) and silicon nitride membrane substrates (0.5 × 0.5 mm^2^ Si_3_N_4_ window size, 100 nm membrane thickness, 5 × 5 mm^2^ Si frame size; Silson, Inc.). The 10-stage Micro-Orifice Uniform Deposition Impactors™ (MOUDI™; model 110-R, MSP, Inc.) were used for sampling. This study focuses on samples from stages 4 and 5 (size range: 1.0–3.2 µm) where the relative abundance of biological particles is high. Samples were handled with caution. For example, tweezers were cleaned with solvents prior to handling the samples; new gloves were used for handling of samples after each experiment. Examination of blank substrate showed negligible contamination on the sample substrate (Supplementary Fig. 14). Investigation of particles from stages 6 and 7 (size range: 0.32–1.0 µm) showed Na-containing particles in stages 6 and 7 are significantly lower (Na-containing particle <15%) than particles on stages 4 and (Supplementary Fig. 15). This result suggests that a high fraction of sodium-salt particles are indeed from spores that were deposited on stages 4 and 5. Furthermore, sodium content in spores was observed irrespective of different sample preparation and techniques (e.g., SEM/EDX, STXM, and NanoSIMS).

### Chemical imaging of particles

An environmental scanning electron microscope (Quanta 3D model, FEI, Inc.) with an EDAX^TM^ EDX spectrometer and a Si(Li) detector with a 10 mm^2^ active area and an ATW2 window was used under vacuum conditions for imaging and X-ray microanalysis. Particles were imaged using secondary electron (SE) and forward scattered transmitted electron (STE) signals. STE imaging mode was used for particle identification and CC SEM/EDX analysis. X-ray spectrum for each identified particle was acquired at an acceleration voltage of 20 kV and at a beam current of 430 pA for 10 s. The CC SEM/EDX analysis automatically investigates the specified scanning area and detects particles in the specified fields of view. In this way, we can detect ~90% of the particles deposited on the grid. Particles collected on the edges (~5–10%) of the Cu mesh were excluded from the results based on their high Cu background signal. We analyzed ~80% of the particles that were collected onto TEM B-film grids placed on stages 4 and 5 of the impactor. Area equivalent diameters were calculated from the two-dimensional projected area recorded for each individual particle. Particle elemental composition was quantified and is reported in units of atomic fractions. The percentage of sodium content in fungal spores was estimated using the known mass fraction of spores^10^ and carbon/sodium wt% from X-ray microanalysis. Carbon content in spores ranges from 42 to 66%, with an average of 51%^45^. The average carbon/sodium wt% in fungal spores is 4:1. The mass of the spores is estimated from the size distribution of microscopy analysis and assuming a density of 1 gm cm^−3 ^^10,16^.

STXM utilizes a focused soft X-ray beam generated from the synchrotron light source to probe chemical bonding of specific elements of interest within individual particles. STXM images are obtained by raster scanning the sample at fixed photon energy and recording intensities of the transmitted X-rays at each pixel. The optical density (–ln(*I*(_d_)/*I*_0_) is calculated based on the measured intensity (*I*_d_) using Beer–Lambert’s law^46^. Transmission intensity through a particle free region of the substrate is used to obtain *I*_0_.

The hygroscopicity of biological particles was investigated using a temperature-controlled cooling stage in the environmental SEM to estimate the area growth factor (ratio of wet-to-dry diameters) and a microreactor for in situ STXM hydration experiments^27^ to estimate the mass growth factor (ratio of wet-to-dry oxygen mass).

NanoSIMS (CAMECA 50L, Gennevilliers Cedex, France) was used to perform imaging of Na+ ions. NanoSIMS provides high lateral resolution. Samples were coated with a thin layer of Au to minimize charging and non-equilibrium sputtering effects during analysis. A 16 keV O^−^ primary ion beam was used for analysis. Prior to data collection, samples were pre-sputtered with about 10^16^ O^−^ ions cm^−2^. Analyses of 15 μm × 15 μm and 256 px × 256 px were performed with a dwell time of 13.5 ms px^−1^. Beam diameter was estimated to be about 250 nm.

### Community atmosphere model description

Fungal spore emissions were calculated according to the global model parameterization of Heald and Spracklen^47^. While this parameterization has substantial uncertainties, it was developed on the basis of observed mannitol concentrations from various continental locations and seasons; about half of these observations were from PM2.5 aerosol collected in the tropical Brazilian rainforest during the wet season, where concentrations were 2–3 times higher than those reported at extratropical locations^10^. Therefore, we believe the parameterization can be used with greater confidence in the Amazonian rainforest, particularly during the wet season, than at other times and locations.

A simulation for present-day conditions was performed using the Community Atmosphere Model (CAM5)^48^, which is the atmosphere component of the CESM (version 1.2.1)^49^. The model was configured at a horizontal resolution of 1.9° latitude by 2.5° longitude, with 30 vertical layers ranging from the surface to 2.26 hPa. The simulation was performed from March 2014 to March 2015 with constrained meteorology^50^,where model winds are nudged toward the MERRA reanalysis^51^ with a relaxation time of 6 h^41^.

The model tracks the emissions and concentrations of six chemical species of aerosol: sea salt, dust, sulfate, secondary organic aerosol, black carbon, and continental primary organic aerosol. Aerosol transport and microphysics, including nucleation, condensation, and coagulation, is calculated within a modal aerosol module with three size classes (MAM3)^52^, including the Aitken mode (dry diameter size range of 0.02–0.08 μm), accumulation mode (0.08–1.0 μm), and coarse mode (1.0–10.0 μm). Sea salt aerosol emission follows Mårtensson et al.^53^ for small particles (diameter <2.8 μm), while emissions of larger particles (diameter >2.8 μm) follow Monahan^54^. The simulated sea salt budget was evaluated in Liu et al.^52^ and falls within the AeroCom values^55^; the modeled global sea salt emission flux is within the range of 2200~118,000 Tg yr^−1^ using different sea salt source functions. A detailed description of the model’s aerosol removal processes can be found elsewhere^48^.

Fungal spores were emitted into the model’s coarse mode, and are removed by wet and dry deposition in the same manner as other aerosol species. Upon emission both fungal spore mass and coarse mode number are increased, with the mass-to-number ratio for emissions determined by assuming that fungal spores have 4 μm diameter upon emission. The model’s coarse mode particle number concentration also includes number contributions from other aerosol species which are treated as internally mixed within the coarse mode, and the particle diameter is calculated dynamically by the aerosol microphysics routines from the total coarse mode aerosol mass and number, and a fixed geometric width. Fungal spores were assigned the same optical properties as the model’s organic carbon tracer which is close to the observed refractive indices of fungal spores (1.4-0i)^20^. Fungal spores were assigned a material density of 1 g cm^−3^, which is commonly used in literature^10,16^. Fungal spores were assigned a hygroscopicity parameter (*κ*) of 0.1, similar to that of pollen grains^56^. The observed overall *κ* value for ambient below-canopy aerosol in the Amazon is 0.22 ± 0.05 in the accumulation mode, with an overall mean value of *κ* = 0.17 ± 0.06^57^.

### Code availability

The CAM5 model source code and input datasets are available at http://www.cesm.ucar.edu/. The code modifications to CAM5 to introduce fungal spore aerosol with emissions following Heald and Spracklen^47^, and to enable nudging of meteorological fields, are currently available at http://portal.nersc.gov/project/acme/sburrows/fungal_emissions/.

## Electronic supplementary material

Supplementary Information

Peer Review File

## Data Availability

All relevant data are available from the authors.
