# Supplementary material for: Fungal spores as a source of sodium salt particles in the Amazon basin
Source: Nat Commun. 2018 Nov 19;9:4793. doi: 10.1038/s41467-018-07066-4 (PMC6242827; doi:10.1038/s41467-018-07066-4)
Supplement: Supplementary file 1 — Supplementary Information [file 41467_2018_7066_MOESM1_ESM.pdf]

Supplementary information

Fungal spores as a source of sodium salt particles in the Amazon basin

China et al.

Swarup China[1], Susannah M. Burrows[2], Bingbing Wang[3], Tristan H. Harder[4,5,‖], Johannes Weis[4,5], Meryem Tanarhte[6], Luciana V. Rizzo[7], Joel Brito[8,#], Glauber G. Cirino[9], Po-Lun Ma[2], John Cliff[1], Paulo Artaxo[10], Mary K. Gilles[4], Alexander Laskin[10,*]

[1]*William R. Wiley Environmental Molecular Sciences Laboratory, Pacific Northwest National Laboratory, Richland, WA 99354, USA*

[2]*Atmospheric Science and Global Change Division, Pacific Northwest National Laboratory, Richland, WA 99354, USA*

[3]*State Key Laboratory of Marine Environmental Science, College of Ocean and Earth Science, Xiamen University, Xiamen 361102, China*

[4]*Chemical Sciences Division, Lawrence Berkeley National Laboratory, Berkeley, CA 94720, USA*

[5]*Department of Chemistry, University of California, Berkeley, CA 94720, USA.*

[6]*Max Planck Institute for Chemistry, Mainz 55128, Germany*

[7]*Universidade Federal de Sao Paulo, Diadema, SP, Brazil*

[8]*Institute of Physics, University of São Paulo, Rua do Matão 1371, CEP 05508-090, São Paulo, S.P., Brazil*

[9]*Geosciences Institute, Federal University of Para, Belem, 66075-110, Brazil*

[10]*Department of Chemistry, Purdue University, West Lafayette, IN 47907, USA*

[‖]*present address: Physikalisches Institut, Universität Würzburg, Am Hubland, 97074 Würzburg, Germany*

[#]*present address: IMT Lille Douai, Univ. Lille, SAGE, 59000 Lille, France*

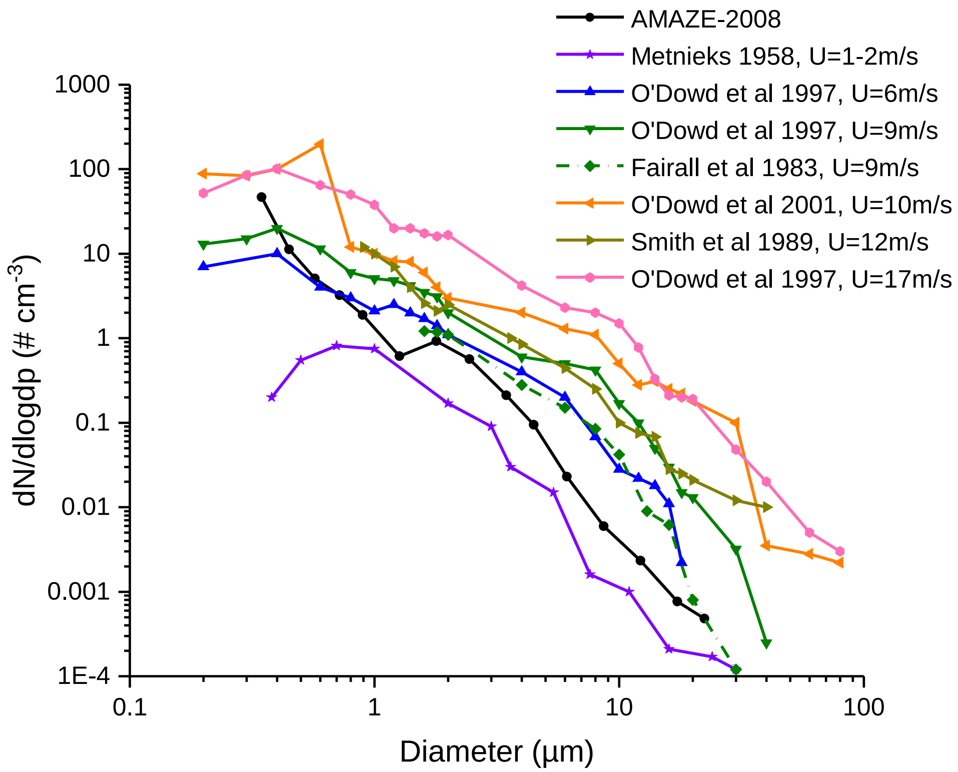

Supplementary Figure 1. Comparison of coarse particles in Amazonia size distribution with sea salt particles from different marine areas. Size distribution of sea salt particles for maritime air masses over different coastal sites (Metnieks[1], 1958 in Ireland; O'Dowd et al[2] 1997 in North East Atlantic coastal site; Fairall et al[3] 1952 in California coastal area; and Smith et al[4] 1989 in North East Atlantic coastal site) at several wind speeds, U. Size distribution data is from Lewis and Schwartz[5]. Average particle concentrations for the wet season from Amazonia (black line) were measured using an optical particle counter during the Amazonian Aerosol Characterization Experiment (AMAZE-08) field campaign during 2008.

Supplementary Table 1. Aerosol sample identifications, local (AMT) sampling time, and duty cycle. Number of particles (N) analyzed and estimated margin of error (ME) at a given confidence interval (99%) applying binomial distribution[6].

Sample #	Canopy	Sampling start	Sampling end	Duty cycle (%)	N	ME
1A	Above	01/28/2015 12:05 PM	01/28/2015 2:30 PM	100	580	4.8
1B	Below	01/28/2015 12:05 PM	01/28/2015 2:30 PM	100	472	5.4
2A	Above	01/28/2015 7:00 PM	01/29/2015 5:30 AM	75	305	6.7
2B	Below	01/28/2015 7:00 PM	01/29/2015 5:30 AM	75	366	6.1
3A	Above	01/30/2015 11:30 AM	01/30/2015 2:00 PM	100	180	8.7
3B	Below	01/30/2015 11:30 AM	01/30/2015 2:00 PM	100	220	7.9
4A	Above	01/30/2015 09:00 AM	02/02/2015 5:00 PM	75	240	7.5
4B	Below	01/30/2015 09:00 AM	02/02/2015 5:00 PM	75	320	6.5
5A	Above	02/02/2015 08:00 PM	02/04/2015 05:00 AM	75	524	5.1
5B	Below	02/02/2015 08:00 PM	02/04/2015 05:00 AM	75	614	4.7

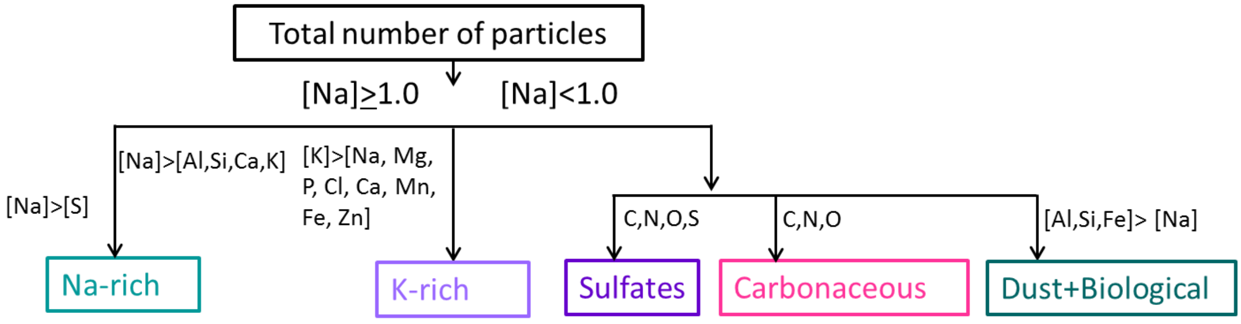

Supplementary Figure 2. Particle-type classification scheme. Total particles were classified into five groups based on their elemental compositions from CCSEM/EDX data.

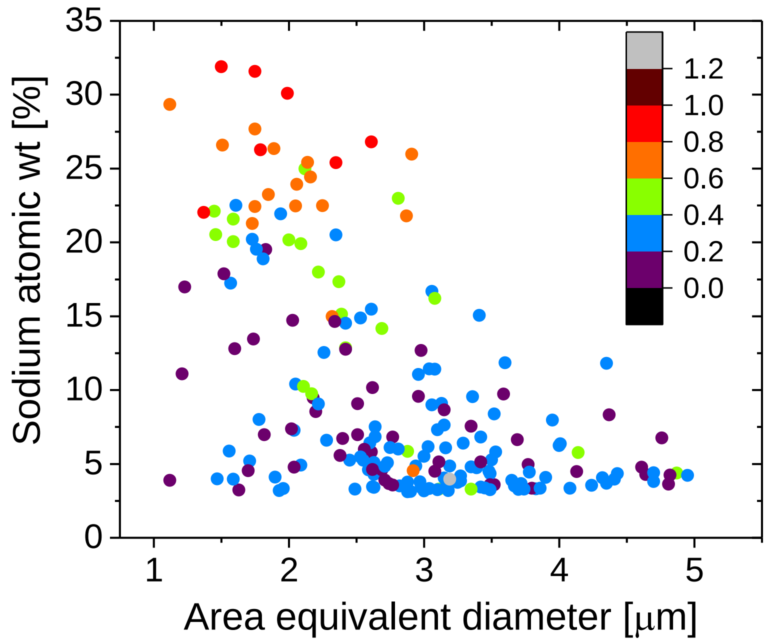

Supplementary Figure 3. Size dependence of sodium fraction in individual biological particles. Atomic weight percent of sodium in individual biological particles. Color bar indicates Cl/Na atomic ratio. For fresh particles, Cl/Na atomic ratio would be close to 1 for fresh sea salt particles.

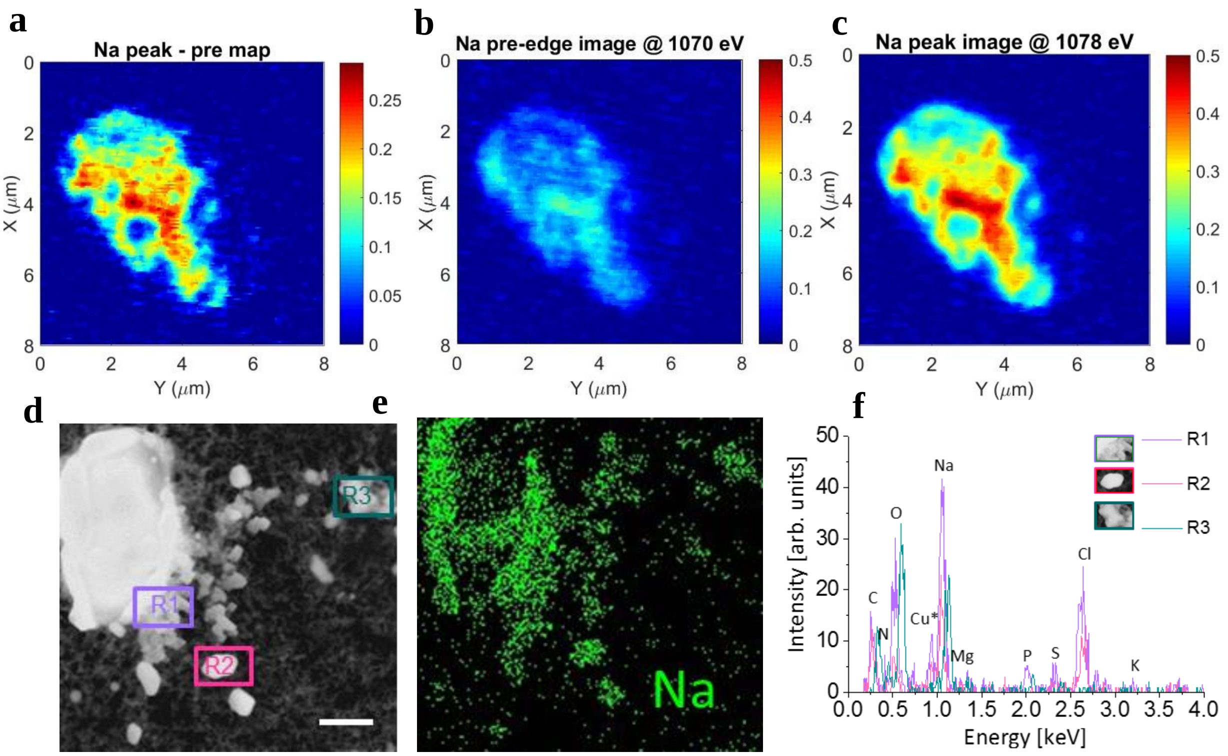

Supplementary Figure 4. Chemical composition of ruptured fungal spores. Scanning transmission X-ray microscopy images at a) Na map (optical density difference between Na K peak and pre-edge), b) Na K pre-edge (1070 eV) and c) peak (1078 eV) and. Color bars represent optical density. SEM image d) of a ruptured fungal spore and its fragments after prolonged water vapor exposure (8 hrs); e) elemental Na map; f) the EDX spectra acquired over three different regions of the particle (indicated by boxes in the SEM image) showing NaCl composition of the fragmented particles. Energies for three different regions are slightly offset for better visualization of the spectra.

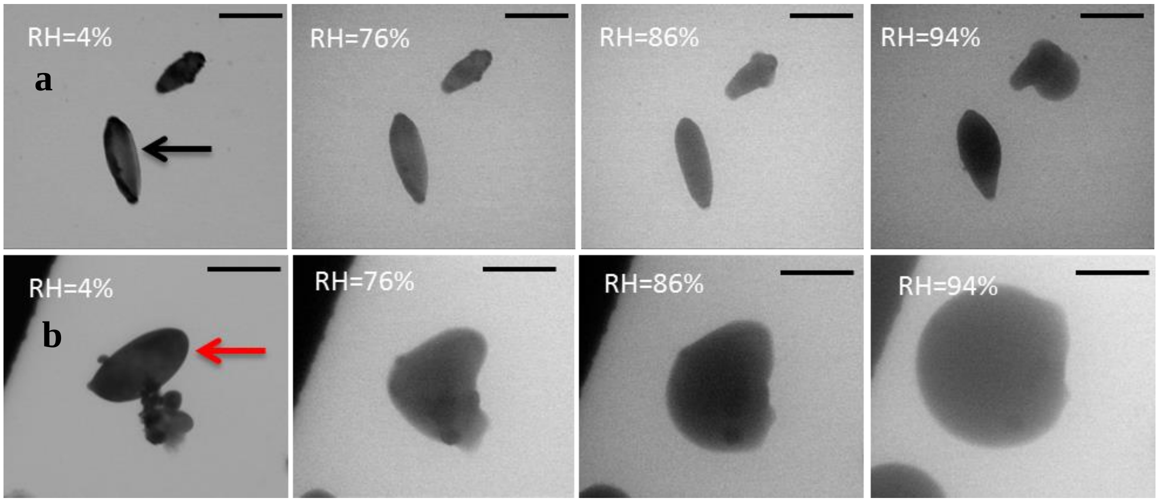

Supplementary Figure 5. Dynamic ESEM images of biological particles during hydration experiments. An environmental scanning electron microscope (ESEM, Quanta 3D model, FEI, Inc.) with a Peltier cooling stage was used for hydration experiments. a) Sodium-free biological particle is indicated by the black arrow and b) a sodium-containing biological particle is indicated by a red arrow. First, the sample on TEM grids was loaded onto the cooling stage at 278 K. Next, by controlling the water vapor in the SEM chamber, the relative humidity (RH) was increased. Scale bars are 5 µm.

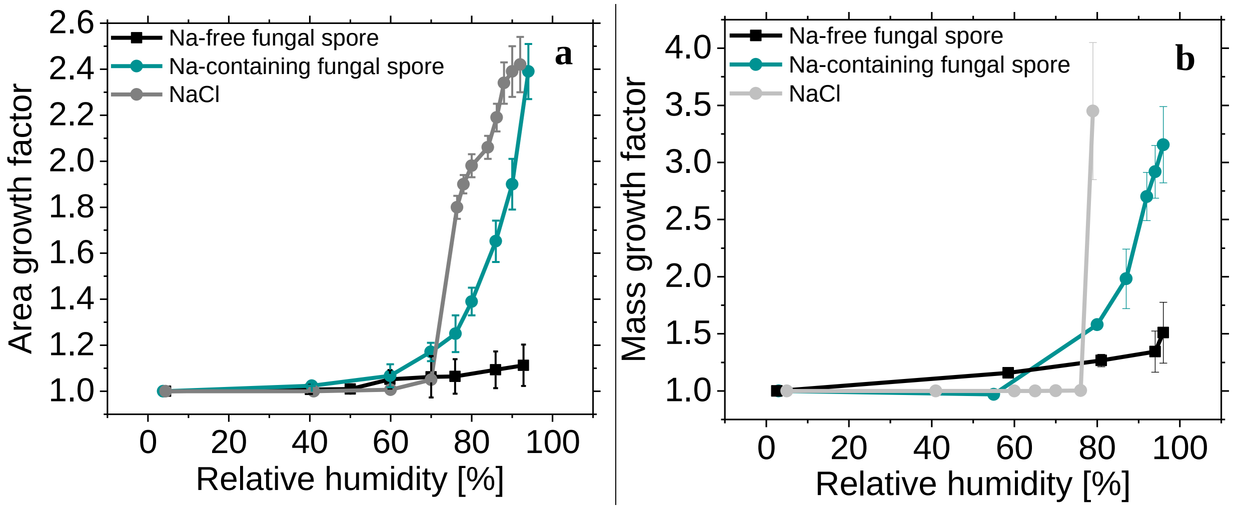

Supplementary Figure 6. Hygroscopic growth factors of fungal spore. a) Area equivalent diameter growth factors (D_{wet}/D_{dry}) of sodium-free fungal spore (black line), sodium-containing fungal spore (red line) from ESEM experiments. Gray line indicates the growth factor of laboratory generated NaCl particles. b) Mass growth factor of sodium-free fungal spore and sodium-free fungal spore particles using in situ scanning transmission X-ray microscopy (STXM) humidification experiments[7].

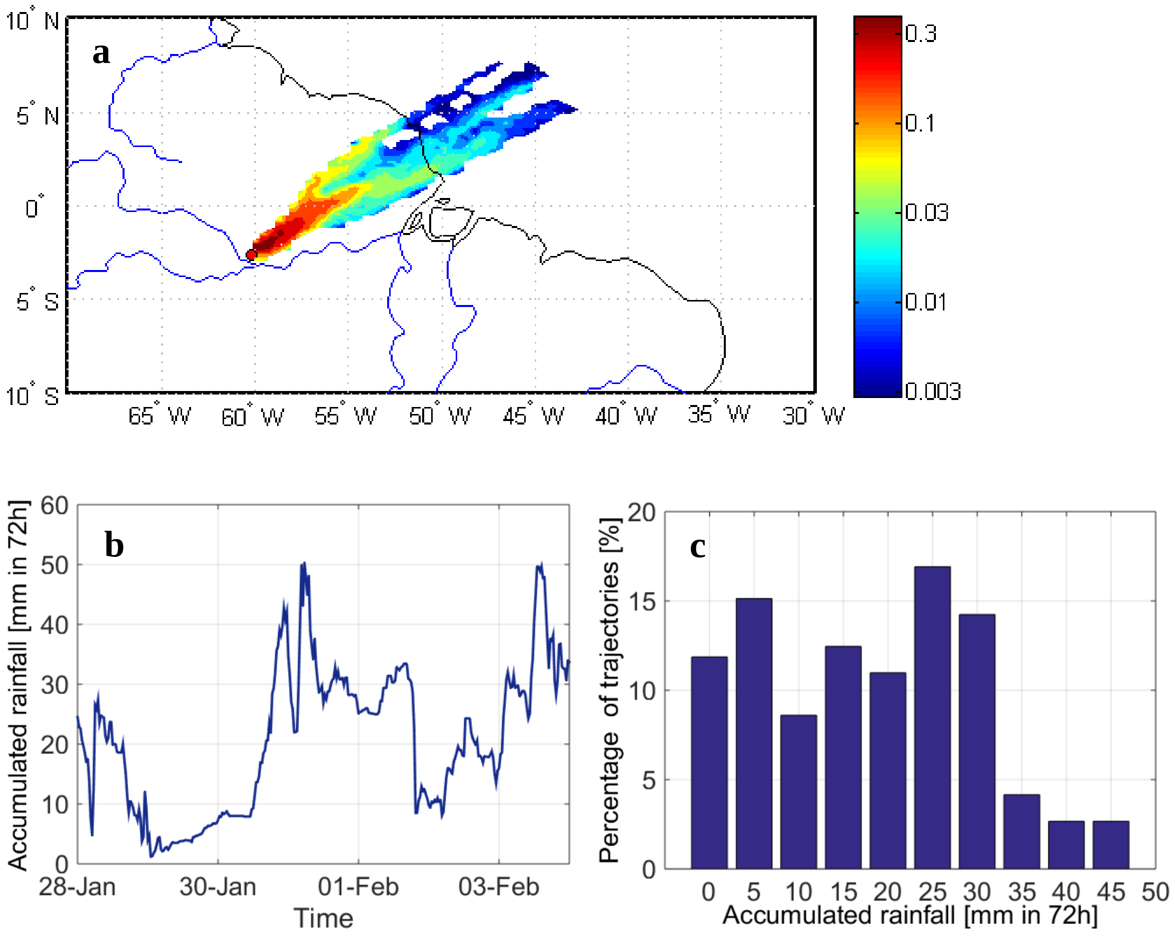

Supplementary Figure 7. Airmass transport patterns during the sampling period. Backward trajectory analysis using the Hybrid Single-Particle Lagrangian Integrated Trajectory (HYSPLIT) model. Backtrajectories ending at the at the ZF2 site were calculated every 30 minutes within the sampling period (28 January-04 February, 2015) using HYSPLIT model, comprehending 337 trajectories in total. Backtrajectory duration was 72 h, final height was 100 m, and 1 degree GDAS (Global Data Assimilation System) meteorological fields were used in the calculations. **a,** Density of 72h backtrajectories ending at the ZF2 site (symbolized by a red circle) in central Amazon basin sampling site. The color bar represents the fraction of the 337 trajectories passing over a region. **b,** Time series of rainfall integrated for each backtrajectory. **c,** Histogram of rainfall integrated for each backtrajectory. Approximate travel time of marine air masses to the sampling site was ~2.5-3 days.

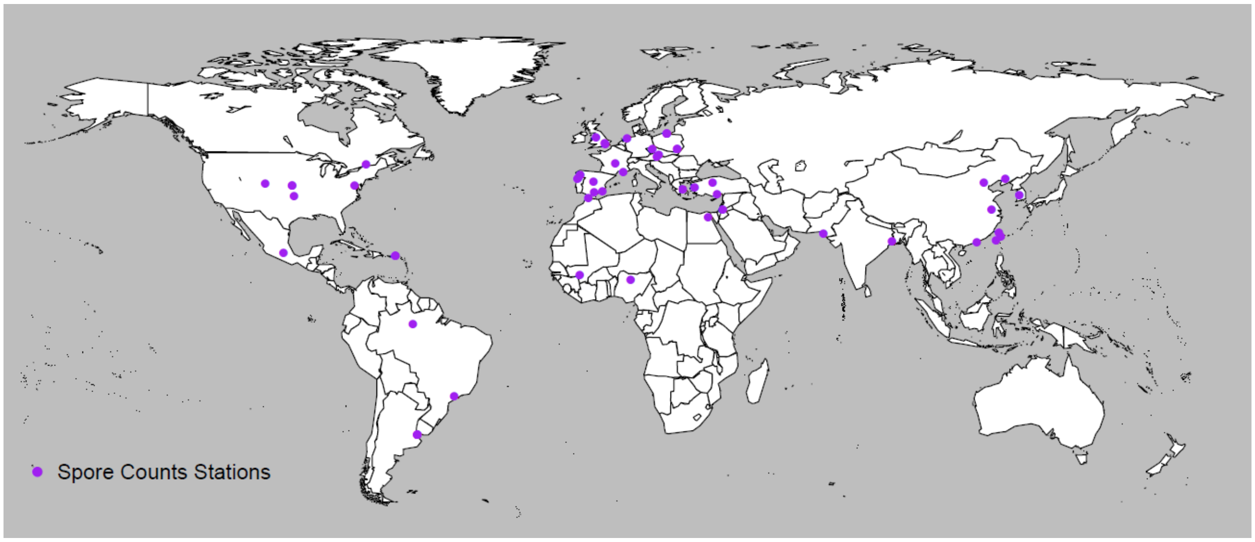

Supplementary Figure 8. Global map of spore counts and measurement site. Purple dots represent the measurement where spore counts are available and used in Fig. 3 and Supplementary Table 2.

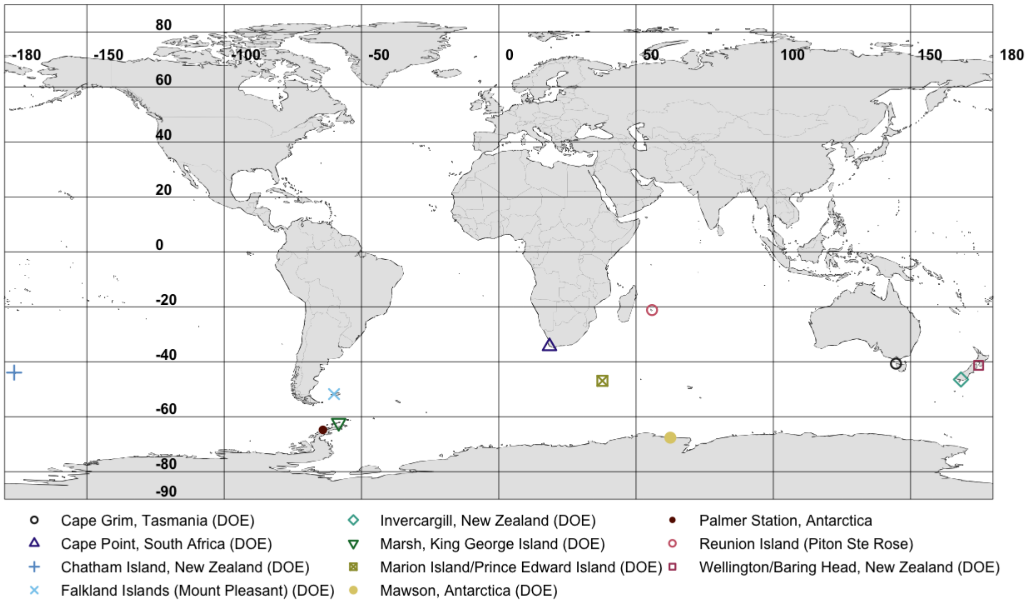

Supplementary Figure 9. Global map of sodium measurement sites, from the AEROCE/SEAREX. Symbols represent locations where measurements of the atmospheric aerosol sodium mass concentration are available and are compared with model-estimated sodium mass concentration. Note that many sites in this network sampled aerosol only for certain wind directions (sector sampling); sector-sampled sites were excluded from this comparison to remove impacts of sampling bias.

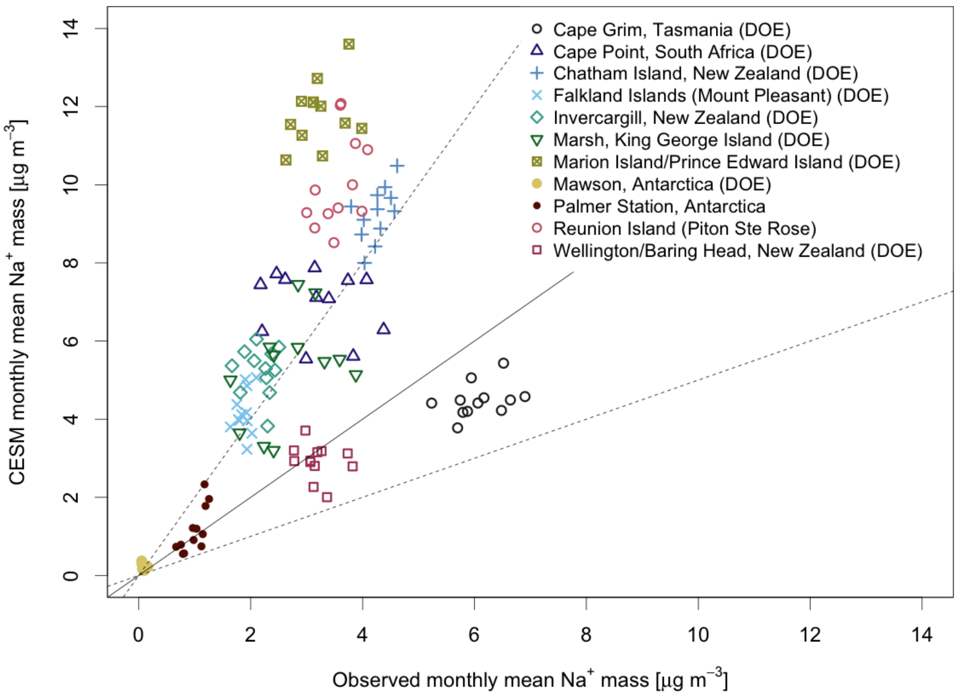

Supplementary Figure 10. Comparison of climatological monthly mean model-simulated and observed sodium mass from sea-salt. Observations are multi-year climatological monthly means of all available years from the AEROCE/SEAREX observational dataset (http://aerocom.met.no/databenchmarks.html)[8-10], collected at sampling sites shown in Supplementary Figure 8. Note that to ensure a representative comparison, this analysis has excluded sites in the measurement network that sampled aerosol only for certain wind directions (sectored sampling). The solid line represents the 1:1 line, and the dashed lines represent an over- or under-estimate of a factor of two (2:1 and 1:2).

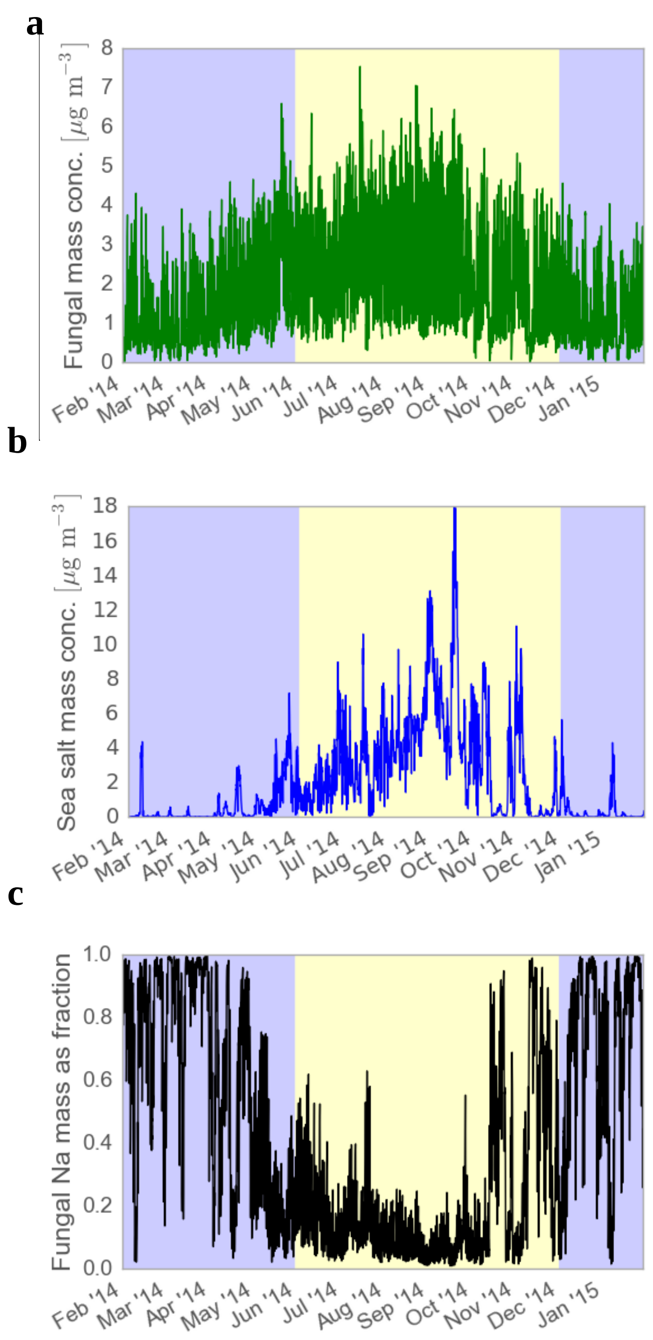

Supplementary Figure 11. Modeled time series of Na-containing particles from fungal spores and transported marine aerosol for March 2014 – Feb 2015. (a) Simulated fungal spore mass concentration. (b) Simulated sea salt mass concentration. (c) Mass fraction of Na, estimated from modeling results in airborne particles attributed to fungal spores. Background shading indicates season (blue for wet, yellow for dry). Results are from the nearest modeled grid point to the ZF2 tower, located at 2.84° S 60.0° W, in the model's lowest (near-surface) layer.

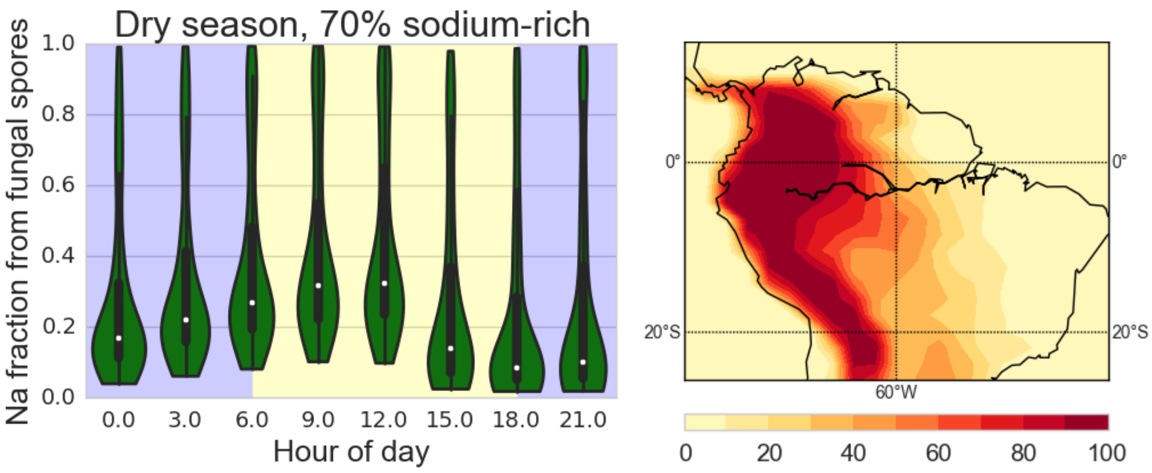

Supplementary Figure 12. Simulation of sodium contribution of biological particles to total sodium budget during dry season. Sodium fraction from fungal spores during dry season at the model's nearest grid point to the sampling site. Figure 3 shows similar data for the wet season. White dots represent the median, thick lines represent 25-75 percentile, and thin vertical lines represent the range of minimum-to-maximum values; violin plot represents a kernel density distribution of the sodium fraction values. Background shading indicates time of day, blue represents night [1800-0600] and yellow represents day [0600-1800].

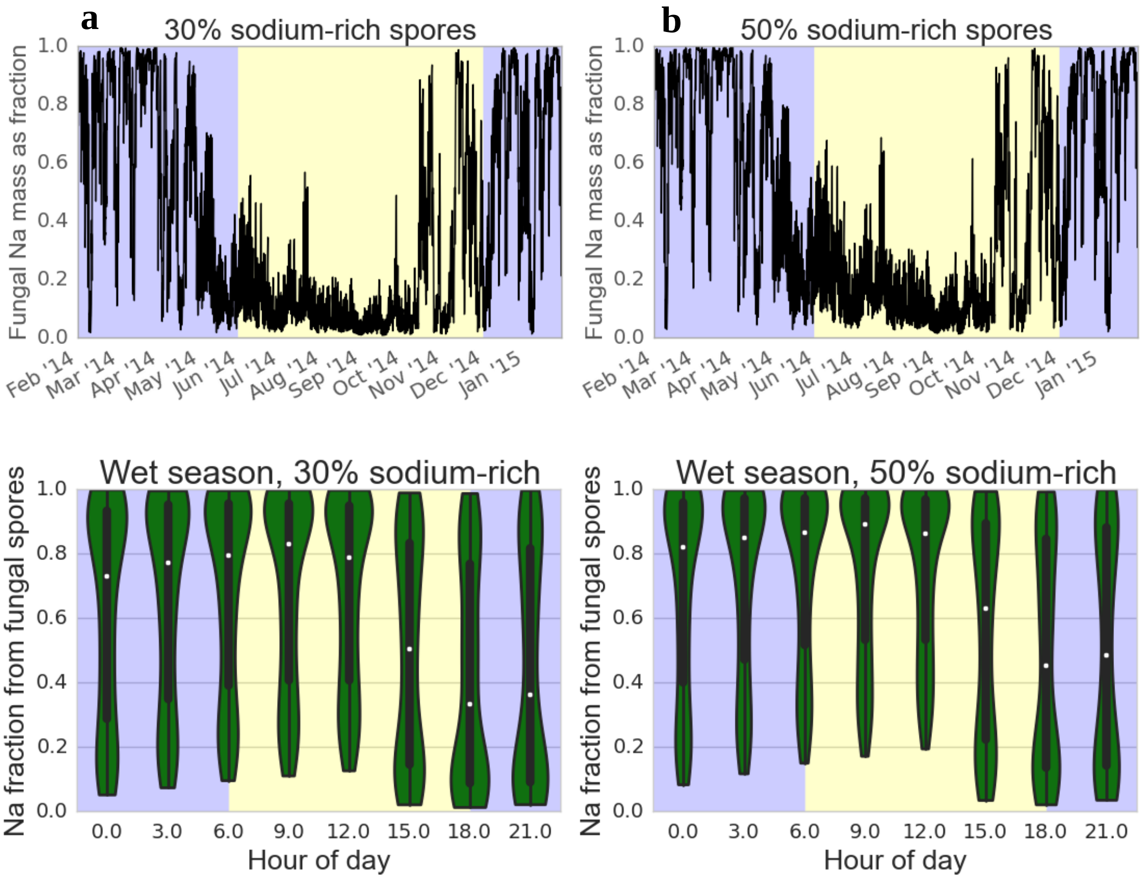

Supplementary Figure 13. Modeled time series of sodium mass contribution from fungal spores assuming different number fractions of sodium-rich spores. Modeled time series (March 2014 – Feb 2015) of sodium mass percentage contributed by fungal spores assuming a) 30% and b) 50% of spores are sodium-rich.

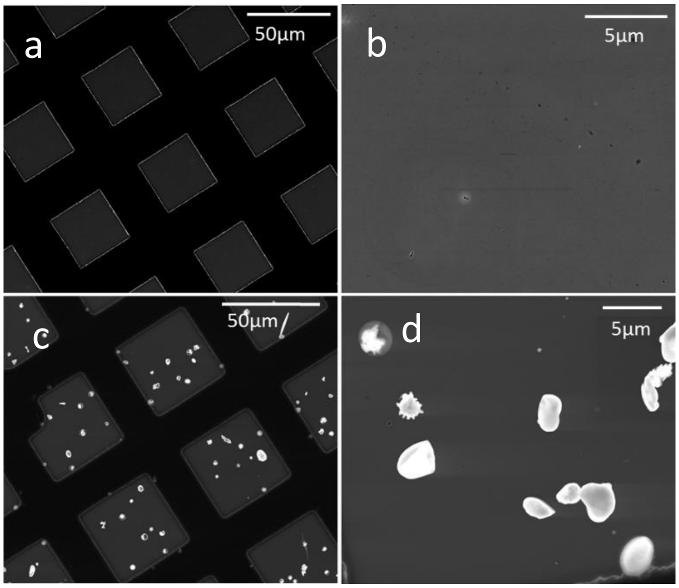

Supplementary Figure 14. Investigation of particle contamination. SEM images show a) low magnification and b) high magnification image of blank sample; c) low magnification and d) high magnification image of collected sample.

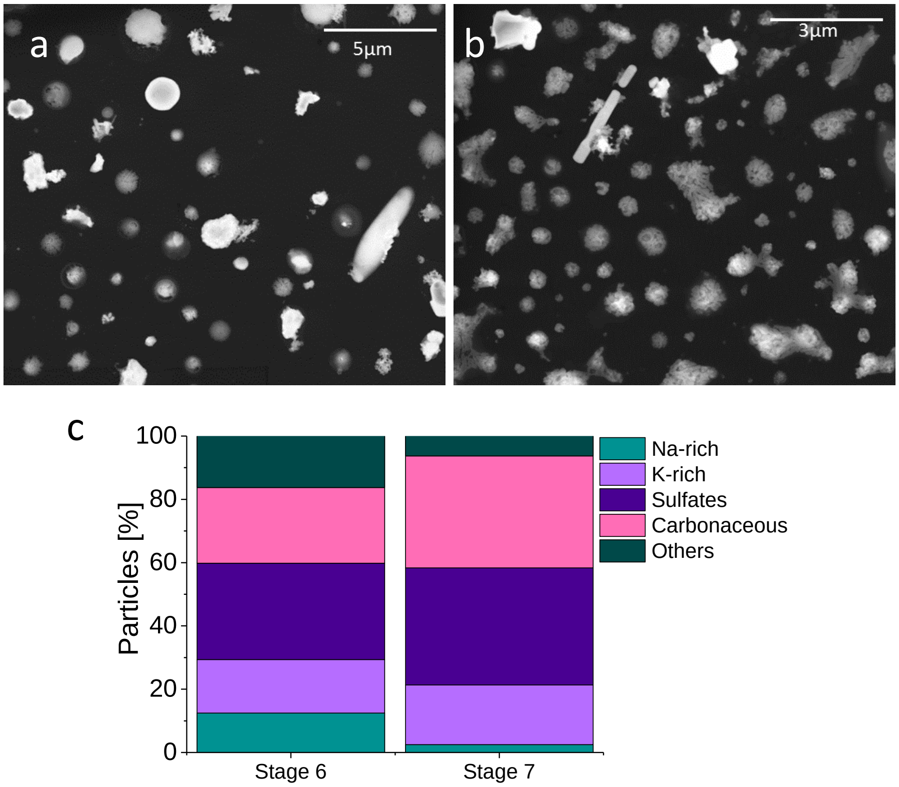

Supplementary Figure 15. Fraction of Na-rich particles in smaller size (stages 6 and 7) particles. Representative SEM images of particles collected on a) stage 6 (0.56-1.0 µm), b) stage 7 (0.32-0.56 µm), c) number fraction of different particle classes for particles collected on stages 6 and 7. Fraction of Na-rich particles is <15%.

Supplementary Table 2. Observed mean fungal spore concentrations at various ecosystem and geographical locations. Observed concentration (Obs. Conc.), minimum concentration (Min. Conc.), maximum concentration (Min. Conc.) are reported in number of spore per m^{-3}.

No#	Longitude	Latitude	Altitude	Start date	End date	Obs Conc.	Min. Conc.	Max. Conc.	Ecosystem	Ref. no
1	2,95	45,76	1465	04.03.2003	06.03.2003	222	53	390	Mixed forest	[11]
2	15,76	47,71	1644	01.03.2000	30.03.2000	773	104	2031	Mixed forest	[12]
3	6,6	53,16	5	01.01.1981	31.12.1983	258	42	1225	Urban	[13]
4	-73,75	45,46	0	16.10.2002	16.10.2002	615	492	738	Urban	[14]
5	-0,11	51,5	64	01.06.1961	31.10.1961	7500	2000	13000	Urban	[15]
6	-2,98	53,4	92	01.06.1961	31.10.1961	9000	3000	15000	Urban	[15]
7	5,36	43,28	0	01.01.1962	31.12.1962	69	3	411	Urban	[16]
8	-59,4	-1,91	174	21.07.2001	21.07.2001	12476	4764	20188	Evergreen broadleaf forest	[17]
9	14,41	50,08	0	01.10.1988	15.12.1988	17	NA	NA	Urban	[18]
10	7,63	11,18	690	01.07.1959	30.07.1959	128	20	237	Croplands	[19]
11	-46,63	-23,55	0	01.03.2007	31.03.2007	3768	NA	NA	Urban	[20]
12	-5,36	35,56	250	01.01.2009	31.12.2012	1434	177	12082	Urban	[21]
13	22	50,03	0	01.01.2001	31.12.2002	2163	NA	NA	Urban	[22]
14	22,08	50,03	0	01.04.2001	31.12.2002	2119	NA	NA	Croplands	[22]
15	18,8	54,6	0	01.01.1998	31.03.1998	105	0	1000	Water	[23]
16	18,8	54,6	0	01.07.1998	30.09.1998	223	0	600	Water	[23]
17	18,85	54,52	0	02.08.1995	17.09.1995	19	0	122	Water	[23]
18	-8,38	41,51	5	01.01.2005	31.12.2007	934	NA	NA	Mixed forest	[24]
19	-8,65	41,18	20	01.01.2005	31.12.2007	531	NA	NA	Urban	[24]
20	-7,85	42,33	0	01.01.2002	31.12.2002	964	NA	NA	Urban	[25]
21	-64,73	18,33	0	01.07.2000	30.07.2000	45	30	60	Evergreen broadleaf forest	[26]
22	-64,79	18,33	0	18.07.2000	08.08.2001	21,24	18,25	61	Water	[27]
23	34,25	36,6	0	18.03.2002	22.10.2002	1702	100	8510	Croplands	[28]
24	114,26	22,33	0	01.09.2002	30.04.2003	269,5	28,5	1963	Forest	[29]

25	-104,86	39,75	0	01.01.1987	31.12.1995	409	NA	NA	Urban	[30]
26	-96,56	39,18	0	01.04.1953	30.10.1953	24499	837	48162	Urban	[31]
27	-96,56	39,18	0	01.12.1953	30.03.1954	715	170	1261	Urban	[31]
28	-95,93	36,11	0	01.01.2002	31.12.2002	24121	53	48188	Urban	[32]
29	-57,95	-34,91	15	01.07.2000	15.09.2000	284	NA	2000	Urban	[33]
30	-57,95	-34,91	15	15.09.2000	15.12.2000	395	NA	2000	Urban	[33]
31	-57,95	-34,91	15	15.12.2000	15.03.2001	814	NA	3478	Urban	[33]
32	-57,95	-34,91	15	15.03.2001	30.06.2001	715	NA	4763	Urban	[33]
33	-3,58	37,18	0	01.01.1994	31.12.1994	832	NA	NA	Urban	[34]
34	32,86	39,93	15	01.01.1990	31.12.1990	2917	17	5817	Urban	[35]
35	-0,35	51,81	0	04.05.1954	30.09.1954	11500	6400	10000	Croplands	[36]
36	121,1	25,05	0	01.03.2003	31.12.2004	2255	NA	NA	Water	[37]
37	120,2	23	0	01.12.2000	30.04.2001	28683	NA	NA	Urban	[38]
38	-8	12,65	0	01.02.2001	31.03.2001	225	80	370	Urban	[39]
39	127,38	36,35	0	01.01.1995	31.12.1995	3014	100	5929	Urban	[40]
40	121,6	23,96	0	01.04.1993	31.03.1996	4839	NA	NA	Urban	[41]
41	36,1	32,01	0	01.12.2008	30.09.2009	7541	3066	12017	Urban	[42]
42	23,71	37,98	30	01.01.1998	31.12.2001	1055	60	6328	Urban	[43]
43	-99,18	19,31	0	01.08.1988	28.02.1989	351	45	3195	Urban/shrub	[44]
44	-0,98	37,6	0	01.01.1994	31.12.1999	655	10	1301	Croplands	[45]
45	67	24,85	0	01.01.2010	31.12.2010	310	157	469	Urban	[46]
46	-3,75	40,45	0	01.01.2003	31.12.2003	609	166	1614	Urban	[47]
47	16,36	48,2	0	01.01.2002	31.12.2002	49	NA	NA	Forest	[48]
48	31,55	29,86	0	01.01.2006	29.02.2007	216	101	331	Urban	[49]
49	116,38	39,91	0	01.06.2003	30.05.2004	1164	23	13959	Urban	[50]
50	118,76	32,05	0	21.03.1998	14.07.1998	655	NA	NA	Urban	[51]
51	123,06	41,13	0	01.12.1990	30.11.1991	1797	NA	NA	Urban	[52]
52	-77,33	39,23	0	01.09.1978	01.12.1980	695	4	6885	Suburban	[53]
53	27,41	38,61	74	01.01.2004	31.12.2005	541	415	780	Urban	[54]
54	88,16	22,66	10	01.10.1996	30.09.1998	879	119	1639	Croplands	[55]

References

1 Metnieks, A. L. The size spectrum of large and giant sea-salt nuclei under maritime conditions. *Geophys. Bull.* **15**, 1-50 (1958).

2 O'Dowd, C. D., Smith, M. H., Consterdine, I. E. & Lowe, J. A. Marine aerosol, sea-salt, and the marine sulphur cycle: a short review. *Atmospheric Environment* **31**, 73-80, doi:http://dx.doi.org/10.1016/S1352-2310(96)00106-9 (1997).

3 Fairall, C. W., Davidson, K. L. & Schacher, G. E. An analysis of the surface production of sea-salt aerosols. *Tellus B* **35B**, 31-39, doi:10.1111/j.1600-0889.1983.tb00005.x (1983).

4 Smith, M. H., Consterdine, I. E. & Park, P. M. Atmospheric loadings of marine aerosol during a Hebridean cyclone. *Quarterly Journal of the Royal Meteorological Society* **115**, 383-395, doi:10.1002/qj.49711548610 (1989).

5 Lewis, E. R. & Schwartz, S. E. *Sea salt aerosol production: mechanisms, methods, measurements, and models-A critical review.* Vol. 152 (American Geophysical Union, 2004).

6 Levy, P. S. & Lemeshow, S. *Sampling of populations: methods and applications*. (John Wiley & Sons, 2013).

7 Piens, D. S. *et al.* Measuring Mass-Based Hygroscopicity of Atmospheric Particles through in Situ Imaging. *Environmental Science & Technology* **50**, 5172-5180, doi:10.1021/acs.est.6b00793 (2016).

8 L., S. D. *et al.* Marine biogenic and anthropogenic contributions to non-sea-salt sulfate in the marine boundary layer over the North Atlantic Ocean. *Journal of Geophysical Research: Atmospheres* **107**, AAC 3-1-AAC 3-21, doi:doi:10.1029/2001JD000970 (2002).

9 Gong, S. *et al.* Modeling sea-salt aerosols in the atmosphere: 2. Atmospheric concentrations and fluxes. *Journal of Geophysical Research: Atmospheres* **102**, 3819-3830 (1997).

10 Li-Jones, X. & Prospero, J. Variations in the size distribution of non-sea-salt sulfate aerosol in the marine boundary layer at Barbados: Impact of African dust. *Journal of Geophysical Research: Atmospheres* **103**, 16073-16084 (1998).

11 Amato, P. *et al.* Microbial population in cloud water at the Puy de Dôme: Implications for the chemistry of clouds. *Atmospheric Environment* **39**, 4143-4153, doi:10.1016/j.atmosenv.2005.04.002 (2005).

12 Bauer, H. *et al.* The contribution of bacteria and fungal spores to the organic carbon content of cloud water, precipitation and aerosols. *Atmos Res* **64**, 109-119, doi:10.1016/S0169-8095(02)00084-4 (2002).

13 Beaumont, F., Kauffman, H. F., van Der Mark, T. H., Sluiter, H. J. & de Vries, K. Volumetric aerobiological survey of conidial fungi in the North-East Netherlands. I. Seasonal patterns and the influence of meteorological variables. *Allergy: European Journal of Allergy and Clinical Immunology* **40**, 173-180 (1985).

14 Côté, V., Kos, G., Mortazavi, R. & Ariya, P. A. Microbial and "de novo" transformation of dicarboxylic acids by three airborne fungi. *Science of the Total Environment* **390**, 530-537, doi:10.1016/j.scitotenv.2007.10.035 (2008).

15 Davies, R. R., Denny, M. J. & Newton, L. M. A COMPARISON HETWEEN THE SUMMER AND AUTUMN AIR-SPORAS AT LONDON AND LIVERPOOL. *Allergy* **18**, 131-147, doi:10.1111/j.1398-9995.1963.tb03156.x (1963).
16 Di Giorgio, C. *et al.* Atmospheric pollution by airborne microorganisms in the city of Marseilles. *Atmospheric Environment* **30**, 155-160, doi:10.1016/1352-2310(95)00143-M (1996).
17 Elbert, W., Taylor, P. E., Andreae, M. O. & Poschl, U. Contribution of fungi to primary biogenic aerosols in the atmosphere: wet and dry discharged spores, carbohydrates, and inorganic ions. *Atmospheric Chemistry and Physics* **7**, 4569-4588 (2007).
18 Fišar, Z., Hýsek, J. & Binek, B. Quantification of airborne microorganisms and investigation of their interactions with non-living particles. *International Journal of Biometeorology* **34**, 189-193, doi:10.1007/BF01048719 (1990).
19 Dransfield, M. The fungal air-spora at Samaru, Northern Nigeria. *Transactions of the British Mycological Society* **49**, 121-132, doi:http://dx.doi.org/10.1016/S0007-1536(66)80042-6 (1966).
20 Goncalves, F. L. *et al.* Indoor and outdoor atmospheric fungal spores in the Sao Paulo metropolitan area (Brazil): species and numeric concentrations. *Int J Biometeorol* **54**, 347-355, doi:10.1007/s00484-009-0284-6 (2010).
21 Bardei, F. *et al.* Incidence des spores fongiques de l'air de Tétouan (NW du Maroc) et influence des paramètres météorologiques. *Revue Française d'Allergologie* **53**, 576-584, doi:10.1016/j.reval.2013.05.004 (2013).
22 Kasprzyk, I. & Worek, M. Airborne fungal spores in urban and rural environments in Poland. *Aerobiologia* **22**, 169-176, doi:10.1007/s10453-006-9029-8 (2006).
23 Marks, R., Kruczalak, K., Jankowska, K. & Michalska, M. Bacteria and fungi in air over the Gulf of Gdansk and Baltic sea. *J Aerosol Sci* **32**, 237-250, doi:Doi 10.1016/S0021-8502(00)00064-1 (2001).
24 Oliveira, M., Ribeiro, H., Delgado, J. L. & Abreu, I. Seasonal and intradiurnal variation of allergenic fungal spores in urban and rural areas of the North of Portugal. *Aerobiologia* **25**, 85-98, doi:10.1007/s10453-009-9112-z (2009).
25 RodrÍGuez-Rajo, F. J., Iglesias, I. & Jato, V. Variation assessment of airborne Alternaria and Cladosporium spores at different bioclimatical conditions. *Mycological Research* **109**, 497-507, doi:10.1017/s0953756204001777 (2005).
26 Griffin, D. W., Garrison, V. H., Herman, J. R. & Shinn, E. A. African desert dust in the Caribbean atmosphere: Microbiology and public health. *Aerobiologia* **17**, 203-213, doi:10.1023/a:1011868218901 (2001).
27 Griffin, D. W. *et al.* Atmospheric microbiology in the northern Caribbean during African dust events. *Aerobiologia* **19**, 143-157, doi:10.1023/B:AERO.0000006530.32845.8d (2003).
28 Griffin, D. W. *et al.* Airborne desert dust and aeromicrobiology over the Turkish Mediterranean coastline. *Atmospheric Environment* **41**, 4050-4062, doi:10.1016/j.atmosenv.2007.01.023 (2007).
29 Lau, A. P. S., Lee, A. K. Y., Chan, C. K. & Fang, M. Ergosterol as a biomarker for the quantification of the fungal biomass in atmospheric aerosols. *Atmospheric Environment* **40**, 249-259, doi:10.1016/j.atmosenv.2005.09.048 (2006).

30 Katial, R. K., Zhang, Y., Jones, R. H. & Dyer, P. D. Atmospheric mold spore counts in relation to meteorological parameters. *International Journal of Biometeorology* **41**, 17-22 (1997).
31 Pady, S. M. Quantitative Studies of Fungus Spores in the Air. *Mycologia* **49**, 339-353, doi:Doi 10.2307/3755684 (1957).
32 Levetin, E. & Dorsey, K. Contribution of leaf surface fungi to the air spora. *Aerobiologia* **22**, 3-12, doi:10.1007/s10453-005-9012-9 (2006).
33 Mallo, A. C., Nitiu, D. S. & Gardella Sambeth, M. C. Airborne fungal spore content in the atmosphere of the city of La Plata, Argentina. *Aerobiologia* **27**, 77-84, doi:10.1007/s10453-010-9172-0 (2010).
34 Sabariego, S., Díaz De La Guardia, C. & Alba, F. The effect of meteorological factors on the daily variation of airborne fungal spores in Granada (southern Spain). *International Journal of Biometeorology* **44**, 1-5 (2000).
35 Şakiyan, N. & Inceoğlu, Ö. Atmospheric concentrations of Cladosporium Link and Alternaria Nées spores in Ankara and the effects of meteorological factors. *Turkish Journal of Botany* **27**, 77-81 (2003).
36 Hamilton, E. D. STUDIES ON THE AIR SPORA1. *Allergy* **13**, 143-175, doi:10.1111/j.1398-9995.1959.tb02761.x (1959).
37 Wu, Y.-H. *et al.* Characteristics, determinants, and spatial variations of ambient fungal levels in the subtropical Taipei metropolis. *Atmospheric Environment* **41**, 2500-2509, doi:10.1016/j.atmosenv.2006.11.035 (2007).
38 Wu, P. C., Tsai, J. C., Li, F. C., Lung, S. C. & Su, H. J. Increased levels of ambient fungal spores in Taiwan are associated with dust events from China. *Atmospheric Environment* **38**, 4879-4886, doi:10.1016/j.atmosenv.2004.05.039 (2004).
39 Kellogg, C. A. *et al.* Characterization of Aerosolized Bacteria and Fungi From Desert Dust Events in Mali, West Africa. *Aerobiologia* **20**, 99-110, doi:10.1023/B:AERO.0000032947.88335.bb (2004).
40 Choi, Y. W., Hyde, K. D. & Ho, W. H. Single spore isolation of fungi. *Fungal Diversity* **3**, 29-38 (1999).
41 Ho, H.-M. *et al.* Characteristics and determinants of ambient fungal spores in Hualien, Taiwan. *Atmospheric Environment* **39**, 5839-5850, doi:10.1016/j.atmosenv.2005.06.034 (2005).
42 Abu-Dieyeh, M. H., Barham, R., Abu-Elteen, K., Al-Rashidi, R. & Shaheen, I. Seasonal variation of fungal spore populations in the atmosphere of Zarqa area, Jordan. *Aerobiologia* **26**, 263-276, doi:10.1007/s10453-010-9162-2 (2010).
43 Pyrri, I. & Kapsanaki-Gotsi, E. Evaluation of the fungal aerosol in Athens, Greece, based on spore analysis. *Aerobiologia* **31**, 179-190, doi:10.1007/s10453-014-9355-1 (2015).
44 Rosas, I., Escamilla, B., Calderon, C. & Mosiño, P. The daily variations of airborne fungal spores in Mexico City. *Aerobiologia* **6**, 153, doi:10.1007/BF02539108 (1990).
45 Elvira-Rendueles, B. *et al.* Air-spore in Cartagena, Spain: Viable and non-viable sampling methods. *Ann Agr Env Med* **20**, 664-671 (2013).
46 Hasnain, S. M., Akhter, T. & Waqar, M. A. Airborne and allergenic fungal spores of the Karachi environment and their correlation with meteorological factors. *J Environ Monit* **14**, 1006-1013, doi:10.1039/c2em10545d (2012).

47 Herrero, A. D., Ruiz, S. S., Bustillo, M. G. & Morales, P. C. Study of airborne fungal spores in Madrid, Spain. *Aerobiologia* **22**, 133-140, doi:10.1007/s10453-006-9025-z (2006).

48 Winiwarter, W., Bauer, H., Caseiro, A. & Puxbaum, H. Quantifying emissions of primary biological aerosol particle mass in Europe. *Atmospheric Environment* **43**, 1403-1409, doi:10.1016/j.atmosenv.2008.01.037 (2009).

49 Abdel Hameed, A. A. *et al.* Study on some factors affecting survivability of airborne fungi. *Sci Total Environ* **414**, 696-700, doi:10.1016/j.scitotenv.2011.10.042 (2012).

50 Fang, Z. *et al.* Culturable airborne fungi in outdoor environments in Beijing, China. *Sci Total Environ* **350**, 47-58, doi:10.1016/j.scitotenv.2005.01.032 (2005).

51 Zhai, J. H. *et al.* Analysis and identification of atmospheric fungi in Beijing and Nanjing, China. *China Public Health* **16**, 1026–1027 (2000).

52 Hu, Q. X., Xu, X. Z., Chen, M. L., Tong, Y. Y. & Che, F. X. Study on atmospheric microbes in Shengyang III. The concentration and distribution of airborne fungal particles. *Microbiology* **21**, 281–285 (1994).

53 Jones, A. M. & Harrison, R. M. The effects of meteorological factors on atmospheric bioaerosol concentrations--a review. *Sci Total Environ* **326**, 151-180, doi:10.1016/j.scitotenv.2003.11.021 (2004).

54 Kalyoncu, F. Relationship between airborne fungal allergens and meteorological factors in Manisa City, Turkey. *Environ Monit Assess* **165**, 553-558, doi:10.1007/s10661-009-0966-x (2010).

55 Adhikari, A., Sen, M. M., Gupta-Bhattacharya, S. & Chanda, S. Airborne viable, non-viable, and allergenic fungi in a rural agricultural area of India: a 2-year study at five outdoor sampling stations. *Sci Total Environ* **326**, 123-141, doi:10.1016/j.scitotenv.2003.12.007 (2004).
