# Supplementary material for: Fungal spores as a source of sodium salt particles in the Amazon basin
Source: Nat Commun. 2018 Nov 19;9:4793. doi: 10.1038/s41467-018-07066-4 (PMC6242827; doi:10.1038/s41467-018-07066-4)
Supplement: Supplementary file 2 — Peer Review File [file 41467_2018_7066_MOESM2_ESM.pdf]

Reviewer #1 (Remarks to the Author):

This paper reports that fungal spores are major sources of particle sodium, with size distributions and properties that are similar to that from sea salt particles. This is a new finding that will be of broad interest and certainly justifies publication in Nature Communications. Given the importance of understanding the role of particles in cloud formation and particles, which impacts climate, this will definitely influence thinking in the field.

The paper is very well-written and documented, and this reviewer has essentially no substantiative comments or suggestions to make. A very (!!) minor suggestion is to add to the description of Supplementary Figure 9 the same description as for Figure 3b, since it is an unusual (but effective) way to present data.

Reviewer #2 (Remarks to the Author):

The major claim of the paper is that sodium salts present in atmospheric aerosols arise from fungal spores. Fungal spores represent a salt source that has previously not been reported.

This is a fascinating source. However, the manuscript's assessment of the importance of this finding is not supported by previous literature or clear explanations. Regarding previous literature, the abstract states, "In sharp contrast with the assumption that sodium-containing aerosols arise solely from marine sources,..." This statement is simply not true. There is a wealth of papers on non-marine sources of sodium salts in aerosols.

More problematically, while the observation of salts arises in fungal spores in interesting in its own right, the manuscript in its present form does not provide an adequate explanation or even compelling suggestions for how/when/under what circumstances or via what mechanism the salt in incorporated into some, but not all, spores. If an insightful discussion were included, I would rate this manuscript much more positively. As written, it is lacking.

A second finding is that spores which contain salt are more hygroscopic than those which do not. While I'm not aware of any previous results on precisely this topic, it is very well known that salts are

deliquescent materials whereas organic macromolecules and other organic compounds found in spores are not. So, this result is very obvious.

A minor point -

According to Figure 3, the Na fraction for fungal spores is estimated to be between 0.1 and 1.0 (i.e. nearly any value in the early hours, and later in the day transitions to an even wider spread in possible fractions. I may be missing something, but I don't find this result to be a significant finding.

in summary, the authors have one potentially interesting surprising field observation in the Amazon. However, additional consideration of how this may arise is needed before I could recommend publication.

Reviewer #3 (Remarks to the Author):

China et al. present new multi-instrument observations of the sodium content of fungal spores in the Amazon basin, and use those in combination with backward trajectories and earth system model simulations to suggest that the fungal spores are the main source of sodium containing particles in the wet season.

The major claims of the paper are as follows:

1. Fungal spores contribute significantly to particle-sodium in the Amazon, contributing at least 30% by number of sodium salt containing particles

2. Sodium-containing fungal spores show higher hygroscopic growth than sodium-free spores

3. Fungal spores account for ~62% of sodium mass during the wet season, according to model simulations

4. Therefore, the role of fungal spores in cloud formation and salt cycles in the Amazon deserves further consideration

The claims in the MS are significant and novel. The two closest papers known to me, one from (partly) the same research group, discuss that 1) K-rich bio-aerosols are seeds for SOA formation, but the paper does not mention Na-containing particles (Poehlker et al. 2010) and 2) that when spores rupture at high RH, fungal fragments that contain Na salts are released, but the paper does not quantify Na-content of the particles nor their contribution to the particle Na-budget over Amazonia (China et al. 2016).

The results are of interest to others in the community, since they give 1) new insight into Na-budget of the Amazon from a source that was not considered previously and 2) they add a new line of thought to the debate on sources of cloud-active particles in Amazon, which is a long-standing problem.

The results are of interest to the wider field, because this work adds a new aspect to the understanding of biogeochemical cycles in the Amazon rainforest, and how the forest itself sustains biogeochemical and hydrological recycling. Especially the role of fungal spores therein is one that has not received much attention so far, and this work is likely to start a further debate on this topic.

The MS is clearly structured and generally convincing, but I think it could profit from some further clarifications:

1. Since I am not familiar with the instruments that are used, I cannot judge the quality of the experimental work. However, I think the MS would benefit from a short explanation of the sampling procedure: are all coarse particles identified that were sampled? How is it determined that a particle is a fungal spore and not another kind of PBA (e.g. bacterium or pollen)? Is this done based on size, morphology or otherwise? A brief description could be added, for instance in L107.

2. The estimate of the contribution of spores to the Na-budget, depends on two factors which are very uncertain: local emissions of fungal spores and transport of sea salt aerosol into the Amazon basin. I think the fungal spores emissions are constrained reasonably well by the observations of their concentration, which the model seems to underestimate somewhat. However, if global emission estimates of sea salt span 2 orders of magnitude, how reliable is the estimate of sea salt aerosol transported into the Amazon basin, with no direct observations available for validation? Can the authors provide an estimate of the uncertainty in the sea salt contribution to the particle sodium budget? This could provide a bit more context to the number of 62% of total sodium mass that is now assigned to fungal spores.

Minor comments:

- Title: Since the main finding is that fungal spores contribute significantly to sodium salt particles, why not mention this in the title, for instance: ‘Fungal spores as source of sodium salt particles in the Amazon basin’

- L29-30: pls add that this is the case for the wet season

- In L52-53, you state that marine aerosol is considered the main source of coarse aerosol, whereas in L213 you state that fungal spores dominate the coarse mode. It would be good to clarify what is actually meant by dominate: mass, number? And to briefly include the evidence for the statement in L213.

- L107: how many particles were sampled and how was it ascertained that they are representative for the wet-season coarse aerosol? And how was a particle identified as a fungal spore? Was every collected particle in the sampling periods in Supp. Table 2 classified?

- L180: since the back trajectories and rain records are based on reanalysis data, I would not refer to them by 'observations' here, but rather by 'simulations'

- L190-191: could you clarify here where the percentages of 70 and 13% for sodium rich-spores and spore sodium mass, respectively, are based on?

- L208-209: how would nighttime emissions or a shallow nocturnal boundary layer explain that the Na fraction from spores drops only after noon, and not in the early morning at the onset of turbulence? Besides, the violin plots in figure 3 seem to show bimodal distributions for the Na fraction from fungal spores for each time of the day. What is the reason for that?

- L217: 'a major fraction to the sodium budget': please add here that this is for the wet season

- L533-534: which size distribution was assumed for the spores? Within the range of the model coarse mode (1-10 micron), the assumed size can make large differences for the efficiency of the wet and dry removal

- Supp. Fig. 9: It seems that the same violin plot is show twice: the plot looks very similar to that in Fig. 3 and the title says 'wet season' while the figure is for the dry season. Please include the right plot for the right season.

Technical corrections/suggestions:

- L32: pls remove 'we'

- L50: similar to concentration of marine air -> similar to concentrations of coarse mode particles in marine air

- L57: has -> have and is -> are

- L94: modeling studies -> model simulations

- L94: 'potential contributions of spores': contributions to what?

- Fig. 1, caption: representative is misspelled

- Supp. Table 1: Samples 3A and 3B have an end time prior to the start time

- Supp. Fig. 3: C/Na -> Cl/Na

We appreciate the reviewers' comments and suggestions; they provided positive and constructive feedback. The reviewers pointed out several important issues that we believe addressing will indeed strengthen the manuscript.

In the following discussion, the reviewers'comments are in black-colored font, our responses are in blue-colored font, and changes in the manuscript are noted by *italics blue-colored font.*

Reviewer #1 (Remarks to the Author):

This paper reports that fungal spores are major sources of particle sodium, with size distributions and properties that are similar to that from sea salt particles. This is a new finding that will be of broad interest and certainly justifies publication in Nature Communications. Given the importance of understanding the role of particles in cloud formation and particles, which impacts climate, this will definitely influence thinking in the field.
The paper is very well-written and documented, and this reviewer has essentially no substantiative comments or suggestions to make. A very (!!) minor suggestion is to add to the description of Supplementary Figure 9 the same description as for Figure 3b, since it is an unusual (but effective) way to present data.
We appreciate the positive response of the reviewer regarding the novelty and importance of the findings reported in the manuscript and for recommending the manuscript for publication. As suggested, we added more detailed description of Supplementary Figure 9 in the caption.
"*White dots represent the median, thick lines represent 25-75 percentile, and thin vertical lines represent the range of minimum-to-maximum values; violin plot represents a kernel density distribution of the sodium fraction values. Background shading indicates time of day, blue represents night [1800-0600] and yellow represents day [0600-1800].*"

Reviewer #2 (Remarks to the Author):

1. The major claim of the paper is that sodium salts present in atmospheric aerosols arise from fungal spores. Fungal spores represent a salt source that has previously not been reported. This is a fascinating source. However, the manuscript's assessment of the importance of this finding is not supported by previous literature or clear explanations. Regarding previous literature, the abstract states, "In sharp contrast with the assumption that sodium-containing aerosols arise solely from marine sources,..." This statement is simply not true. There is a wealth of papers on non-marine sources of sodium salts in aerosols.
We agree that fungal spores are a fascinating source of sodium salt particles in Amazonia. Sodium content in atmospheric aerosol is primarily attributed to sea water, especially the coarse mode particles. However, as the reviewer pointed out, there are also non-marine sources of sodium salts in atmospheric aerosols. For example, Ooki et al[1] observed the existence of anthropogenic sodium in fine mode aerosol particles in urban air. Ooki et al[1] suggested potassium as a tracer for anthropogenic sodium. Mamane[2] found presence of sodium and potassium in submicron particles from waste incinerators emissions. Another study conducted in

the metropolitan area of São Paulo by Bourotte et al.[3] found K/Na ratio of 1.4 and 0.9 for coarse and fine particles respectively and they attributed this particles to waste incineration and vehicles. Most of these studies show the presence of sodium in fine mode and suggest potassium as a tracer for anthropogenic sodium. Observations of our study are fundamentally different. First, our study focuses on coarse mode particles. Second, the potassium observed in our study mostly originates from biological particles[4], especially during the wet season when biomass burning is not a dominant source in the basin.

To clarify these details, we modified the sentence and the manuscript (as suggested by Reviewer 3) to reflect that our main finding is that fungal spores contribute to sodium salt particles in the Amazon basin.

"In contrast to the common assumption that sodium-containing aerosols are primarily arise from marine sources, our results suggest that locally-emitted fungal spores containing sodium contribute substantially to the number and mass of coarse particles containing Na and Cl (similar to sea salt)."

We adopted the title proposed by reviewer 3.

"Fungal spores as a source of sodium salt particles in the Amazon basin"

2. More problematically, while the observation of salts arises in fungal spores in interesting in its own right, the manuscript in its present form does not provide an adequate explanation or even compelling suggestions for how/when/under what circumstances or via what mechanism the salt in incorporated into some, but not all, spores. If an insightful discussion were included, I would rate this manuscript much more positively. As written, it is lacking.

In the original manuscript we provided basic information (lines: 167-172) regarding the nature for sodium content in fungal spores. To address the reviewers concerns, in the revised manuscript we provided more detailed discussion on this topic.

The following text was added in the revised manuscript (lines: 172-190).

"The presence of sodium is common in so called halophilic fungi which cannot grow without NaCl[5]. Active discharge, uptake, and efflux processes are likely responsible for the sodium content in the spores and sodium content may vary with different classes and genera of fungi. During active discharge, fungi forcibly eject spores into the atmosphere, together with osmotic fluid containing hexoses, mannitol, phosphate, sodium, and potassium[4,6]. Fungi require K^+ for electrical and osmotic equilibria of the cells and presence of K^+ in several fungal spores is well understood[7]. Previous studies suggested that K^+ can be partially replaced by Na^+ and Na^+ can enhance the growth of fungi[8] and plants[9] under K^+ deficiency conditions. The growth of fungi and uptake of Na^+ varies with their physiological conditions and uptake rates depend on the species and their genes[10]. For example, specific genes (e.g., acu1 and acu2) are responsible for high-affinity Na^+ uptake of Ustilago maydis. When fungi contain excess Na^+, they activate Na^+-efflux ATPase (Adenosinetriphosphatase), which acts as a key enzyme for the biological evolution of fungi[11]. We suggest that uptake and efflux of Na^+ varies with different classes and genera of fungal community. For example, previous studies in Amazonia shows several genera (e.g., Agaricus; Amanita; Aspergillus; Boletus; Cladonia; Mortierella; Puccinia; Lepsita; and Rhizopus) within one class of fungi (Lecanonomycetes)[12,13]. Furthermore, the transpiration of plants[14] and nutrient uptake[15] can also influence the sodium content of spores. Further studies

are needed to better comprehend the sodium content in fungal spores by linking chemical composition, molecular biology and diversity of fungal spores."

3. A second finding is that spores which contain salt are more hygroscopic than those which do not. While I'm not aware of any previous results on precisely this topic, it is very well known that salts are deliquescent materials whereas organic macromolecules and other organic compounds found in spores are not. So, this result is very obvious.
Indeed, it is expected that sodium-containing spores are more hygroscopic that those without sodium. Even if the growth factors are similar for sodium chloride particles and sodium containing spores, it is interesting to note that growth factors of NaCl and sodium-containing spores are different at relative humidity between 70 and 96%. Notably, variations in the hygroscopicity are associated with variations in the chemistry of these spores. We believe this finding is important and worth discussing in the manuscript.

A minor point –
4. According to Figure 3, the Na fraction for fungal spores is estimated to be between 0.1 and 1.0 (i.e. nearly any value in the early hours, and later in the day transitions to an even wider spread in possible fractions. I may be missing something, but I don't find this result to be a significant finding.
We believe Figure 3b provides important contextual information to help readers understand the temporal variability in the simulated fungal spore contribution to sodium, near the measurement site. Considering the high temporal variability of aerosol particle concentrations, including the simulated sea salt and fungal spores in this study (Supplementary Figure 11), it is not surprising that the relative contribution of fungal spores to total sodium mass spans a wide range of values. Figure 3b shows that from midnight to noon, the wet season median contribution of fungal spores to Na ranges from about 0.5 to 0.6. This implies that fungal spores are the dominant contributors of Na on the majority of days at these times. By contrast, from 3 pm to 9 pm, however, the median ranges from 0.15 to 0.3, and the dry season median fungal spore fraction ranges between only about 0.1 and 0.2 (Supplementary Figure 11). In other words, the model helps contextualize the limited number of observations, by identifying the circumstances under which the fungal spores are predicted to contribute the majority to simulated boundary-layer Na at the site: specifically, during the late night and early morning hours of the wet season.

5. In summary, the authors have one potentially interesting surprising field observation in the Amazon. However, additional consideration of how this may arise is needed before I could recommend publication.
We appreciate the comments and suggestions provided by the reviewer. As outlined in the response to the comments 2, in the revised manuscript we expanded the discussion substantially regarding the presence of sodium in fungal spores. In addition, the observation of sodium in fungal spores is on samples from a range of different days.

Reviewer #3 (Remarks to the Author):
China et al. present new multi-instrument observations of the sodium content of fungal spores in the Amazon basin, and use those in combination with backward trajectories and earth system model simulations to suggest that the fungal spores are the main source of sodium containing particles in the wet season.

The major claims of the paper are as follows:
1. Fungal spores contribute significantly to particle-sodium in the Amazon, contributing at least 30% by number of sodium salt containing particles
2. Sodium-containing fungal spores show higher hygroscopic growth than sodium-free spores
3. Fungal spores account for ~62% of sodium mass during the wet season, according to model simulations
4. Therefore, the role of fungal spores in cloud formation and salt cycles in the Amazon deserves further consideration
The claims in the MS are significant and novel. The two closest papers known to me, one from (partly) the same research group, discuss that 1) K-rich bio-aerosols are seeds for SOA formation, but the paper does not mention Na-containing particles (Poehlker et al. 2010) and 2) that when spores rupture at high RH, fungal fragments that contain Na salts are released, but the paper does not quantify Na-content of the particles nor their contribution to the particle Na-budget over Amazonia (China et al. 2016).

The results are of interest to others in the community, since they give 1) new insight into Na-budget of the Amazon from a source that was not considered previously and 2) they add a new line of thought to the debate on sources of cloud-active particles in Amazon, which is a long-standing problem.

The results are of interest to the wider field, because this work adds a new aspect to the understanding of biogeochemical cycles in the Amazon rainforest, and how the forest itself sustains biogeochemical and hydrological recycling. Especially the role of fungal spores therein is one that has not received much attention so far, and this work is likely to start a further debate on this topic.
We appreciate the reviewers' careful reading of our manuscript and providing us positive feedbacks.

The MS is clearly structured and generally convincing, but I think it could profit from some further clarifications:
1) Since I am not familiar with the instruments that are used, I cannot judge the quality of the experimental work. However, I think the MS would benefit from a short explanation of the sampling procedure: are all coarse particles identified that were sampled? How is it determined that a particle is a fungal spore and not another kind of PBA (e.g. bacterium or pollen)? Is this done based on size, morphology or otherwise? A brief description could be added, for instance in L107.

Initially, due to space constraints, we couldn't provide details on the methods used for identifying the fungal spores in the manuscript. Typically fungal spores are 1-6µm, while pollen

grains are larger in size (5−150 μm), bacteria are smaller (<1μm) and typically elongated in shape. As we selected only impactor stages 4 and 5 (aerodynamic diameter range: 1.0-3.2 μm) for this study, the fraction of bacteria and pollen is almost negligible. We also refer to our previous publication (China et al., 2016) where we described identification of fungal spores.

To address reviewer's comment, we moved portion of text from the methods section to the main manuscript and provided additional explanations (lines: 108-112)

"The fraction of fungal spores was quantified based on their unique characteristic morphologies (spherical, rod-like or spheroidal in shape), size (1-6 μm), and chemical composition (mostly carbonaceous and containing phosphorous) by electron microscopy imaging and X-ray microanalysis of over 3500 individual particles. Details of the fungal spore identification method are provided elsewhere[16]."

We added the following text in the method section (lines: 529-534):

"The computer-controlled CCSEM/EDX analysis automatically investigates the specified scanning area and detects particles in the specified fields of view. In this way, we can detect ~90% of the particles deposited on the grid. Particles collected on the edges (~5-10%) of the Cu mesh were excluded from the results based on their high Cu background signal. We analyzed ~80% of the particles that were collected onto TEM B-film grids placed on stages 4 and 5 of the impactor."

2. The estimate of the contribution of spores to the Na-budget, depends on two factors which are very uncertain: local emissions of fungal spores and transport of sea salt aerosol into the Amazon basin. I think the fungal spores emissions are constrained reasonably well by the observations of their concentration, which the model seems to underestimate somewhat. However, if global emission estimates of sea salt span 2 orders of magnitude, how reliable is the estimate of sea salt aerosol transported into the Amazon basin, with no direct observations available for validation? Can the authors provide an estimate of the uncertainty in the sea salt contribution to the particle sodium budget? This could provide a bit more context to the number of 62% of total sodium mass that is now assigned to fungal spores.

The reviewer is correct that the model emission estimates of the sea salt are uncertain.

To address this, we compared model estimates of atmospheric sea salt concentration with observed values at few coastal and remote island sites to investigate the fidelity of the model estimates of sodium from sea-salt. We have added a scatterplot comparing observed sodium mass concentration with model-simulated mass sodium mass concentration from sea-salt, together with a global map (Supplementary figure 9) of measurement sites.

We added the comparison of model-simulated and measured sodium mass (Supplementary figure 10) and added the following text.

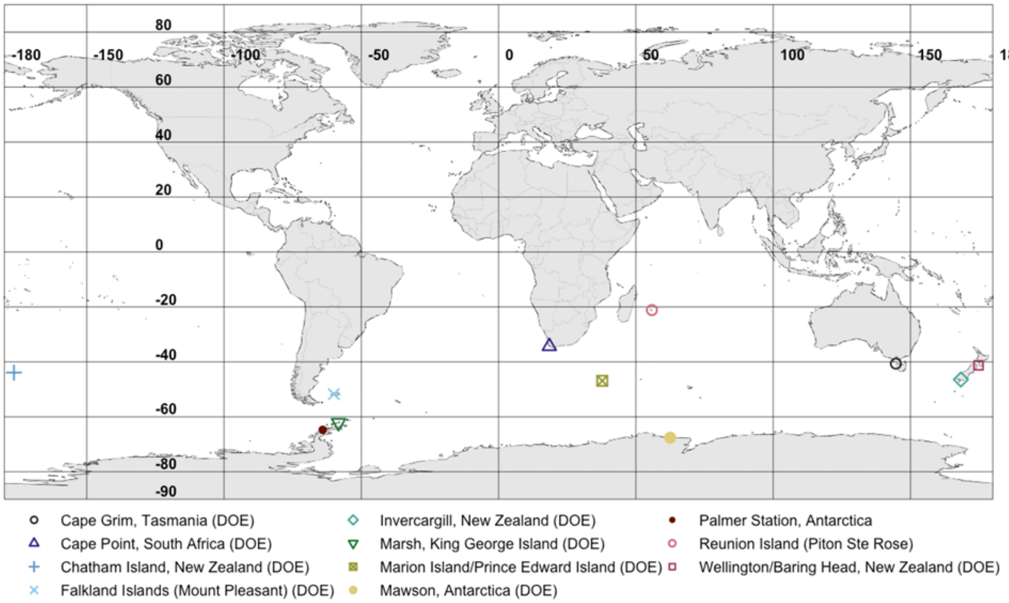

Supplementary Figure 8. Global map of sodium measurement sites, from the AEROCE/SEAREX. *Symbols represent locations where measurements of the atmospheric aerosol sodium mass concentration are available and are compared with model-estimated sodium mass concentration. Note that many sites in this network sampled aerosol only for certain wind directions (sector sampling); sector-sampled sites were excluded from this comparison to remove impacts of sampling bias.*

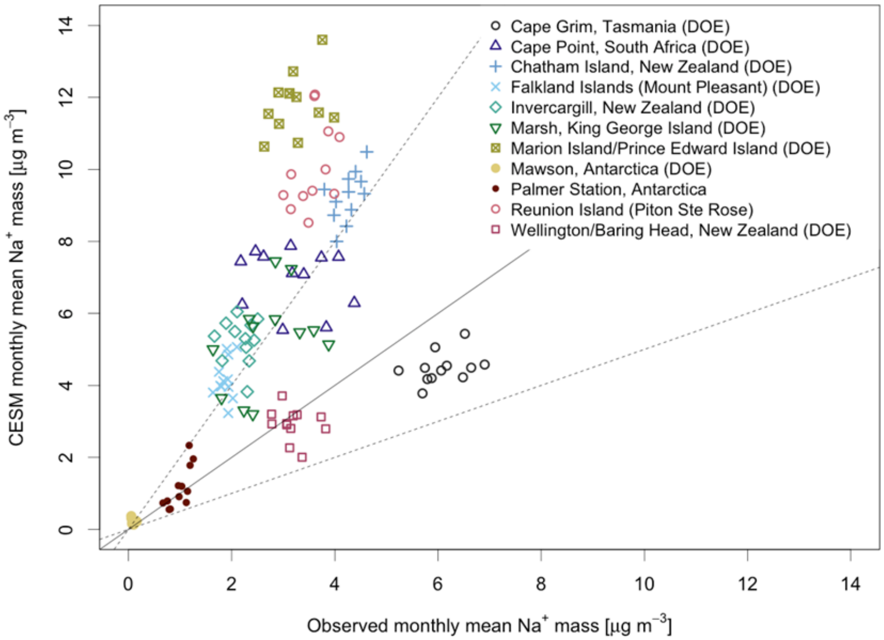

Supplementary Figure 9. Comparison of climatological monthly mean model-simulated and observed sodium mass from sea-salt. *Observations are multi-year climatological monthly means of all available years from the AEROCE/SEAREX observational dataset (http://aerocom.met.no/databenchmarks.html)[17-19], collected at sampling sites shown in Supplementary Figure 8. Note that to ensure a representative comparison, this analysis has*

excluded sites in the measurement network that sampled aerosol only for certain wind directions (sectored sampling). The solid line represents the 1:1 line, and the dashed lines represent an over-or under-estimate of a factor of two (2:1 and 1:2).

"Comparison between model-estimated and measured sodium mass concentrations from sea salt emissions from various geographical locations at the coastal and island sites (Supplementary Figure 9) are illustrated in Supplementary Figure 10. The model estimates of sodium mass concentrations are approximately a factor of two higher than observations at many of the measurement sites; the measurements used in this analysis were all collected at coastal and island locations in the Southern hemisphere, between 20 and 70 S. It is interesting to note that measurements at the remote sites (e.g., Antarctica) are in good agreement with the estimated mass."

"We calculate approximate upper and lower bounds for the fungal spore contribution to the total sodium mass by varying fungal spore mass concentrations by a factor of ten and sea salt mass concentrations by a factor of two. On average, fungal spores are estimated to contribute 69% (31-95%) of sodium during the wet season. However, the level of confidence in fungal spore estimates is arguably higher for the Amazon, since the emissions parameterization was originally developed based primarily on measurements from the Amazon and other tropical rainforests, and simulated fungal spore number concentrations agree with measurements at the Amazonia site within a factor of two. Assuming a factor of two uncertainty bounds for fungal spore concentrations, their estimated contribution to sodium mass would range between 51-84%."

We would also like to note that the sodium mass fraction in sea salt in the original manuscript was underestimated. The sodium mass has been corrected (30% sodium in sea-salt)[20] and all values recalculated. The revised model estimate of fungal spore contribution to the total sodium mass is 69% during wet season.

Minor comments:

1. Title: Since the main finding is that fungal spores contribute significantly to sodium salt particles, why not mention this in the title, for instance: 'Fungal spores as source of sodium salt particles in the Amazon basin'
Based upon the reviewer's suggestion the title is changed to
"Fungal spores as a source of sodium salt particles in the Amazon basin"

2. L29-30: pls add that this is the case for the wet season
We modified the sentence to clarify that the finding is valid for wet season.
"..we show that fungal spores emitted by the forest biosphere contribute to at least 30% (by number) of sodium salt particles observed in the central Amazon basin during the wet season."

3. In L52-53, you state that marine aerosol is considered the main source of coarse aerosol, whereas in L213 you state that fungal spores dominate the coarse mode. It would be good to

clarify what is actually meant by dominate: mass, number? And to briefly include the evidence for the statement in L213.

When we state that marine aerosol is considered the main source of coarse aerosol, we meant this was a common assumption, in contrast to our measurements. We meant that fungal spores dominate the coarse mode by number in below-canopy air, and at night, based on our microscopy observations. Fractions of fungal spores are provided in lines 113-116:

"During sampling, the number fraction of fungal spores was higher below the canopy (~60%) than above the canopy (~38%) and higher during night (~52%) than day (~37%)." Figure 1b demonstrate the number fraction of Na-rich particles.

We modified the sentence as follows (lines: 254-255).

"Overall, fungal spores dominated the number fraction of coarse particles in the Amazonia during nighttime and in below-canopy samples, and half of the coarse particles are sodium-rich."

4. L107: how many particles were sampled and how was it ascertained that they are representative for the wet-season coarse aerosol? And how was a particle identified as a fungal spore? Was every collected particle in the sampling periods in Supp. Table 2 classified?

As explained in the reply to the comment 4, we added the information of total number of particles in the main text (line: 111).

"The fraction of fungal spores was quantified .....by electron microscopy imaging and X-ray microanalysis of over 3500 individual particles."

Approximately 80% of the particles that were collected in the sampling period mentioned in the SI Table 2 were classified (see detailed reply in comment 1). We added the number of particles analyzed per sample in the supplementary table. We sampled particles for different durations (~2.5-8 hours) with varying duty cycles to get representative samples both during day- and night-time. We also performed back-trajectory analysis throughout the wet season and overall transport is similar to the transport pattern of airmass that was sampled during our sampling time. In addition, to better understand the representativeness of the analyzed particles with respect to the total particle population, we performed statistical analysis (applying binomial distribution)[21] to estimate the particle sample size of the particle population with given confidence interval (99%) and margin of error. We revised supplementary table 1 to reflect that samples particles are representative of the particle population with 99% confidence interval, and added the margin of error during each of the sampling periods.

As explained in the reply to the comment 1, we have provided additional text (lines: 108-112) in the main manuscript explaining the methods used for identification of fungal spores.

5. L180: since the back trajectories and rain records are based on reanalysis data, I would not refer to them by 'observations' here, but rather by 'simulations'

We changed the sentence to make it clear.

"Hence, these findings support the dominance of locally-emitted spores in the Amazon basin during this study."

6. L190-191: could you clarify here where the percentages of 70 and 13% for sodium rich-spores and spore sodium mass, respectively, are based on?

We added the following sentences in the revised manuscript to clarify our results (lines: 536-540)

"The percentage of sodium content in fungal spores was estimated using the known mass fraction of spores[22] and carbon/sodium wt% from X-ray microanalysis. Carbon content in spores ranges from 42-66%, with an average of 51%[23]. The average carbon/sodium wt% in fungal spores is 4:1. The mass of the spores is estimated from the size distribution of microscopy analysis and assuming a density of 1 gm cm^{-3})[22,24]."

Our estimation of 70% represents an upper bound. In the revised manuscript we provide a sensitivity analysis where we use different fractions (e.g., 30% and 50%) of sodium-containing fungal spores estimated from the samples. We add a modeled time series plot of sodium mass contributed by fungal spores assuming 30% and 50% sodium-rich spores in the sample (Supplementary Figure 13). We added few lines (242-245) to present this sensitivity analysis.

"We performed a sensitivity analysis by assuming either 30% or 50% of fungal spores to be sodium-rich. When applying these different assumptions to the model simulations, fungal spores account for 58% (21-90%) or 65% (27-93%) of the total simulated sodium mass during the wet season (Supplementary Fig. 13)."

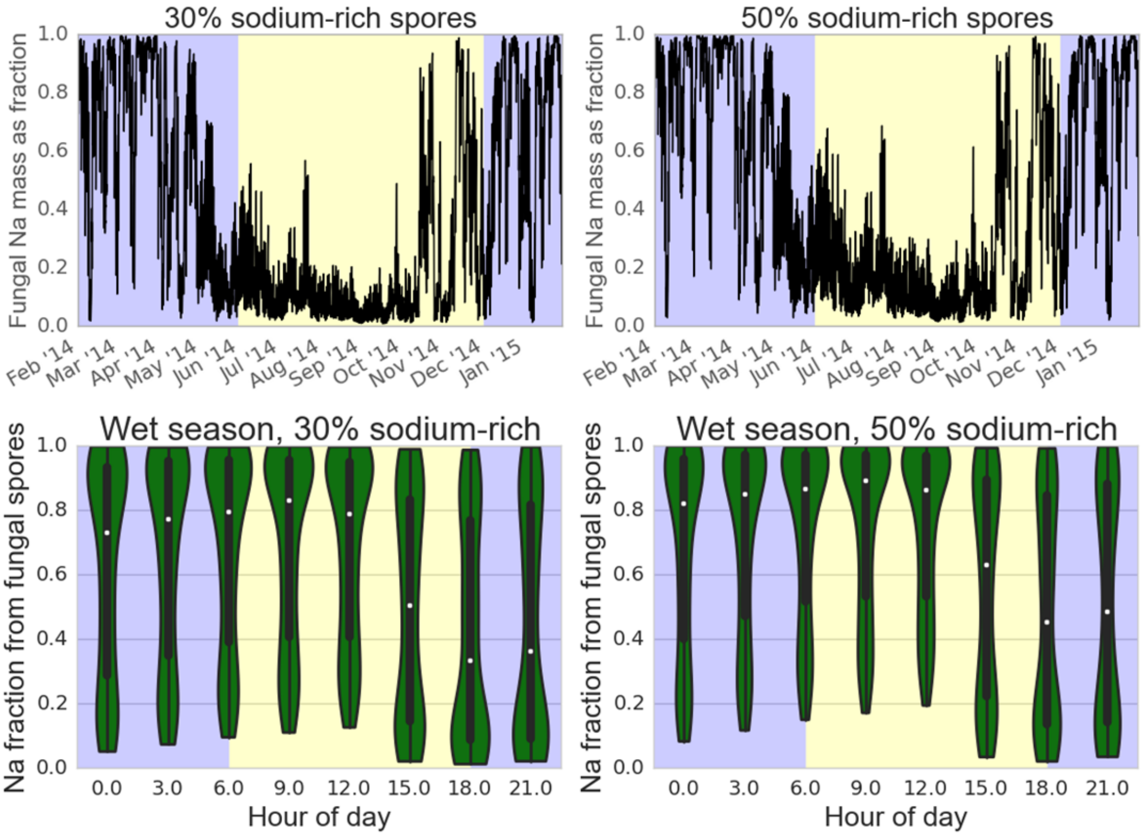

Supplementary Figure 9. Modeled time series of sodium mass contribution from fungal spores assuming different number fractions of sodium-rich spores. *Modeled time series (March 2014 – Feb 2015) of sodium mass percentage contributed by fungal spores assuming a) 30% and b) 50% of spores are sodium-rich.*

7. L208-209: how would nighttime emissions or a shallow nocturnal boundary layer explain that the Na fraction from spores drops only after noon, and not in the early morning at the onset of turbulence? Besides, the violin plots in figure 3 seem to show bimodal distributions for the Na fraction from fungal spores for each time of the day. What is the reason for that?

We suggest that there may be residual particles from the night time emissions that can contribute to the sodium fraction from fungal spores beyond the onset turbulence. It is also possible that high relative humidity throughout the day may be responsible for the higher fraction of fungal spores in the late morning. We added the following senescence in the manuscript.

"This may be the combined result of diurnal cycles in emissions, boundary-layer dynamics, and removal processes (e.g., precipitation)."

The bimodal distribution of the fractional contribution (as seen on the violin plots) is a reflection of the strong variability in simulated sea salt mass concentrations at the site. As a consequence of this variability, most of the time, simulated values of sea salt sodium mass are either much greater or much less than fungal spore sodium mass. Since fractional contributions are presented here, the values cluster in the upper and lower portions of the range 0-1.

8. L217: 'a major fraction to the sodium budget': please add here that this is for the wet season

We clarified that the statement is for the wet season.

"Remarkably, our experimental and modeling results demonstrate that fungal spores emitted from the rainforest biosphere contribute a major fraction to the sodium budget during wet season…"

9. L533-534: which size distribution was assumed for the spores? Within the range of the model coarse mode (1-10 micron), the assumed size can make large differences for the efficiency of the wet and dry removal

We added the following lines to clarify the size distribution used in the model.

"Upon emission both fungal spore mass and coarse mode number are increased, with the mass-to-number ratio for emissions determined by assuming that fungal spores have 4 µm diameter upon emission. The model's coarse mode particle number concentration also includes number contributions from other aerosol species, which are treated as internally mixed within the coarse mode, and the particle diameter is calculated dynamically by the aerosol microphysics routines from the total coarse mode aerosol mass and number, and a fixed geometric width."

10. Supp. Fig. 9: It seems that the same violin plot is show twice: the plot looks very similar to that in Fig. 3 and the title says 'wet season' while the figure is for the dry season. Please include the right plot for the right season.

By mistake we placed the wet season plot. We incorporated the correct dry season plot in the revised manuscript.

11. Technical corrections/suggestions:- L32: pls remove 'we'

Done.

12. L50: similar to concentration of marine air -> similar to concentrations of coarse mode particles in marine air
Corrected.

13. L57: has -> have and is -> are
Corrected.

14. L94: modeling studies -> model simulations
Done.

15. L94: 'potential contributions of spores': contributions to what?

16. Fig. 1, caption: representative is misspelled
Corrected.

17. Supp. Table 1: Samples 3A and 3B have an end time prior to the start time
Corrected

18. Supp. Fig. 3: C/Na -> Cl/Na
Corrected.

References

1 Ooki, A., Uematsu, M., Miura, K. & Nakae, S. Sources of sodium in atmospheric fine particles. *Atmospheric Environment* **36**, 4367-4374, doi:https://doi.org/10.1016/S1352-2310(02)00341-2 (2002).

2 Mamane, Y. Estimate of municipal refuse incinerator contribution to philadelphia aerosol-I. Source analysis. *Atmospheric Environment (1967)* **22**, 2411-2418, doi:https://doi.org/10.1016/0004-6981(88)90473-8 (1988).

3 Bourotte, C. *et al.* Association between ionic composition of fine and coarse aerosol soluble fraction and peak expiratory flow of asthmatic patients in São Paulo city (Brazil). *Atmospheric Environment* **41**, 2036-2048, doi:https://doi.org/10.1016/j.atmosenv.2006.11.004 (2007).

4 Pöhlker, C. *et al.* Biogenic potassium salt particles as seeds for secondary organic aerosol in the Amazon. *Science* **337**, 1075-1078 (2012).

5 Plemenitaš, A. *et al.* Adaptation to high salt concentrations in halotolerant/halophilic fungi: a molecular perspective. *Frontiers in Microbiology* **5**, doi:10.3389/fmicb.2014.00199 (2014).

6 Webster, J. *et al.* Mannitol and hexoses are components of Buller's drop. *Mycological Research* **99**, 833-838, doi:http://dx.doi.org/10.1016/S0953-7562(09)80737-5 (1995).

7 Rodríguez-Navarro, A. Potassium transport in fungi and plants. *Biochimica et Biophysica Acta (BBA) - Reviews on Biomembranes* **1469**, 1-30, doi:https://doi.org/10.1016/S0304-4157(99)00013-1 (2000).

8 Camacho, M., Ramos, J. & Rodríguez-Navarro, A. Potassium requirements ofSaccharomyces cerevisiae. *Current Microbiology* **6**, 295-299, doi:10.1007/bf01566880 (1981).
9 Subbarao, G. V., Wheeler, R. M., Stutte, G. W. & Levine, L. H. How far can sodium substitute for potassium in red beet? *Journal of Plant Nutrition* **22**, 1745-1761, doi:10.1080/01904169909365751 (1999).
10 Benito, B., Garciadeblás, B., Schreier, P. & Rodríguez-Navarro, A. Novel P-type ATPases mediate high-affinity potassium or sodium uptake in fungi. *Eukaryotic Cell* **3**, 359-368 (2004).
11 Benito, B., Garciadeblás, B. & Rodríguez-Navarro, A. Potassium- or sodium-efflux ATPase, a key enzyme in the evolution of fungi. *Microbiology* **148**, 933-941, doi:doi:10.1099/00221287-148-4-933 (2002).
12 Womack, A. M. *et al.* Characterization of active and total fungal communities in the atmosphere over the Amazon rainforest. *Biogeosciences* **12**, 6337-6349, doi:10.5194/bg-12-6337-2015 (2015).
13 Fröhlich-Nowoisky, J. *et al.* Biogeography in the air: fungal diversity over land and oceans. *Biogeosciences* **9**, 1125-1136, doi:10.5194/bg-9-1125-2012 (2012).
14 Nemeryuk, G. Migration of salts into the atmosphere during transpiration. *Soviet Plant Physiol* (1970).
15 Lawson, I. T., Jones, T. D., Kelly, T. J., Coronado, E. N. H. & Roucoux, K. H. The geochemistry of Amazonian peats. *Wetlands* **34**, 905-915 (2014).
16 China, S. *et al.* Rupturing of Biological Spores As a Source of Secondary Particles in Amazonia. *Environmental Science & Technology* **50**, 12179-12186, doi:10.1021/acs.est.6b02896 (2016).
17 L., S. D. *et al.* Marine biogenic and anthropogenic contributions to non-sea-salt sulfate in the marine boundary layer over the North Atlantic Ocean. *Journal of Geophysical Research: Atmospheres* **107**, AAC 3-1-AAC 3-21, doi:doi:10.1029/2001JD000970 (2002).
18 Gong, S. *et al.* Modeling sea-salt aerosols in the atmosphere: 2. Atmospheric concentrations and fluxes. *Journal of Geophysical Research: Atmospheres* **102**, 3819-3830 (1997).
19 Li-Jones, X. & Prospero, J. Variations in the size distribution of non-sea-salt sulfate aerosol in the marine boundary layer at Barbados: Impact of African dust. *Journal of Geophysical Research: Atmospheres* **103**, 16073-16084 (1998).
20 Pilson, M. An Introduction to the Chemistry of the Sea Prentice-Hall. *Inc. Upper Saddle River, NJ. 431p* (1998).
21 Levy, P. S. & Lemeshow, S. *Sampling of populations: methods and applications*. (John Wiley & Sons, 2013).
22 Elbert, W., Taylor, P. E., Andreae, M. O. & Pöschl, U. Contribution of fungi to primary biogenic aerosols in the atmosphere: wet and dry discharged spores, carbohydrates, and inorganic ions. *Atmos. Chem. Phys.* **7**, 4569-4588, doi:10.5194/acp-7-4569-2007 (2007).
23 Bauer, H. *et al.* Determination of the carbon content of airborne fungal spores. *Analytical Chemistry* **74**, 91-95 (2002).
24 Huffman, J. A. *et al.* Size distributions and temporal variations of biological aerosol particles in the Amazon rainforest characterized by microscopy and real-time UV-APS

fluorescence techniques during AMAZE-08. *Atmos. Chem. Phys.* **12**, 11997-12019, doi:10.5194/acp-12-11997-2012 (2012).

Reviewer #2 (Remarks to the Author):

As I pointed out in my initial review, there are many non-marine sources of sodium salts, which the manuscript failed to discuss. It is frustrating to me that the authors acknowledge this fact in their response and yet refuse to improve the text accordingly. A number of relevant references are missing. In fact, the authors discuss a number of these in their rebuttal but don't include them in the revised text. I find the manuscript seriously lacking in this regard. It is a serious problem in the discussion and introduction. It would seem that the authors are concerned that once the proper references are cited, the novelty of their manuscript will fall beneath the high standards of Nature. Additionally, failure to acknowledge and discuss Na sources is a weakness in the estimates of how much salt in the Amazon comes from marine sources. I appreciate the effort in revising and adding the modeled estimates of sources. However, that section is very simplistic and I question the merit of conclusions drawn from it.

Also, the authors state in their response that other Na sources in aerosol are "completely

different" because they are small sized aerosols, whereas fungal spores are in the coarse mode. This statement is contradictory to the discussion in the manuscript text that describes the bursting of fungal spores and generation of smaller aerosol as a major reason that fungal spores have climatic relevance.

What is known in the literature about the concentration of NaCl in spores? I suspect that a lot more work has been conducted that is not cited here.

The experimental details are sparse. My concern that the highly variable present of salt may simply be a function of operator error (and the occasional contamination of some samples through contain with human skin or contaminated (or re-used) laboratory gloves. I note that another reviewer requested additional details, and the authors chose not to include them, but rather to refer readers to a previous manuscript. I appreciate the limitation of space, but this does not help to eleviate my concern that the experimental results are artifacts rather than new findings.

Reviewer #3 (Remarks to the Author):

The authors have appropriately addressed the comments of me and the other two reviewers. I only have one (very minor) comment on the revised version of the MS: in the main text (lines 236-256), the authors discuss the uncertainty in the 69% contribution of fungal spores to sodium mass, due to assumptions on spore sodium content and sea salt contribution. I would like to see this uncertainty reflected in the number that is given in the abstract, so could you please indicate the uncertainty range there (line 36)?

My recommendation is therefore to publish the paper after this minor revision.

We thank the reviewers' for their comments and suggestions. In the following discussion, the reviewers' comments are in black-colored font, our responses are in blue-colored font, and changes in the manuscript are noted by *italics blue-colored font.*

Reviewer #2 (Remarks to the Author):

As I pointed out in my initial review, there are many non-marine sources of sodium salts, which the manuscript failed to discuss. It is frustrating to me that the authors acknowledge this fact in their response and yet refuse to improve the text accordingly. A number of relevant references are missing. In fact, the authors discuss a number of these in their rebuttal but don't include them in the revised text. I find the manuscript seriously lacking in this regard. It is a serious problem in the discussion and introduction. It would seem that the authors are concerned that once the proper references are cited, the novelty of their manuscript will fall beneath the high standards of Nature. Additionally, failure to acknowledge and discuss Na sources is a weakness in the estimates of how much salt in the Amazon comes from marine sources. I appreciate the effort in revising and adding the modeled estimates of sources. However, that section is very simplistic and I question the merit of conclusions drawn from it. Also, the authors state in their response that other Na sources in aerosol are "completely different" because they are small sized aerosols, whereas fungal spores are in the coarse mode. This statement is contradictory to the discussion in the manuscript text that describes the bursting of fungal spores and generation of smaller aerosol as a major reason that fungal spores have climatic relevance. What is known in the literature about the concentration of NaCl in spores? I suspect that a lot more work has been conducted that is not cited here.

In response to the reviewer's request, we added relevant text in the introduction as noted below (lines: 58-68). Regarding the bursting of fungal spores and generation of smaller particles, we suggest that total contribution of sodium budget from fungal spores can be even lager because we did not take into account the smaller particles in this study. This study focuses solely on the coarse mode particles and investigates samples from stages 4 and 5 (size range: 1.0-3.2 µm) where the relative abundance of biological particles is high.

"*This study shows locally emitted fungal spores of coarse super-micrometer size contribute considerably to sodium salt particles in the Amazon basin during wet season. While previous studies reported non-marine sources of sodium salts in fine particles, sodium-containing coarse particles have been attributed almost exclusively to sea-salt. Specifically, Ooki et al*[1] *observed the existence of anthropogenic sodium in fine mode (<1.1 µm) aerosol particles in urban air. Ooki et al*[1] *suggested potassium as a tracer for anthropogenic sodium in submicron size (0.43µm) particles. Mamane*[2] *found presence of sodium and potassium in <1 µm particles from waste incinerators emissions. Another study conducted in the metropolitan area of São Paulo found K/Na ratio of 1.4 and 0.9 for coarse (>2.5 µm) and fine (<2.5µm) particles respectively and they attributed this particles to waste incineration and vehicles*[3]."

We added the following text in the discussion (lines: 268-273)

"Previous studies in different atmospheric environment show the presence of sodium in fine mode and suggest potassium as a tracer for anthropogenic sodium[1-5]. Observations of our study are fundamentally different. First, our study focuses on the coarse mode particles. Second, the potassium observed in our study mostly originates from biological particles[6] confirmed by their unique and distinguishable morphology. Those particles were especially abundant during the wet season when biomass burning is not a dominant source of particles in the basin."

The experimental details are sparse. My concern that the highly variable present of salt may simply be a function of operator error (and the occasional contamination of some samples through contain with human skin or contaminated (or re-used) laboratory gloves. I note that another reviewer requested additional details, and the authors chose not to include them, but rather to refer readers to a previous manuscript. I appreciate the limitation of space, but this does not help to eleviate my concern that the experimental results are artifacts rather than new findings.

As we discussed in our revised manuscript, we suggest that variable present of sodium content may depend on different classes and genera of fungi. Sample collection and experiments were conducted with cautions. In our previous revised version as requested by the reviewer 3, we provided details of the method (please see the notes of the reviewer 3 who found our arguments convincing). To elaborate more on this note, we added the following text in the method section of the revised manuscript.

"All the experiments were performed with diligently. Samples were handled with caution. For examples, tweezers were cleaned with solvents prior to handling the samples; new gloves were used for handling of samples after each experiments."

<u>Reviewer #3 (Remarks to the Author):</u>

The authors have appropriately addressed the comments of me and the other two reviewers. I only have one (very minor) comment on the revised version of the MS: in the main text (lines 236-256), the authors discuss the uncertainty in the 69% contribution of fungal spores to sodium mass, due to assumptions on spore sodium content and sea salt contribution. I would like to see this uncertainty reflected in the number that is given in the abstract, so could you please indicate the uncertainty range there (line 36)?

My recommendation is therefore to publish the paper after this minor revision.

As suggested, we provided the uncertainty range in the abstract as well.

"Modeling results suggest that fungal spores account for ~69% (31-95%) of the total sodium mass during the wet season and that their fractional contribution increases during nighttime."

References

1 Ooki, A., Uematsu, M., Miura, K. & Nakae, S. Sources of sodium in atmospheric fine particles. *Atmospheric Environment* **36**, 4367-4374, doi:https://doi.org/10.1016/S1352-2310(02)00341-2 (2002).

2 Mamane, Y. Estimate of municipal refuse incinerator contribution to philadelphia aerosol-I. Source analysis. *Atmospheric Environment (1967)* **22**, 2411-2418, doi:https://doi.org/10.1016/0004-6981(88)90473-8 (1988).

3 Bourotte, C. *et al.* Association between ionic composition of fine and coarse aerosol soluble fraction and peak expiratory flow of asthmatic patients in São Paulo city (Brazil). *Atmospheric Environment* **41**, 2036-2048, doi:https://doi.org/10.1016/j.atmosenv.2006.11.004 (2007).

4 Kitto, M. E. Trace-Element Patterns in Fuel Oils and Gasolines for Use in Source Apportionment. *Air & Waste* **43**, 1381-1388, doi:10.1080/1073161X.1993.10467213 (1993).

5 Mizohata, A. & Mamuro, T. Chemical element balances and identification of air pollution sources in Sakai, Osaka (I). *Journal of Japan Society of Air Pollution* **15**, 198-206 (1980).

6 Pöhlker, C. *et al.* Biogenic potassium salt particles as seeds for secondary organic aerosol in the Amazon. *Science* **337**, 1075-1078 (2012).

Fungal spores as a source of sodium salt particles in the Amazon basin

Swarup China[1], Susannah Burrows[2], Bingbing Wang[3] ,Tristan H. Harder[4,5,‖], Johannes Weis[4,5], Meryem Tanarhte[6], Luciana V. Rizzo[7], Joel Brito[8], Glauber G. Cirino[9], Po-Lun Ma[2], John Cliff[1], Paulo Artaxo[10], Mary K. Gilles[4], Alexander Laskin[11,*]

[1]*William R. Wiley Environmental Molecular Sciences Laboratory, Pacific Northwest National Laboratory, Richland, WA 99354, USA*

[2]*Atmospheric Science and Global Change Division, Pacific Northwest National Laboratory, Richland, WA 99354, USA*

[3]*State Key Laboratory of Marine Environmental Science, College of Ocean and Earth Science, Xiamen University, Xiamen 361102, China*

[4]*Chemical Sciences Division, Lawrence Berkeley National Laboratory, Berkeley, CA 94720, USA*

[5]*Department of Chemistry, University of California, Berkeley, California 94720, USA.*

[6]*Max Planck Institute for Chemistry, Mainz, Germany*

[7]*Universidade Federal de Sao Paulo, Diadema, SP, Brazil*

[8]*Laboratory for Meteorological Physics, University Clermont Auvergne, Clermont-Ferrand, France*

[9]*Geosciences Institute, Federal University of Para, Belem, 66075-110, Brazil*

[10] *Institute of Physics, University of São Paulo, Rua do Matão 1371, CEP 05508-090, São Paulo, S.P.,Brazil*

[11]*Department of Chemistry, Purdue University, West Lafayette, IN 47907, USA*

[‖]*present address: Physikalisches Institut, Universität Würzburg, Am Hubland, 97074 Würzburg, Germany*

Salt containing aerosols play important roles in atmospheric chemistry and cloud formation, hence understanding and observationally constraining their sources and processes are vital. In the Amazon basin, particles containing mixed sodium salts are routinely observed and attributed to marine aerosols transported from the Atlantic Ocean. Here, using detailed chemical imaging analysis, we show that fungal spores emitted by the forest biosphere (Primary Biogenic Aerosol Particles) contribute to at least 30% (by number) of sodium salt particles observed in the central Amazon basin during the wet season. Hydration experiments, using environmental microreactors complemented with micro-spectroscopy analysis, show that sodium-containing fungal spores have higher hygroscopic growth than sodium-free spores and their sodium content determines their growth factors. Modeling results suggest that fungal spores account for ~69% (31-95%) of the total sodium mass during the wet season and that their fractional contribution increases during nighttime. In contrast to the common assumption that sodium-containing aerosols are primarily arise from marine sources, our results suggest that locally-emitted fungal spores containing sodium contribute substantially to the number and mass of coarse particles containing Na and Cl (similar to sea salt). Hence, their role in cloud formation and contribution to salt cycles and the terrestrial ecosystem in the Amazon basin warrant further consideration.

The Amazon rainforest is often termed the “green ocean” because its relatively pristine atmospheric conditions resemble those in remote marine regions interns of the low particle count and cloud characteristics[1-3]. Specifically, the size distribution and concentration of coarse mode particles in Amazonia are similar to concentrations of coarse mode particles in marine air above the ocean under calm wind conditions of 1-10 m s^{-1} (Supplementary Fig. 1). During the wet season in the Amazon basin, marine aerosol from the Atlantic Ocean is considered the dominant source of coarse mode particles[1,4]. Previous studies assumed the chemical composition of coarse mode aerosols was strongly impacted by long-range transport of soil dust and marine aerosol advected into the central Amazon by large-scale tropospheric circulation that adds to the coarse mode primary biological aerosol particles[5]. However, local sources of sodium salt-containing coarse particles and their potential impact have not been considered and are the focus of this study. This study shows locally emitted fungal spores of coarse super-micrometer size contribute considerably to sodium salt particles in the Amazon basin during wet season. While previous studies reported non-marine sources of sodium salts in fine particles, sodium-containing coarse particles have been attributed almost exclusively to sea-salt. Specifically, Ooki et al[6] observed the existence of anthropogenic sodium in fine mode (<1.1 μm) aerosol particles in urban air. Ooki et al[6] suggested potassium as a tracer for anthropogenic sodium in submicron size (0.43μm) particles. Mamane[7] found presence of sodium and potassium in <1 μm particles from waste incinerators emissions. Another study conducted in the metropolitan area of São Paulo found K/Na ratio of 1.4 and 0.9 for coarse (>2.5 μm) and fine (<2.5μm) particles respectively and they attributed this particles to waste incineration and vehicles[8].

Primary biological aerosol particles (PBAP), i.e. particles emitted directly from the biosphere, include fungal spores, pollen, bacteria, algae, protozoa, and fragments of plants and living or dead organisms. Total global emissions of fungal spores are highly uncertain[9-11], estimates vary from 8 Tg per yr^{-1} to 186 Tg yr^{-1}, to as large as 1000 Tg yr^{-1}. Fungal species actively discharge their spores via liquid jets into the air[12,13], a process occurring preferentially under humid atmospheric conditions[13]. Fungal spores and fragments are the most abundant classes of biological particles in the Amazon basin. They contribute up to 25% during daytime (and 45% during nighttime) to the total number of coarse mode particles (1 to 10 µm diameter)[1].

Biological particles, as well as fluorescent particles (inferred to be biological) have been detected in the free troposphere[14,15]. Their chemical compositions are highly variable and, due to the inherent challenges involved in analytically distinguishing between biological and other carbonaceous particles, their exact origins remain insufficiently characterized[1,11]. In Amazonia, concentrations of fluorescent biological particles during the wet season of $\sim 7.3\times10^{-4}$ m^{-3} are reported, with the highest concentrations occurring during nighttime[16].

Once in the troposphere, biological particles can act as cloud condensation nuclei and ice nuclei, thus impacting warm and cold cloud formation and evolution[11,17]. During their airborne lifetime, Amazonian particles typically experience several cycles of cloud processing[1], which alters their physio-chemical properties and further thwarts their identification. In particular, biological spores rupture at high humidity due to osmotic pressure and subsequently release submicron-sized fragments[18,19]. These fragments contain inorganic salts with the same elements (e.g., Na, Mg, K, Cl) as sea salt. While fragmentation reduces the fraction of original supermicron-

sized biological particles, it increases their contribution to the number concentration of submicron particles. Hence, fragmentation due to atmospheric processing further complicates estimation of their number and size distributions in the accumulation mode[18].

Samples of airborne biological particles were collected during the beginning of the wet season (January and February, 2015) at the ZF2 tower, a pristine rainforest site in Central Amazonia located 40 km North of Manaus[4]. We applied multimodal chemical imaging techniques, such as scanning electron microscopy (SEM) coupled with energy dispersive X-ray (EDX) microanalysis, scanning transmission X-ray microscopy with near-edge X-ray absorption fine structure analysis (STXM/NEXAFS), and nano secondary ion mass spectrometry (NanoSIMS) to analyze size and composition of the biological particles. In situ exposure to water vapor in environmental SEM and STXM studies examined their hygroscopic growth. Finally, model simulations examined the potential contributions of spores in the Amazon basin atmosphere.

Results

Chemical imaging and micro-spectroscopy analysis of fungal spores. SEM images of representative coarse mode particles collected in the Amazon basin are illustrated in Fig 1a. X-ray microanalysis using computer-controlled (CC) SEM/EDX reveals that almost half (~48%) of the coarse mode particles (1.0-3.2 µm aerodynamic diameter) are Na-rich (Fig. 1b). Dust (containing Al, Si, Fe), or internally mixed biological-dust particles (C, N, P, Na, K, Al, Si, and Fe) are second most abundant. Coarse mode sulfate and purely carbonaceous particles are 5% and 3%, respectively. Overall, no considerable differences in the size distributions of different particle classes were observed during night or day or within, above, or below the canopy (Figs. 1c-f). X-

ray microanalysis indicates that the spores are comprised primarily of C, N, and O (carbonaceous in nature) with substantial amounts of other elements e.g., Na, Mg, P, S, Cl, and K (Figs. 2a-c), consistent with previous reports[18,20,21].

The fraction of fungal spores was quantified based on their unique characteristic morphologies (spherical, rod-like or spheroidal in shape), size (1-6 µm), and chemical composition (mostly carbonaceous and containing phosphorous) by electron microscopy imaging and X-ray microanalysis of over 3500 individual particles. Details of the fungal spore identification method are provided elsewhere[18]. The particle-type classification scheme is summarized in Supplementary Fig. 2. During sampling, the number fraction of biological particles was higher below the canopy (60%±2%) than above the canopy (38%±2%) and higher during night (52%±2%) than day (37%±2%). The coarse mode mass fractions of phosphorous and potassium, primarily derived from biological particles[13,22], were larger below than above the canopy and larger during night than day[23]. The higher fraction of biological particles below the canopy is consistent with a local source, and with observations from other tropical rainforests such as Borneo, Malaysia[24]. The increased nighttime number fraction of spores could result from enhanced nighttime active wet discharge of ascospores and basidiospores associated with elevated humidity[13]. Our observation of higher fractions of biological particles during nighttime below the canopy is consistent with previous studies in the Amazon rainforest[16,23,25].

Chemical imaging of the biological particles using X-ray absorption micro-spectroscopy confirms the presence of sodium. Figs. 2d-f show representative STXM images of a biological particle at the Na pre edge (1070 eV) and peak (1079 eV) as well as optical density map. The

sodium atomic weight fraction is size dependent; smaller particles contain a higher fraction of sodium than larger particles (Supplementary Fig. 3). The frequent occurrence of substantial sodium and chlorine masses in biological spores is remarkable. Since biological spores have never been considered as sources of sodium and chlorine, common analytical methods could mistakenly assign them as transported sea salt.

NanoSIMS analysis of fungal spores confirms the presence of sodium-containing particles (Figs. 2g-i) and provides spatial distributions of sodium and other elements within individual particles[26]. For example, the particle in Fig. 2g shows a stronger sodium signal around the particle boundary. That in Fig. 2h displays complex morphology and heterogeneity in its sodium spatial distribution, while the distribution of sodium ions in the elongated fungal spore in Fig. 2i appears homogeneous. These images suggest that sodium distributions within individual fungal spores is diverse, presumably depends upon spore type, and may be influenced by the particle's origin and aging history.

X-ray microanalysis indicates that the average percentage of total spores containing sodium (minimum detection of 3 wt %) is higher above the canopy (daytime: 60%±6%; nighttime: 39%±3%) than below the canopy (daytime: 46%±4%; nighttime: 22%±2%). These differences may partially be influenced by changes in physio-chemical properties of spores that transform during atmospheric processing. For example, when exposed to high humidity conditions, spores rupture and release submicrometer-to-micrometer size fragments. A major fraction (~40-60%) of these fragments contains Na and Cl, and appears morphologically similar to dry sea salt particles

(Supplementary Fig. 4). Subsequently, when exposed to high humidity these hygroscopic fragments grow and become supermicron[18].

Hygroscopicity of fungal spores. We investigated the hygroscopicity of spores using microreactors in environmental SEM[18] and in-situ STXM[27]. At 94% relative humidity (*RH)* sodium-containing fungal spores had area growth factors of 2.4 versus 1.1 if they were sodium-free (Supplementary Figs. 5 and 6). Similarly, mass growth factors of 3.6 and 1.5 were determined for sodium-containing and sodium-free fungal spores, respectively, at 96% *RH*. Remarkably, under high humidity conditions (*RH*~94%) the growth factors of sodium-containing biological particles and NaCl particles are strikingly similar. Using standard analytical methods or inspecting their morphology[18], these processed particles would not readily be recognized as spores. Due to the wide range of sources relevant to biological particle emissions which is further complicated by fragmentation during atmospheric processing, detecting and apportioning aged spores is a daunting task. Hence, microscopy methods underestimate the contribution of biological particles to the total atmospheric aerosol. As a result, the fractions of sodium-containing spores reported here represent lower limits for sodium-bearing fungal spores.

Presence of sodium in fungal spores. Previous studies highlighted the presence of biogenic potassium-rich particles in the Amazonia in the accumulation mode (0.1-1µm diameter)[22]. However, biological particles have never been indicated as a significant source of sodium-containing particles in the Amazonia. This is despite the fact that sodium in biological particles has been reported previously.[20,28] For example, laser-induced breakdown spectroscopy measurements showed the presence of sodium in fungal spores[28] and an X-ray microanalysis study

observed sodium in particles of biogenic origin[29]. The presence of sodium is common in so called halophilic fungi which cannot grow without NaCl[30]. Active discharge, uptake, and efflux processes are likely responsible for the sodium content in the spores and sodium content may vary with different classes and genera of fungi. During active discharge, fungi forcibly eject spores into the atmosphere, together with osmotic fluid containing hexoses, mannitol, phosphate, sodium, and potassium[22,31]. Fungi require K^+ for electrical and osmotic equilibria of the cells and presence of K^+ in several fungal spores is well understood[32]. Previous studies suggested that K^+ can be partially replaced by Na^+ and Na^+ can enhance the growth of fungi[33] and plants[34] under K^+ deficiency conditions. The growth of fungi and uptake of Na^+ varies with their physiological conditions and uptake rates depend on the species and their genes[35]. For example, specific genes (e.g., acu1 and acu2) are responsible for high-affinity Na^+ uptake of Ustilago maydis. When fungi contain excess Na^+, they activate Na^+-efflux ATPase (Adenosinetriphosphatase), which acts as a key enzyme for the biological evolution of fungi[36]. We suggest that uptake and efflux of Na^+ varies with different classes and genera of fungal community. For example, previous studies in Amazonia shows several genera (e.g., Agaricus; Amanita; Aspergillus; Boletus; Cladonia; Mortierella; Puccinia; Lepsita; and Rhizopus) within one class of fungi (Lecanonomycetes)[37,38]. Furthermore, the transpiration of plants[39] and nutrient uptake[40] can also influence the sodium content of spores. Further studies are needed to better comprehend the sodium content in fungal spores by linking chemical composition, molecular biology and diversity of fungal spores.

In contrast to previous reports[1,5], our analyses suggest that locally-emitted biological particles contribute substantially to the total concentration of sodium salt particles in the Amazon basin during the wet season. Backward trajectories calculated with NOAA's HYSPLIT model

suggest that marine air masses would have traveled ~1500 km over 2.5-3 days to reach central Amazonia (Supplementary Fig. 7). The rain records along backward trajectories also indicate that multiple precipitation events occurred during transport, which would likely wash out most of the sea salt particles before their arrival at the sampling site. Hence, these findings support the dominance of locally-emitted spores in the Amazon basin during this study. Furthermore, the presence of a substantial amount (48%±3%) of coarse mode sodium-rich particles below the ~20 m thick forest canopy suggests that local biological emissions contribute significantly to the concentration of sodium-salt particles in the Amazonia.

Model estimate of contribution of fungal spores to sodium budget. To evaluate the geographic distribution and frequency of high fungal spores contributions to airborne sodium mass over the Amazon basin, we conducted model simulations in the Community Earth System Model (CESM1.2.2, see Methods). Briefly, atmospheric emissions and transport simulations were performed using nudging[41] to recreate the observed meteorology of 2014-2015. Particulate sodium content was estimated by considering 70% of fungal spores to be sodium-rich (represents an upper bound), with sodium-rich fungal spores containing 13% sodium by mass, and 30% sodium in sea salt[42](see Methods). We also performed a sensitivity analysis by assuming either 30% or 50% of fungal spores to be sodium-rich.

Model-estimated and observed fungal spore concentrations in various ecosystems and geographic locations[9] (Supplementary Fig. 8) are compared in Fig. 3a. Estimated concentrations are typically higher than observed values but at the measurement site, model-estimated fungal spore concentrations are similar to the observed concentrations. Comparison between model-

estimated and measured sodium mass concentrations from sea salt emissions from various geographical locations at the coastal and island sites (Supplementary Fig. 9) are illustrated in Supplementary Fig. 10. The model estimates of sodium mass concentrations are approximately a factor of two higher than observations at many of the measurement sites; the measurements used in this analysis were all collected at coastal and island locations in the Southern hemisphere, between 20 and 70 S. It is interesting to note that measurements at the remote sites (e.g., Antarctica) are in good agreement with the estimated mass. The time series of both simulated fungal spore and sea salt concentrations (Supplementary Fig. 11) exhibited strong variability. Simulated sea salt concentrations, driven by the long-range transport of marine air, are higher during the dry season compared to the wet season. This may be due to stronger wet removal processes hindering long-range transport, and changes in wind patterns during the wet season.

In the model, during periods of low sea salt aerosol concentration, fungal spores typically contribute the majority of the estimated sodium mass. These conditions occur more frequently during the wet season (Fig. 3b) than the dry season (Supplementary Fig. 12). On average, fungal spores are estimated to contribute 69% of sodium during the wet season. We calculate approximate upper and lower bounds for the fungal spore contribution to the total sodium mass by varying fungal spore mass concentrations by a factor of ten and sea salt mass concentrations by a factor of two. On average, fungal spores are estimated to contribute 69% (31-95%) of sodium during the wet season. However, the level of confidence in fungal spore estimates is arguably higher for the Amazon, since the emissions parameterization was originally developed based primarily on measurements from the Amazon and other tropical rainforests, and simulated fungal spore number concentrations agree with measurements at the Amazonia site within a factor of two. Assuming a

factor of two uncertainty bounds for fungal spore concentrations, their estimated contribution to sodium mass would range between 51-84%. We performed a sensitivity analysis by assuming either 30% or 50% of fungal spores to be sodium-rich. When applying these different assumptions to the model simulations, fungal spores account for 58% (21-90%) or 65% (27-93%) of the total simulated sodium mass during the wet season (Supplementary Fig. 13). The model suggests that the fractional contribution of fungal spores to sodium will increase during nighttime and decrease during daytime (Fig. 3b). This may be the combined result of diurnal cycles in emissions, boundary-layer dynamics, and removal processes (e.g., precipitation). Fig. 3c presents a regional map of the percentage of days when fungal spores contribute at least 50% of estimated total sodium during the wet season.

Discussion

Overall, fungal spores dominated the number fraction of coarse mode in the Amazonia during nighttime and in below-canopy samples, and half of the coarse mode particles are sodium-rich. Previously, sodium-rich particles have been solely attributed to marine aerosol from the Atlantic Ocean. Previous studies in different atmospheric environment show the presence of sodium in fine mode and suggest potassium as a tracer for anthropogenic sodium[6-8,43,44]. Observations of our study are fundamentally different. First, our study focuses on the coarse mode particles. Second, the potassium observed in our study mostly originates from biological particles[22] confirmed by their unique and distinguishable morphology. Those particles were especially abundant during the wet season when biomass burning is not a dominant source of particles in the basin. Remarkably, our experimental and modeling results demonstrate that fungal spores emitted from the rainforest biosphere contribute a major fraction to the sodium budget during wet season, while most of

marine aerosol particles would be removed by wet deposition during transport. Fig. 4 illustrates the sources and atmospheric processing of fungal spores. Hygroscopicity experiments indicate that during cloud processing or high-humidity spores rupture and release submicrometer-to-micrometer size salt fragments[18], further contributing to the sodium budget in accumulation mode particles. This work highlights the potential importance of biological particles as a source of sodium-salt particles in widespread tropical forests, and motivates the necessity of studies investigating the chemistry of biological particles in other forested regions of the globe.

Acknowledgements

This research was supported by the Office of Science of the U.S. Department of Energy (DOE) as part of the Atmospheric System Research program and by the Environmental Molecular Sciences Laboratory (EMSL), a national scientific user facility sponsored by the DOE's Office of Biological and Environmental Research at Pacific Northwest National Laboratory (PNNL). The Pacific Northwest National Laboratory (PNNL) group acknowledges additional support from the Chemical Imaging Initiative of the Laboratory Directed Research and Development program at PNNL. The CCSEM/EDX, ESEM and NanoSIMS analyses, and the Community Earth System Model (CESM) simulations were performed at EMSL/PNNL. PNNL is operated for the U.S. DOE by Battelle Memorial Institute under contract DE-AC06-76RL0. STXM/NEXAFS analysis was performed at beamline 11.0.2.2 of the Advanced Light Source at Lawrence Berkeley National Laboratory. The beamline is supported by the Director, Office of Science, Office of Basic Energy Sciences of the U.S. DOE under Contract No. DE-AC02- 05CH11231. Office of Basic Energy Sciences Division of Chemical Sciences, Geosciences, and Biosciences by the Condensed Phase and Interfacial Molecular Sciences Program of the U.S. DOE are acknowledged for additional

support of the beamline. J. W. and T. H. acknowledge the student exchange program between the University of Würzburg and U.C. Berkeley (curator Professor A. Forchel, Würzburg and NSF IGERT program at UCB, DGE-0333455, Nanoscale Science and Engineering - From Building Blocks to Functional Systems). The Brazilian group acknowledges support from the FAPESP projects 08/58100-2, 2013/25058-1 and 2013/05014-0. We thank Simone R. da Silva and the LBA central office at the Instituto Nacional de Pesquisas da Amazônia (INPA) for assistance and logistical support during sampling at the field site. The CESM project is supported by the National Science Foundation and the Office of Science (BER) of the U.S. Department of Energy. We thank Prof. J. Prospero (U. Miami) and the AEROCOM project for collecting and making available the in situ aerosol measurement network dataset. The authors gratefully acknowledge the NOAA Air Resources Laboratory (ARL) for the provision of the HYSPLIT transport and dispersion model and READY website (http://www.ready.noaa.gov) used in this publication.

Author contributions

S.C., S.B., M.K.G., P.A., and A.L. designed the study. S.C., B.W., T.H., J.W., J.C., A.L., and M.K.G. performed the micro-spectroscopy experiments and analysis. A.L. and G.G.C. collected the samples. S.B., and P.M., performed the modeling work. M.T. gathered observed spore counts data. L.V.R performed backward trajectory analysis. L.V.R., and J.B. analyzed size distribution data. S.C. led the manuscript preparation with contributions from all co-authors.

Competing financial interests

The authors declare no competing financial interests.

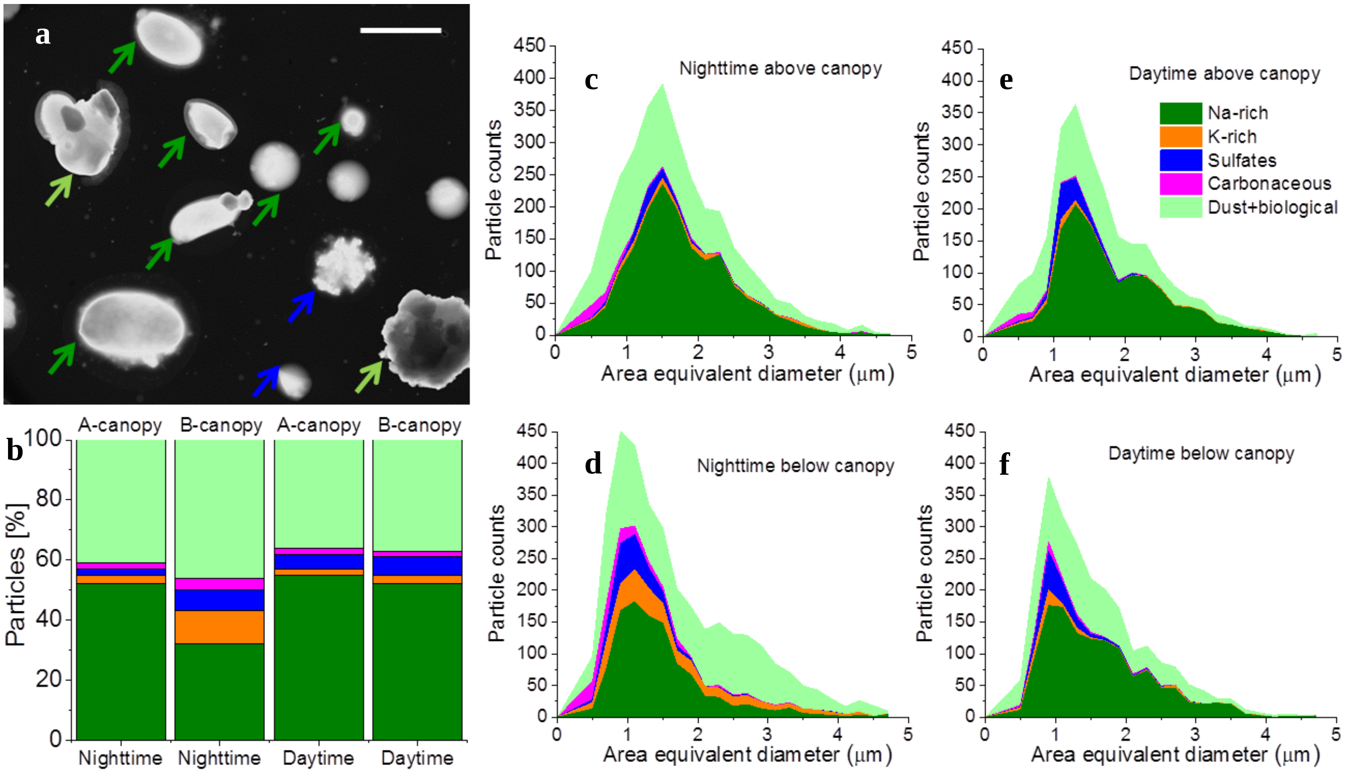

Figure 1| Size distribution and particle-type classes. a, Representative SEM image (forward scattered transmistted electron imaging mode) of particles collected at the ZF2 site in Amazonia. Dark green arrows indicate Na-rich particles, blue arrow indicates a mixed sulfate and Na containing particle with minor contributions of Na, and light green color arrows indicate other types of particles, mostly internally mixed dust and biological particles. Scale bar is 5 μm. **b**, Nightime and daytime number fraction of different particle classes for particles collected above canopy (A-canopy) and below canopy (B-canopy). **c-f** Size distribution of different particle classes.

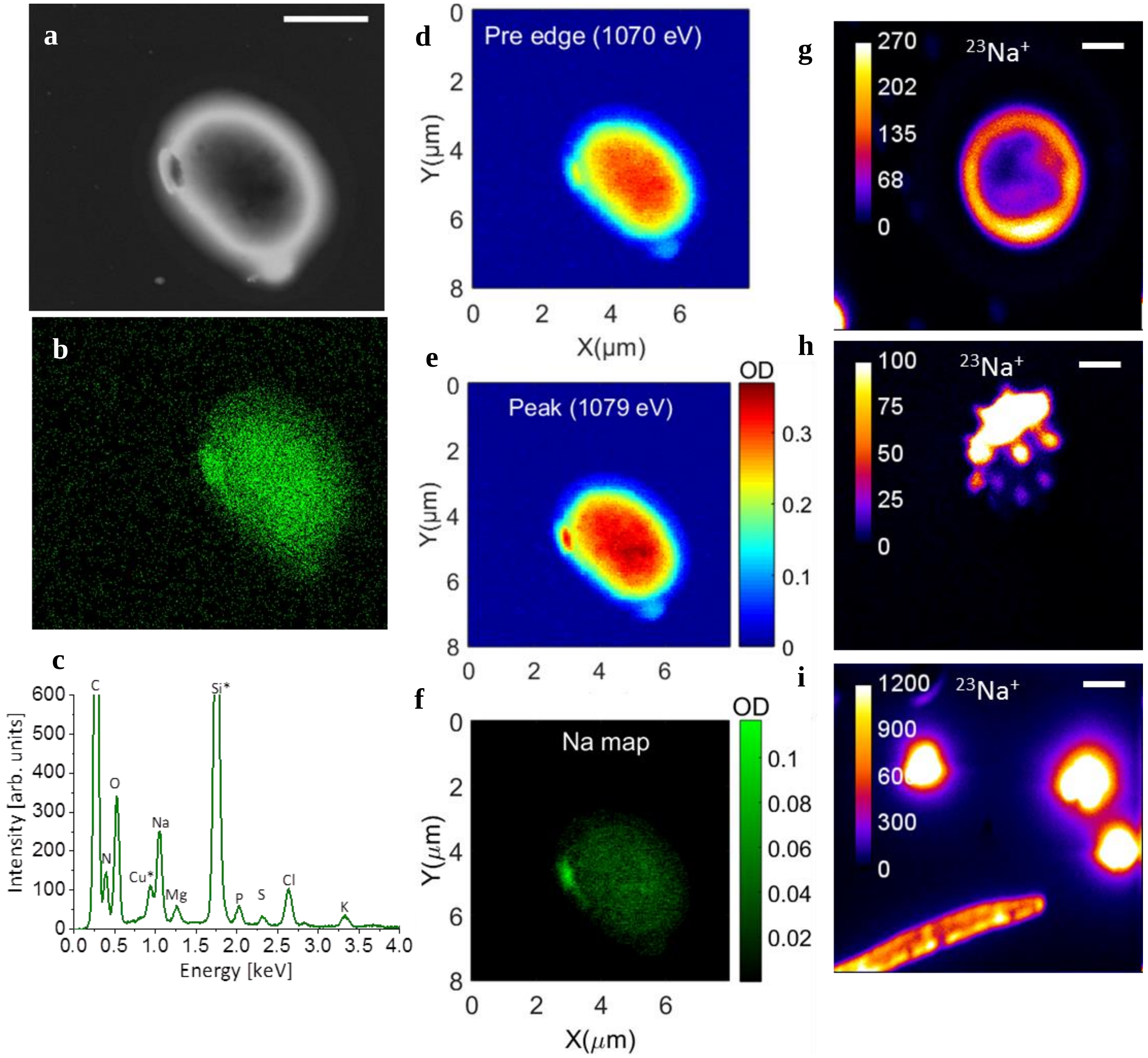

Figure 2| Chemical imaging of Na-containing biological particles. a, SEM image **b**, elemental Na map **c**, EDX spectra of the biological particle. The Cu and Si in the EDX spectra are background peaks originating from the substrate and various instrument parts inside the SEM chamber. STXM images of the same biological particle: **d**, pre-edge (1070 eV), **e**, Na peak (1079 eV) and **f**, Na optical density map. **g-i**, Representative NanoSIMS images of $^{23}Na^+$ of selected biological particles showing various distributions of sodium within particles contours. Scale bars are 2 μm.

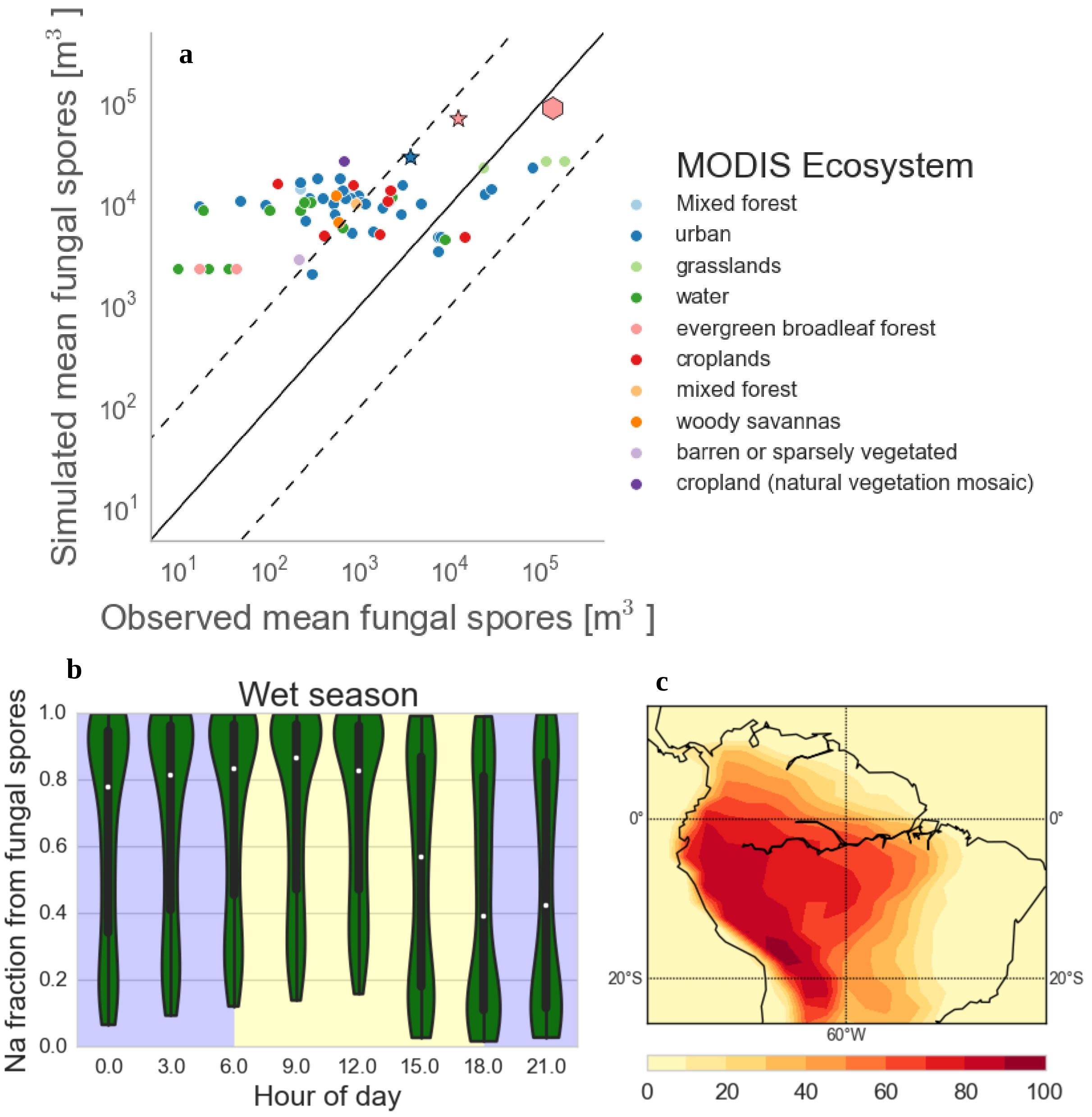

Figure 3| Simulation of sodium contribution from biological particles to total sodium budget in the Amazon area. a, Comparison of model-simulated and observed mean fungal spore concentration at various ecosystem and geographical locations. All model-simulated values are annual means; while experimental observations are mostly mean values over periods from 2 months up to 3 years, as previously reported in the literature (full reference list is provided in Supplementary Table 2). Dotted lines represent an over-or under-estimate of a factor of ten (10:1

and 1:10). The pink hexagonal point represents the measurement campaign (mean simulated fungal spore concentration, Jan 26 – Feb 8, 2015, compared with observed fungal spore concentrations during the same time period). The stars indicate points from literature measurements from tropical rainforests. **b,** Sodium fraction from fungal spores in wet and dry season at the model's nearest grid point to the ZF2 tower, located at 2.84° S 60.0° W, in the model's lowest (near-surface) layer. White dots represent the median, thick lines represent 25-75 percentile, and thin vertical lines represent the range of minimum and maximum value; violin plot represents a kernel density estimate distribution of the sodium fraction values. Background shading indicates time of day, blue represents night [1800-0600] and yellow represents day [0600-1800]. **c,** Percentage of days when fungal spores contributed at least 50% of estimated total sodium in wet season, assuming that 70% of fungal spores are sodium-rich, containing 13% sodium by mass, and sea salt aerosol is composed of 30% Na by mass. Results for dry season are shown in Supplementary Fig. 12.

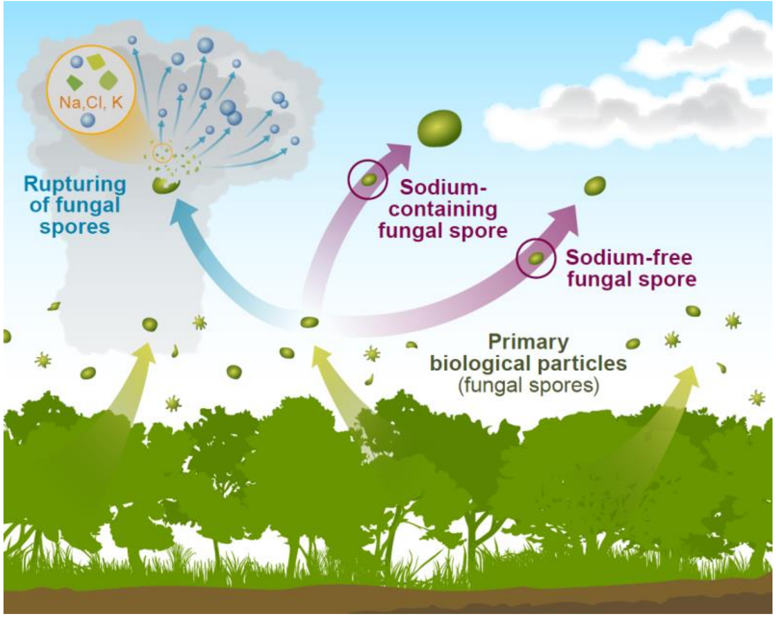

Figure 4| Sources and atmospheric processing of fungal spore particles in the Amazon rainforest. Sodium-containing and sodium-free fungal spore particles are emitted from the Amazon rainforest. Sodium-containing fungal spores exhibit higher hygroscopic growth compared to sodium-free fungal spores. When they are exposed to high humidity conditions, or through cloud processing, fungal spore particles rupture and release submicrometer-to-micrometer size fragments. A substantial fraction of the fragments which contains Na, Cl, and K, and appear morphologically similar to dry sea salt particles. These hygroscopic salt fragments can participate in cloud formation.

Data Availability

All relevant data are available from the authors.

References

1 Martin, S. T. *et al.* Sources and properties of Amazonian aerosol particles. *Reviews of Geophysics* **48**, RG2002, doi:10.1029/2008RG000280 (2010).

2 Roberts, G. C., Andreae, M. O., Zhou, J. & Artaxo, P. Cloud condensation nuclei in the Amazon Basin:"Marine" conditions over a continent? *Geophysical research letters* **28**, 2807-2810 (2001).

3 Williams, E. *et al.* Contrasting convective regimes over the Amazon: Implications for cloud electrification. *Journal of Geophysical Research: Atmospheres* **107**, 8082, doi:10.1029/2001JD000380 (2002).

4 Artaxo, P. *et al.* Atmospheric aerosols in Amazonia and land use change: from natural biogenic to biomass burning conditions. *Faraday discussions* **165**, 203-235 (2013).

5 Talbot, R. W. *et al.* Aerosol chemistry during the wet season in central Amazonia: The influence of long-range transport. *Journal of Geophysical Research: Atmospheres* **95**, 16955-16969, doi:10.1029/JD095iD10p16955 (1990).

6 Ooki, A., Uematsu, M., Miura, K. & Nakae, S. Sources of sodium in atmospheric fine particles. *Atmospheric Environment* **36**, 4367-4374, doi:https://doi.org/10.1016/S1352-2310(02)00341-2 (2002).

7 Mamane, Y. Estimate of municipal refuse incinerator contribution to philadelphia aerosol-I. Source analysis. *Atmospheric Environment (1967)* **22**, 2411-2418, doi:https://doi.org/10.1016/0004-6981(88)90473-8 (1988).

8 Bourotte, C. *et al.* Association between ionic composition of fine and coarse aerosol soluble fraction and peak expiratory flow of asthmatic patients in São Paulo city (Brazil). *Atmospheric Environment* **41**, 2036-2048, doi:https://doi.org/10.1016/j.atmosenv.2006.11.004 (2007).

9 Sesartic, A. & Dallafior, T. N. Global fungal spore emissions, review and synthesis of literature data. *Biogeosciences* **8**, 1181-1192, doi:10.5194/bg-8-1181-2011 (2011).

10 Jacobson, M. Z. & Streets, D. G. Influence of future anthropogenic emissions on climate, natural emissions, and air quality. *Journal of Geophysical Research: Atmospheres* **114**, n/a-n/a, doi:10.1029/2008JD011476 (2009).

11 Després, V. R. *et al.* Primary biological aerosol particles in the atmosphere: a review. *Tellus B* **64**, doi:10.3402/tellusb.v64i0.15598 (2012).

12 Pringle, A., Patek, S. N., Fischer, M., Stolze, J. & Money, N. P. The captured launch of a ballistospore. *Mycologia* **97**, 866-871 (2005).

13 Elbert, W., Taylor, P. E., Andreae, M. O. & Pöschl, U. Contribution of fungi to primary biogenic aerosols in the atmosphere: wet and dry discharged spores, carbohydrates, and inorganic ions. *Atmos. Chem. Phys.* **7**, 4569-4588, doi:10.5194/acp-7-4569-2007 (2007).

14 Twohy, C. H. *et al.* Abundance of fluorescent biological aerosol particles at temperatures conducive to the formation of mixed-phase and cirrus clouds. *Atmos. Chem. Phys.* **16**, 8205-8225, doi:10.5194/acp-16-8205-2016 (2016).

15 Griffin, D. W. Terrestrial Microorganisms at an Altitude of 20,000 m in Earth's Atmosphere. *Aerobiologia* **20**, 135-140, doi:10.1023/b:aero.0000032948.84077.12 (2004).

16 Huffman, J. A. *et al.* Size distributions and temporal variations of biological aerosol particles in the Amazon rainforest characterized by microscopy and real-time UV-APS fluorescence techniques during AMAZE-08. *Atmos. Chem. Phys.* **12**, 11997-12019, doi:10.5194/acp-12-11997-2012 (2012).

17 Haga, D. I. *et al.* Ice nucleation by fungal spores from the classes Agaricomycetes, Ustilaginomycetes, and Eurotiomycetes, and the effect on the atmospheric transport of these spores. *Atmos. Chem. Phys.* **14**, 8611-8630, doi:10.5194/acp-14-8611-2014 (2014).

18 China, S. *et al.* Rupturing of Biological Spores As a Source of Secondary Particles in Amazonia. *Environmental Science & Technology* **50**, 12179-12186, doi:10.1021/acs.est.6b02896 (2016).

19 Steiner, A. L. *et al.* Pollen as atmospheric cloud condensation nuclei. *Geophysical Research Letters* (2015).

20 Ebert, M., Weinbruch, S., Hoffmann, P. & Ortner, H. M. The chemical composition and complex refractive index of rural and urban influenced aerosols determined by individual particle analysis. *Atmospheric Environment* **38**, 6531-6545, doi:http://dx.doi.org/10.1016/j.atmosenv.2004.08.048 (2004).

21 Zhisheng, Z. *et al.* Significant influence of fungi on coarse carbonaceous and potassium aerosols in a tropical rainforest. *Environmental Research Letters* **10**, 034015 (2015).

22 Pöhlker, C. *et al.* Biogenic potassium salt particles as seeds for secondary organic aerosol in the Amazon. *Science* **337**, 1075-1078 (2012).

23 Guyon, P. *et al.* In-canopy gradients, composition, sources, and optical properties of aerosol over the Amazon forest. *Journal of Geophysical Research: Atmospheres* **108**, 4591, doi:10.1029/2003JD003465 (2003).

24 Gabey, A. M. *et al.* Measurements and comparison of primary biological aerosol above and below a tropical forest canopy using a dual channel fluorescence spectrometer. *Atmos. Chem. Phys.* **10**, 4453-4466, doi:10.5194/acp-10-4453-2010 (2010).

25 Rizzo, L. V., Artaxo, P., Karl, T., Guenther, A. B. & Greenberg, J. Aerosol properties, in-canopy gradients, turbulent fluxes and VOC concentrations at a pristine forest site in Amazonia. *Atmospheric Environment* **44**, 503-511, doi:https://doi.org/10.1016/j.atmosenv.2009.11.002 (2010).

26 Boxer, S. G., Kraft, M. L. & Weber, P. K. Advances in imaging secondary ion mass spectrometry for biological samples. *Annual review of biophysics* **38**, 53-74 (2009).

27 Piens, D. S. *et al.* Measuring Mass-Based Hygroscopicity of Atmospheric Particles through in Situ Imaging. *Environmental Science & Technology* **50**, 5172-5180, doi:10.1021/acs.est.6b00793 (2016).

28 Hybl, J. D., Lithgow, G. A. & Buckley, S. G. Laser-induced breakdown spectroscopy detection and classification of biological aerosols. *Appl. Spectrosc.* **57**, 1207-1215 (2003).

29 Krejci, R., Ström, J., de Reus, M. & Sahle, W. Single particle analysis of the accumulation mode aerosol over the northeast Amazonian tropical rain forest, Surinam, South America. *Atmos. Chem. Phys.* **5**, 3331-3344, doi:10.5194/acp-5-3331-2005 (2005).

30 Plemenitaš, A. *et al.* Adaptation to high salt concentrations in halotolerant/halophilic fungi: a molecular perspective. *Frontiers in Microbiology* **5**, doi:10.3389/fmicb.2014.00199 (2014).
31 Webster, J. *et al.* Mannitol and hexoses are components of Buller's drop. *Mycological Research* **99**, 833-838, doi:http://dx.doi.org/10.1016/S0953-7562(09)80737-5 (1995).
32 Rodríguez-Navarro, A. Potassium transport in fungi and plants. *Biochimica et Biophysica Acta (BBA) - Reviews on Biomembranes* **1469**, 1-30, doi:https://doi.org/10.1016/S0304-4157(99)00013-1 (2000).
33 Camacho, M., Ramos, J. & Rodríguez-Navarro, A. Potassium requirements ofSaccharomyces cerevisiae. *Current Microbiology* **6**, 295-299, doi:10.1007/bf01566880 (1981).
34 Subbarao, G. V., Wheeler, R. M., Stutte, G. W. & Levine, L. H. How far can sodium substitute for potassium in red beet? *Journal of Plant Nutrition* **22**, 1745-1761, doi:10.1080/01904169909365751 (1999).
35 Benito, B., Garciadeblás, B., Schreier, P. & Rodríguez-Navarro, A. Novel P-type ATPases mediate high-affinity potassium or sodium uptake in fungi. *Eukaryotic Cell* **3**, 359-368 (2004).
36 Benito, B., Garciadeblás, B. & Rodríguez-Navarro, A. Potassium- or sodium-efflux ATPase, a key enzyme in the evolution of fungi. *Microbiology* **148**, 933-941, doi:doi:10.1099/00221287-148-4-933 (2002).
37 Womack, A. M. *et al.* Characterization of active and total fungal communities in the atmosphere over the Amazon rainforest. *Biogeosciences* **12**, 6337-6349, doi:10.5194/bg-12-6337-2015 (2015).
38 Fröhlich-Nowoisky, J. *et al.* Biogeography in the air: fungal diversity over land and oceans. *Biogeosciences* **9**, 1125-1136, doi:10.5194/bg-9-1125-2012 (2012).
39 Nemeryuk, G. Migration of salts into the atmosphere during transpiration. *Soviet Plant Physiol* (1970).
40 Lawson, I. T., Jones, T. D., Kelly, T. J., Coronado, E. N. H. & Roucoux, K. H. The geochemistry of Amazonian peats. *Wetlands* **34**, 905-915 (2014).
41 Zhang, K. *et al.* Technical Note: On the use of nudging for aerosol–climate model intercomparison studies. *Atmos. Chem. Phys.* **14**, 8631-8645, doi:10.5194/acp-14-8631-2014 (2014).
42 Pilson, M. An Introduction to the Chemistry of the Sea Prentice-Hall. *Inc. Upper Saddle River, NJ. 431p* (1998).
43 Kitto, M. E. Trace-Element Patterns in Fuel Oils and Gasolines for Use in Source Apportionment. *Air & Waste* **43**, 1381-1388, doi:10.1080/1073161X.1993.10467213 (1993).
44 Mizohata, A. & Mamuro, T. Chemical element balances and identification of air pollution sources in Sakai, Osaka (I). *Journal of Japan Society of Air Pollution* **15**, 198-206 (1980).
45 Bauer, H. *et al.* Determination of the carbon content of airborne fungal spores. *Analytical Chemistry* **74**, 91-95 (2002).
46 Moffet, R., AV, T. & Gilles, M. in *Fundamentals and Applications in Aerosol Spectroscopy* (eds R Signorell & JP Reid) 419–462 (Taylor & Francis, 2010).

47 Heald, C. L. & Spracklen, D. V. Atmospheric budget of primary biological aerosol particles from fungal spores. *Geophysical Research Letters* **36**, L09806, doi:10.1029/2009GL037493 (2009).
48 Neale, R. B. *et al.* Description of the NCAR community atmosphere model (CAM 5.0). *NCAR Tech. Note NCAR/TN-486+ STR* (2010).
49 Hurrell, J. W. *et al.* The Community Earth System Model: A Framework for Collaborative Research. *Bulletin of the American Meteorological Society* **94**, 1339-1360, doi:10.1175/bams-d-12-00121.1 (2013).
50 Ma, P.-L. *et al.* The role of circulation features on black carbon transport into the Arctic in the Community Atmosphere Model version 5 (CAM5). *Journal of Geophysical Research: Atmospheres* **118**, 4657-4669, doi:10.1002/jgrd.50411 (2013).
51 Rienecker, M. M. *et al.* MERRA: NASA's Modern-Era Retrospective Analysis for Research and Applications. *Journal of Climate* **24**, 3624-3648, doi:10.1175/jcli-d-11-00015.1 (2011).
52 Liu, X. *et al.* Toward a minimal representation of aerosols in climate models: description and evaluation in the Community Atmosphere Model CAM5. *Geosci. Model Dev.* **5**, 709-739, doi:10.5194/gmd-5-709-2012 (2012).
53 Mårtensson, E. M., Nilsson, E. D., de Leeuw, G., Cohen, L. H. & Hansson, H. C. Laboratory simulations and parameterization of the primary marine aerosol production. *Journal of Geophysical Research: Atmospheres* **108**, 4297, doi:10.1029/2002JD002263 (2003).
54 Monahan, E. C. in *The Role of Air-Sea Exchange in Geochemical Cycling* 129-163 (Springer, 1986).
55 Textor, C. *et al.* Analysis and quantification of the diversities of aerosol life cycles within AeroCom. *Atmos. Chem. Phys.* **6**, 1777-1813, doi:10.5194/acp-6-1777-2006 (2006).
56 Pope, F. D. Pollen grains are efficient cloud condensation nuclei. *Environmental Research Letters* **5**, 044015 (2010).
57 Pöhlker, M. L. *et al.* Long-term observations of cloud condensation nuclei in the Amazon rain forest – Part 1: Aerosol size distribution, hygroscopicity, and new model parametrizations for CCN prediction. *Atmos. Chem. Phys.* **16**, 15709-15740, doi:10.5194/acp-16-15709-2016 (2016).

Methods

Sampling Site and Sample Collection. Samples were collected during the wet season (January and February, 2015) at the ZF2 Tower (02°35.3517′ S, 60 06.8333′ W), a pristine rainforest site in Central Amazonia located 40 km North of Manaus margins. Samples were collected through a sampling inlet located at a height of 2 meters (below canopy) and 39 m above ground (above canopy), during day and night with 30 L/min sampling rate. Particles were collected onto 400 mesh

transmission electron microscopy (TEM) grids coated with Carbon Type-B films (Ted Pella, Inc.), silicon nitride membrane substrates (0.5 × 0.5 mm^2 Si_3N_4 window size, 100 nm membrane thickness, 5 × 5 mm^2 Si frame size; Silson, Inc.). 10-stage Micro-Orifice Uniform Deposition Impactors™ (MOUDI™; model 110-R, MSP, Inc.) were used for sampling. This study focuses on the samples from stages 4 and 5 (size range: 1.0-3.2 µm) where the relative abundance of biological particles is high. All the experiments were performed with diligently. Samples were handled with caution. For examples, tweezers were cleaned with solvents prior to handling the samples; new gloves were used for handling of samples after each experiments.

Chemical imaging of particles. An environmental scanning electron microscope (ESEM, Quanta 3D model, FEI, Inc.) with an EDAX™ energy dispersive X-ray (EDX) spectrometer and a Si(Li) detector with a 10 mm^2 active area and an ATW2 window was used under vacuum conditions for imaging and X-ray microanalysis. Particles were imaged using secondary electron (SE) and forward scattered transmitted electron (STE) signals. STE imaging mode was used for particle identification and computer-controlled (CC) SEM/EDX analysis. X-ray spectrum for each identified particle was acquired at an acceleration voltage of 20 kV and at a beam current of 430 pA for 10 seconds. The computer-controlled CCSEM/EDX analysis automatically investigates the specified scanning area and detects particles in the specified fields of view. In this way, we can detect ~90% of the particles deposited on the grid. Particles collected on the edges (~5-10%) of the Cu mesh were excluded from the results based on their high Cu background signal. We analyzed ~80% of the particles that were collected onto TEM B-film grids placed on stages 4 and 5 of the impactor. Area equivalent diameters (AED) were calculated from the 2D projected area recorded for each individual particle. Particle elemental composition was quantified and is reported in units of atomic fractions. The percentage of sodium content in fungal spores was estimated using

the known mass fraction of spores[13] and carbon/sodium wt% from X-ray microanalysis. Carbon content in spores ranges from 42-66%, with an average of 51%[45]. The average carbon/sodium wt% in fungal spores is 4:1. The mass of the spores is estimated from the size distribution of microscopy analysis and assuming a density of 1 gm cm^{-3})[13,16].

STXM utilizes a focused soft X-ray beam generated from the synchrotron light source to probe chemical bonding of specific elements of interest within individual particles. STXM images are obtained by raster scanning the sample at fixed photon energy and recording intensities of the transmitted X-rays at each pixel. The optical density ($-\ln(I(_d)/I_0)$) is calculated based on the measured intensity (I_d) using Beer-Lambert's law [46]. Transmission intensity through a particle free region of the substrate is used to obtain I_0.

The hygroscopicity of biological particles was investigated using a temperature-controlled cooling stage in the environmental SEM to estimate the area growth factor (ratio of wet-to-dry diameters) and a microreactor for in situ STXM hydration experiments[27] to estimate the mass growth factor (ratio of wet-to-dry oxygen mass).

Nano secondary ion mass spectrometry (NanoSIMS, CAMECA 50L, Gennevilliers Cedex, France) was used to perform imaging of Na+ ions. NanoSIMS provides high lateral resolution. Samples were coated with a thin layer of Au to minimize charging and non-equilibrium sputtering effects during analysis. A 16 keV O^- primary ion beam was used for analysis. Prior to data collection, samples were pre-sputtered with about 10^{16} O^- ions cm^{-2}. Analysis of 15 µm × 15 µm and 256 px × 256 px were performed with a dwell time of 13.5 ms px^{-1}. Beam diameter was estimated to be about 250 nm.

Community Atmosphere Model Description. Fungal spore emissions were calculated according to the global model parameterization of Heald and Spracklen[47]. While this parameterization has substantial uncertainties, it was developed on the basis of observed mannitol concentrations from various continental locations and seasons; about half of these observations were from PM2.5 aerosol collected in the tropical Brazilian rainforest during the wet season, where concentrations were 2-3 times higher than those reported at extratropical locations[13]. Therefore, we believe the parameterization can be used with greater confidence in the Amazonian rainforest, particularly during the wet season, than at other times and locations.

A simulation for present-day conditions was performed using the Community Atmosphere Model (CAM5)[48], which is the atmosphere component of the Community Earth System Model (CESM, version 1.2.1)[49]. The model was configured at a horizontal resolution of 1.9° latitude by 2.5° longitude, with 30 vertical layers ranging from the surface to 2.26 hPa. The simulation was performed from March 2014 to March 2015 with constrained meteorology [50],where model winds are nudged toward the MERRA reanalysis[51] with a relaxation time of 6 hours[41].

The model tracks the emissions and concentrations of six chemical species of aerosol: sea salt, dust, sulfate, secondary organic aerosol (SOA), black carbon (BC) and continental primary organic aerosol (POA). Aerosol transport and microphysics, including nucleation, condensation, and coagulation, is calculated within a modal aerosol module with three size classes (MAM3)[52], including the Aitken mode (dry diameter size range of 0.02–0.08 μm), accumulation mode (0.08–1.0 μm), and coarse mode (1.0–10.0 μm). Sea salt aerosol emission follows Mårtensson et al[53] for small particles (diameter < 2.8 μm), while emissions of larger particles (diameter > 2.8 μm) follow Monahan et al[54]. The simulated sea salt budget was evaluated in Liu et al[52] and falls within the

AeroCom values[55]; the modeled global sea salt emission flux is within the range of 2200 ~ 118,000 Tg yr^{-1} using different sea salt source functions. A detailed description of the model's aerosol removal processes can be found elsewhere[48].

Fungal spores were emitted into the model's coarse mode, and are removed by wet and dry deposition in the same manner as other aerosol species. Upon emission both fungal spore mass and coarse mode number are increased, with the mass-to-number ratio for emissions determined by assuming that fungal spores have 4 μm diameter upon emission. The model's coarse mode particle number concentration also includes number contributions from other aerosol species, which are treated as internally mixed within the coarse mode, and the particle diameter is calculated dynamically by the aerosol microphysics routines from the total coarse mode aerosol mass and number, and a fixed geometric width. Fungal spores were assigned the same optical properties as the model's organic carbon tracer, which is close to the observed refractive indices of fungal spores (1.4-0i)[20]. Fungal spores were assigned a material density of 1 g cm^{-3}, which is commonly used in literature[13,16]. Fungal spores were assigned a hygroscopicity parameter (κ) of 0.1, similar to that of pollen grains[56]. The observed overall κ value for ambient below-canopy aerosol in the Amazon is 0.22±0.05 in the accumulation mode, with an overall mean value of κ=0.17±0.06[57].

We thank the reviewers' for their comments and suggestions. In the following discussion, the reviewers' comments are in black-colored font, our responses are in blue-colored font, and changes in the manuscript are noted by *italics blue-colored font.*

Reviewer #2 (Remarks to the Author):

As I pointed out in my initial review, there are many non-marine sources of sodium salts, which the manuscript failed to discuss. It is frustrating to me that the authors acknowledge this fact in their response and yet refuse to improve the text accordingly. A number of relevant references are missing. In fact, the authors discuss a number of these in their rebuttal but don't include them in the revised text. I find the manuscript seriously lacking in this regard. It is a serious problem in the discussion and introduction. It would seem that the authors are concerned that once the proper references are cited, the novelty of their manuscript will fall beneath the high standards of Nature. Additionally, failure to acknowledge and discuss Na sources is a weakness in the estimates of how much salt in the Amazon comes from marine sources. I appreciate the effort in revising and adding the modeled estimates of sources. However, that section is very simplistic and I question the merit of conclusions drawn from it. Also, the authors state in their response that other Na sources in aerosol are "completely different" because they are small sized aerosols, whereas fungal spores are in the coarse mode. This statement is contradictory to the discussion in the manuscript text that describes the bursting of fungal spores and generation of smaller aerosol as a major reason that fungal spores have climatic relevance. What is known in the literature about the concentration of NaCl in spores? I suspect that a lot more work has been conducted that is not cited here.

In response to the reviewer's request, we added relevant text in the introduction as noted below (lines: 58-68). Regarding the bursting of fungal spores and generation of smaller particles, we suggest that total contribution of sodium budget from fungal spores can be even lager because we didn't take into account the smaller particles in this study. This study focuses solely on the coarse mode particles and reported results from samples collected on stages 4 and 5 (size range: 1.0-3.2 µm) where the relative abundance of biological particles is high.

"*This study shows locally emitted fungal spores of coarse super-micrometer size contribute considerably to sodium salt particles in the Amazon basin during wet season. While previous studies reported non-marine sources of sodium salts in fine particles, sodium-containing coarse particles have been attributed typically to sea-salt. Specifically, Ooki et al*[1] *observed the existence of anthropogenic sodium in fine mode (<1.1 µm) aerosol particles in urban air. Ooki et al*[1] *suggested potassium as a tracer for anthropogenic sodium in submicron size (0.43µm) particles. Mamane*[2] *found presence of sodium and potassium in <1 µm particles from waste incinerators emissions. Another study conducted in the metropolitan area of São Paulo found K/Na ratio of 1.4 and 0.9 for coarse (>2.5 µm) and fine (<2.5µm) particles respectively and they attributed this particles to waste incineration and vehicles*[3]."

We added the following text in the discussion (lines: 268-273)

"Previous studies in different atmospheric environment show the presence of sodium in fine mode and suggest potassium as a tracer for anthropogenic sodium[1-5]. Observations of our study are fundamentally different. First, our study focuses on the coarse mode particles. Second, the potassium observed in our study mostly originates from biological particles[6] confirmed by their unique and distinguishable morphology. Those particles were especially abundant during the wet season when biomass burning is not a dominant source of particles in the basin."

The experimental details are sparse. My concern that the highly variable present of salt may simply be a function of operator error (and the occasional contamination of some samples through contain with human skin or contaminated (or re-used) laboratory gloves. I note that another reviewer requested additional details, and the authors chose not to include them, but rather to refer readers to a previous manuscript. I appreciate the limitation of space, but this does not help to eleviate my concern that the experimental results are artifacts rather than new findings.

As we discussed in our revised manuscript, we suggest that variable present of sodium content may depend on different classes and genera of fungi. Sample collection and experiments were conducted with cautions. In our previous revised version as requested by the reviewer 3, we provided details of the method (please see the notes of the reviewer 3 who found our arguments convincing). In addition to that, we provide following discussions, which suggest that presence of sodium salt in our sample indeed from spores rather than contamination.

i) We routinely investigate quality of our blank substrates from different batches (total 5 in this case). SEM images at different field of view show that blank sample are with negligible contamination (see Supplementary Figure 14). We found 1 particle per 0.0005 cm^2 area, where as field collected particles, on average, consist of ~500 individual particles on stages 4 and 5. Particle loading is higher for smaller size particles (~2100 particles per 0.0005 cm^2 area on stages 6 and 7, see Supplementary Figure 15a, b).

ii) As we mentioned earlier, this study reports samples from stages 4 and 5 (size range: 1.0-3.2 µm). However, we also investigate particles from lower stages 6 and 7, size range: 0.32-1.0 µm) for a separate study. Fractions of Na-containing particles in stages 6 and 7 are significantly lower (Na-containing particle <15%) than particles on stages 4 and 5 (~50%). Please see Supplementary Figure 15c. This result suggest that high fraction of sodium-salt particles are indeed from spores that were deposited on stages 4 and 5.

iii) Na-content in spores were investigated using different techniques (electron microscopy and Nano- secondary ion mass spectrometry) with different operator, sample preparation and sample handling procedures. Irrespective of that, both methods show presence of sodium in spores.

To elaborate more on this note, we added two new Supplementary Figures (14 and 15) and the following text (lines: 555-564) in the method section of the revised manuscript.

"All the experiments were performed with diligently. Samples were handled with caution. For examples, tweezers were cleaned with solvents prior to handling the samples; new gloves were used for handling of samples after each experiments. Examination of blank substrate showed negligible contamination on the sample substrate (Supplementary Figure 14). Investigation of particles from stages 6 and 7 (size range: 0.32-1.0 μm) showed Na-containing particles in stages 6 and 7 are significantly lower (Na-containing particle <15%) than particles on stages 4 and (Supplementary Figure 15). This result suggest that high fraction of sodium-salt particles are indeed from spores that were deposited on stages 4 and 5. Furthermore, sodium content in spores was observed irrespective of different sample preparation and techniques (e.g., SEM/EDX, STXM and NanoSIMS)"

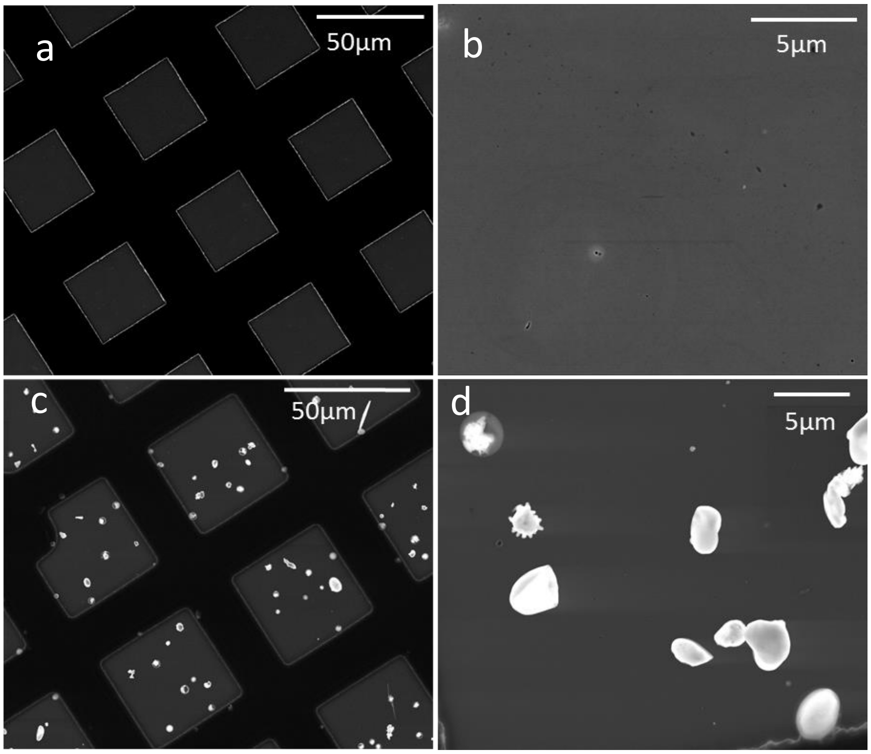

Supplementary Figure 14. Investigation of particle contamination. *SEM images show a) low magnification and b) high magnification image of blank sample; c) low magnification and d) high magnification image of collected sample.*

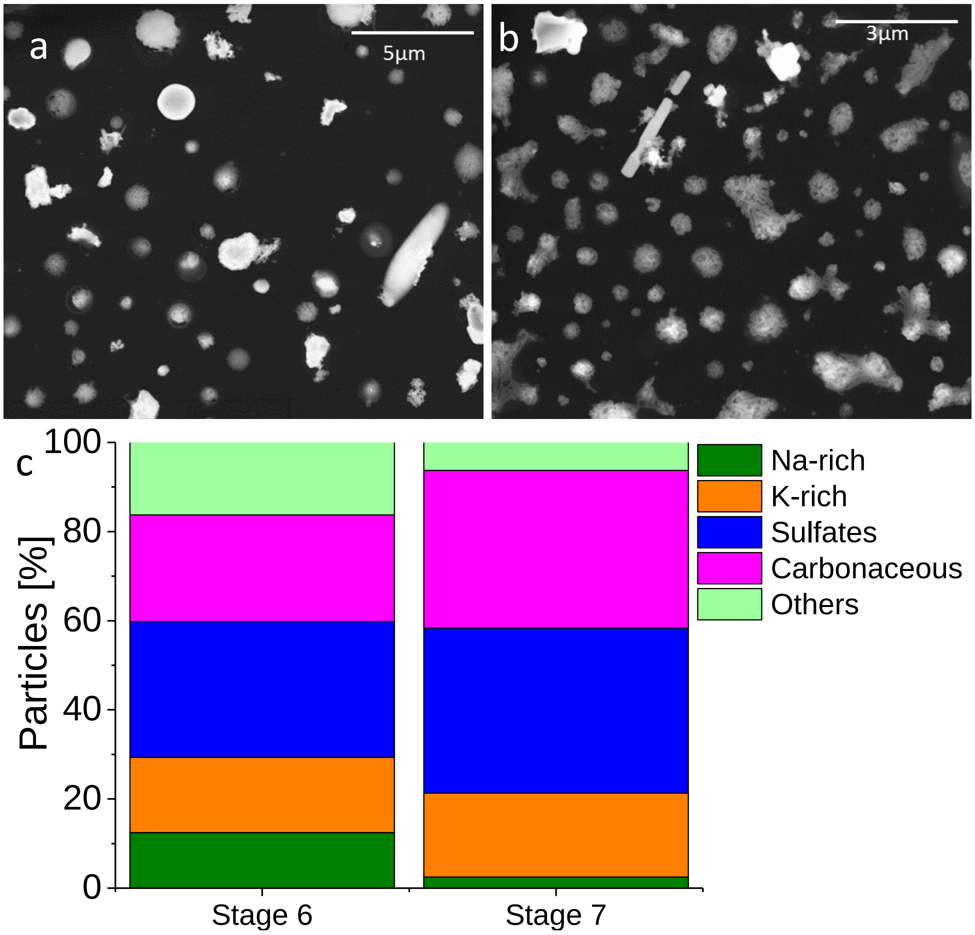

Supplementary Figure 15. Fraction of Na-rich particles in smaller size (stages 6 and 7) particles. *Representative SEM images of particles collected on a) stage 6 (0.56-1.0 µm), b) stage 7 (0.32-0.56 µm), c) number fraction of different particle classes for particles collected on stages 6 and 7. Fraction of Na-rich particles is <15%.*

Reviewer #3 (Remarks to the Author):

The authors have appropriately addressed the comments of me and the other two reviewers. I only have one (very minor) comment on the revised version of the MS: in the main text (lines 236-256), the authors discuss the uncertainty in the 69% contribution of fungal spores to sodium mass, due to assumptions on spore sodium content and sea salt contribution. I would like to see this uncertainty reflected in the number that is given in the abstract, so could you please indicate the uncertainty range there (line 36)?

My recommendation is therefore to publish the paper after this minor revision.

As suggested, we provided the uncertainty range in the abstract as well.

“Modeling results suggest that fungal spores account for ~69% (31-95%) of the total sodium mass during the wet season and that their fractional contribution increases during nighttime.”

References

1 Ooki, A., Uematsu, M., Miura, K. & Nakae, S. Sources of sodium in atmospheric fine particles. *Atmospheric Environment* **36**, 4367-4374, doi:https://doi.org/10.1016/S1352-2310(02)00341-2 (2002).

2 Mamane, Y. Estimate of municipal refuse incinerator contribution to philadelphia aerosol-I. Source analysis. *Atmospheric Environment (1967)* **22**, 2411-2418, doi:https://doi.org/10.1016/0004-6981(88)90473-8 (1988).

3 Bourotte, C. *et al.* Association between ionic composition of fine and coarse aerosol soluble fraction and peak expiratory flow of asthmatic patients in São Paulo city (Brazil). *Atmospheric Environment* **41**, 2036-2048, doi:https://doi.org/10.1016/j.atmosenv.2006.11.004 (2007).

4 Kitto, M. E. Trace-Element Patterns in Fuel Oils and Gasolines for Use in Source Apportionment. *Air & Waste* **43**, 1381-1388, doi:10.1080/1073161X.1993.10467213 (1993).

5 Mizohata, A. & Mamuro, T. Chemical element balances and identification of air pollution sources in Sakai, Osaka (I). *Journal of Japan Society of Air Pollution* **15**, 198-206 (1980).

6 Pöhlker, C. *et al.* Biogenic potassium salt particles as seeds for secondary organic aerosol in the Amazon. *Science* **337**, 1075-1078 (2012).

Fungal spores as a source of sodium salt particles in the Amazon basin

Swarup China[1], Susannah Burrows[2], Bingbing Wang[3], Tristan H. Harder[4,5,‖], Johannes Weis[4,5], Meryem Tanarhte[6], Luciana V. Rizzo[7], Joel Brito[8], Glauber G. Cirino[9], Po-Lun Ma[2], John Cliff[1], Paulo Artaxo[10], Mary K. Gilles[4], Alexander Laskin[11,*]

[1]*William R. Wiley Environmental Molecular Sciences Laboratory, Pacific Northwest National Laboratory, Richland, WA 99354, USA*

[2]*Atmospheric Science and Global Change Division, Pacific Northwest National Laboratory, Richland, WA 99354, USA*

[3]*State Key Laboratory of Marine Environmental Science, College of Ocean and Earth Science, Xiamen University, Xiamen 361102, China*

[4]*Chemical Sciences Division, Lawrence Berkeley National Laboratory, Berkeley, CA 94720, USA*

[5]*Department of Chemistry, University of California, Berkeley, California 94720, USA.*

[6]*Max Planck Institute for Chemistry, Mainz, Germany*

[7]*Universidade Federal de Sao Paulo, Diadema, SP, Brazil*

[8]*Laboratory for Meteorological Physics, University Clermont Auvergne, Clermont-Ferrand, France*

[9]*Geosciences Institute, Federal University of Para, Belem, 66075-110, Brazil*

[10] *Institute of Physics, University of São Paulo, Rua do Matão 1371, CEP 05508-090, São Paulo, S.P.,Brazil*

[11]*Department of Chemistry, Purdue University, West Lafayette, IN 47907, USA*

[‖]*present address: Physikalisches Institut, Universität Würzburg, Am Hubland, 97074 Würzburg, Germany*

Salt containing aerosols play important roles in atmospheric chemistry and cloud formation, hence understanding and observationally constraining their sources and processes are vital. In the Amazon basin, particles containing mixed sodium salts are routinely observed and attributed to marine aerosols transported from the Atlantic Ocean. Here, using detailed chemical imaging analysis, we show that fungal spores emitted by the forest biosphere (Primary Biogenic Aerosol Particles) contribute to at least 30% (by number) of sodium salt particles observed in the central Amazon basin during the wet season. Hydration experiments, using environmental microreactors complemented with micro-spectroscopy analysis, show that sodium-containing fungal spores have higher hygroscopic growth than sodium-free spores and their sodium content determines their growth factors. Modeling results suggest that fungal spores account for ~69% (31-95%) of the total sodium mass during the wet season and that their fractional contribution increases during nighttime. In contrast to the common assumption that sodium-containing aerosols are primarily arise from marine sources, our results suggest that locally-emitted fungal spores containing sodium contribute substantially to the number and mass of coarse particles containing Na and Cl (similar to sea salt). Hence, their role in cloud formation and contribution to salt cycles and the terrestrial ecosystem in the Amazon basin warrant further consideration.

The Amazon rainforest is often termed the “green ocean” because its relatively pristine atmospheric conditions resemble those in remote marine regions interns of the low particle count and cloud characteristics[1-3]. Specifically, the size distribution and concentration of coarse mode particles in Amazonia are similar to concentrations of coarse mode particles in marine air above the ocean under calm wind conditions of 1-10 m s^{-1} (Supplementary Fig. 1). During the wet season in the Amazon basin, marine aerosol from the Atlantic Ocean is considered the dominant source of coarse mode particles[1,4]. Previous studies assumed the chemical composition of coarse mode aerosols was strongly impacted by long-range transport of soil dust and marine aerosol advected into the central Amazon by large-scale tropospheric circulation that adds to the coarse mode primary biological aerosol particles[5]. However, local sources of sodium salt-containing coarse particles and their potential impact have not been considered and are the focus of this study. This study shows locally emitted fungal spores of coarse super-micrometer size contribute considerably to sodium salt particles in the Amazon basin during wet season. While previous studies reported non-marine sources of sodium salts in fine particles, sodium-containing coarse particles have been attributed typically to sea-salt. Specifically, Ooki et al[6] observed the existence of anthropogenic sodium in fine mode (<1.1 µm) aerosol particles in urban air. Ooki et al[6] suggested potassium as a tracer for anthropogenic sodium in submicron size (0.43µm) particles. Mamane[7] found presence of sodium and potassium in <1 µm particles from waste incinerators emissions. Another study conducted in the metropolitan area of São Paulo found K/Na ratio of 1.4 and 0.9 for coarse (>2.5 µm) and fine (<2.5µm) particles respectively and they attributed this particles to waste incineration and vehicles[8].

Primary biological aerosol particles (PBAP), i.e. particles emitted directly from the biosphere, include fungal spores, pollen, bacteria, algae, protozoa, and fragments of plants and living or dead organisms. Total global emissions of fungal spores are highly uncertain[9-11], estimates vary from 8 Tg per yr^{-1} to 186 Tg yr^{-1}, to as large as 1000 Tg yr^{-1}. Fungal species actively discharge their spores via liquid jets into the air[12,13], a process occurring preferentially under humid atmospheric conditions[13]. Fungal spores and fragments are the most abundant classes of biological particles in the Amazon basin. They contribute up to 25% during daytime (and 45% during nighttime) to the total number of coarse mode particles (1 to 10 µm diameter)[1].

Biological particles, as well as fluorescent particles (inferred to be biological) have been detected in the free troposphere[14,15]. Their chemical compositions are highly variable and, due to the inherent challenges involved in analytically distinguishing between biological and other carbonaceous particles, their exact origins remain insufficiently characterized[1,11]. In Amazonia, concentrations of fluorescent biological particles during the wet season of $\sim 7.3\times 10^{-4}$ m^{-3} are reported, with the highest concentrations occurring during nighttime[16].

Once in the troposphere, biological particles can act as cloud condensation nuclei and ice nuclei, thus impacting warm and cold cloud formation and evolution[11,17]. During their airborne lifetime, Amazonian particles typically experience several cycles of cloud processing[1], which alters their physio-chemical properties and further thwarts their identification. In particular, biological spores rupture at high humidity due to osmotic pressure and subsequently release submicron-sized fragments[18,19]. These fragments contain inorganic salts with the same elements (e.g., Na, Mg, K, Cl) as sea salt. While fragmentation reduces the fraction of original supermicron-

sized biological particles, it increases their contribution to the number concentration of submicron particles. Hence, fragmentation due to atmospheric processing further complicates estimation of their number and size distributions in the accumulation mode[18].

Samples of airborne biological particles were collected during the beginning of the wet season (January and February, 2015) at the ZF2 tower, a pristine rainforest site in Central Amazonia located 40 km North of Manaus[4]. We applied multimodal chemical imaging techniques, such as scanning electron microscopy (SEM) coupled with energy dispersive X-ray (EDX) microanalysis, scanning transmission X-ray microscopy with near-edge X-ray absorption fine structure analysis (STXM/NEXAFS), and nano secondary ion mass spectrometry (NanoSIMS) to analyze size and composition of the biological particles. In situ exposure to water vapor in environmental SEM and STXM studies examined their hygroscopic growth. Finally, model simulations examined the potential contributions of spores in the Amazon basin atmosphere.

Results

Chemical imaging and micro-spectroscopy analysis of fungal spores. SEM images of representative coarse mode particles collected in the Amazon basin are illustrated in Fig 1a. X-ray microanalysis using computer-controlled (CC) SEM/EDX reveals that almost half (~48%) of the coarse mode particles (1.0-3.2 µm aerodynamic diameter) are Na-rich (Fig. 1b). Dust (containing Al, Si, Fe), or internally mixed biological-dust particles (C, N, P, Na, K, Al, Si, and Fe) are second most abundant. Coarse mode sulfate and purely carbonaceous particles are 5% and 3%, respectively. Overall, no considerable differences in the size distributions of different particle classes were observed during night or day or within, above, or below the canopy (Figs. 1c-f). X-

ray microanalysis indicates that the spores are comprised primarily of C, N, and O (carbonaceous in nature) with substantial amounts of other elements e.g., Na, Mg, P, S, Cl, and K (Figs. 2a-c), consistent with previous reports[18,20,21].

The fraction of fungal spores was quantified based on their unique characteristic morphologies (spherical, rod-like or spheroidal in shape), size (1-6 µm), and chemical composition (mostly carbonaceous and containing phosphorous) by electron microscopy imaging and X-ray microanalysis of over 3500 individual particles. Details of the fungal spore identification method are provided elsewhere[18]. The particle-type classification scheme is summarized in Supplementary Fig. 2. During sampling, the number fraction of biological particles was higher below the canopy (60%±2%) than above the canopy (38%±2%) and higher during night (52%±2%) than day (37%±2%). The coarse mode mass fractions of phosphorous and potassium, primarily derived from biological particles[13,22], were larger below than above the canopy and larger during night than day[23]. The higher fraction of biological particles below the canopy is consistent with a local source, and with observations from other tropical rainforests such as Borneo, Malaysia[24]. The increased nighttime number fraction of spores could result from enhanced nighttime active wet discharge of ascospores and basidiospores associated with elevated humidity[13]. Our observation of higher fractions of biological particles during nighttime below the canopy is consistent with previous studies in the Amazon rainforest[16,23,25].

Chemical imaging of the biological particles using X-ray absorption micro-spectroscopy confirms the presence of sodium. Figs. 2d-f show representative STXM images of a biological particle at the Na pre edge (1070 eV) and peak (1079 eV) as well as optical density map. The

sodium atomic weight fraction is size dependent; smaller particles contain a higher fraction of sodium than larger particles (Supplementary Fig. 3). The frequent occurrence of substantial sodium and chlorine masses in biological spores is remarkable. Since biological spores have never been considered as sources of sodium and chlorine, common analytical methods could mistakenly assign them as transported sea salt.

NanoSIMS analysis of fungal spores confirms the presence of sodium-containing particles (Figs. 2g-i) and provides spatial distributions of sodium and other elements within individual particles[26]. For example, the particle in Fig. 2g shows a stronger sodium signal around the particle boundary. That in Fig. 2h displays complex morphology and heterogeneity in its sodium spatial distribution, while the distribution of sodium ions in the elongated fungal spore in Fig. 2i appears homogeneous. These images suggest that sodium distributions within individual fungal spores is diverse, presumably depends upon spore type, and may be influenced by the particle's origin and aging history.

X-ray microanalysis indicates that the average percentage of total spores containing sodium (minimum detection of 3 wt %) is higher above the canopy (daytime: 60%±6%; nighttime: 39%±3%) than below the canopy (daytime: 46%±4%; nighttime: 22%±2%). These differences may partially be influenced by changes in physio-chemical properties of spores that transform during atmospheric processing. For example, when exposed to high humidity conditions, spores rupture and release submicrometer-to-micrometer size fragments. A major fraction (~40-60%) of these fragments contains Na and Cl, and appears morphologically similar to dry sea salt particles

(Supplementary Fig. 4). Subsequently, when exposed to high humidity these hygroscopic fragments grow and become supermicron[18].

Hygroscopicity of fungal spores. We investigated the hygroscopicity of spores using microreactors in environmental SEM[18] and in-situ STXM[27]. At 94% relative humidity (*RH)* sodium-containing fungal spores had area growth factors of 2.4 versus 1.1 if they were sodium-free (Supplementary Figs. 5 and 6). Similarly, mass growth factors of 3.6 and 1.5 were determined for sodium-containing and sodium-free fungal spores, respectively, at 96% *RH*. Remarkably, under high humidity conditions (*RH*~94%) the growth factors of sodium-containing biological particles and NaCl particles are strikingly similar. Using standard analytical methods or inspecting their morphology[18], these processed particles would not readily be recognized as spores. Due to the wide range of sources relevant to biological particle emissions which is further complicated by fragmentation during atmospheric processing, detecting and apportioning aged spores is a daunting task. Hence, microscopy methods underestimate the contribution of biological particles to the total atmospheric aerosol. As a result, the fractions of sodium-containing spores reported here represent lower limits for sodium-bearing fungal spores.

Presence of sodium in fungal spores. Previous studies highlighted the presence of biogenic potassium-rich particles in the Amazonia in the accumulation mode (0.1-1μm diameter)[22]. However, biological particles have never been indicated as a significant source of sodium-containing particles in the Amazonia. This is despite the fact that sodium in biological particles has been reported previously.[20,28] For example, laser-induced breakdown spectroscopy measurements showed the presence of sodium in fungal spores[28] and an X-ray microanalysis study

observed sodium in particles of biogenic origin[29]. The presence of sodium is common in so called halophilic fungi which cannot grow without NaCl[30]. Active discharge, uptake, and efflux processes are likely responsible for the sodium content in the spores and sodium content may vary with different classes and genera of fungi. During active discharge, fungi forcibly eject spores into the atmosphere, together with osmotic fluid containing hexoses, mannitol, phosphate, sodium, and potassium[22,31]. Fungi require K^+ for electrical and osmotic equilibria of the cells and presence of K^+ in several fungal spores is well understood[32]. Previous studies suggested that K^+ can be partially replaced by Na^+ and Na^+ can enhance the growth of fungi[33] and plants[34] under K^+ deficiency conditions. The growth of fungi and uptake of Na^+ varies with their physiological conditions and uptake rates depend on the species and their genes[35]. For example, specific genes (e.g., acu1 and acu2) are responsible for high-affinity Na^+ uptake of Ustilago maydis. When fungi contain excess Na^+, they activate Na^+-efflux ATPase (Adenosinetriphosphatase), which acts as a key enzyme for the biological evolution of fungi[36]. We suggest that uptake and efflux of Na^+ varies with different classes and genera of fungal community. For example, previous studies in Amazonia shows several genera (e.g., Agaricus; Amanita; Aspergillus; Boletus; Cladonia; Mortierella; Puccinia; Lepsita; and Rhizopus) within one class of fungi (Lecanonomycetes)[37,38]. Furthermore, the transpiration of plants[39] and nutrient uptake[40] can also influence the sodium content of spores. Further studies are needed to better comprehend the sodium content in fungal spores by linking chemical composition, molecular biology and diversity of fungal spores.

In contrast to previous reports[1,5], our analyses suggest that locally-emitted biological particles contribute substantially to the total concentration of sodium salt particles in the Amazon basin during the wet season. Backward trajectories calculated with NOAA's HYSPLIT model

suggest that marine air masses would have traveled ~1500 km over 2.5-3 days to reach central Amazonia (Supplementary Fig. 7). The rain records along backward trajectories also indicate that multiple precipitation events occurred during transport, which would likely wash out most of the sea salt particles before their arrival at the sampling site. Hence, these findings support the dominance of locally-emitted spores in the Amazon basin during this study. Furthermore, the presence of a substantial amount (48%±3%) of coarse mode sodium-rich particles below the ~20 m thick forest canopy suggests that local biological emissions contribute significantly to the concentration of sodium-salt particles in the Amazonia.

Model estimate of contribution of fungal spores to sodium budget. To evaluate the geographic distribution and frequency of high fungal spores contributions to airborne sodium mass over the Amazon basin, we conducted model simulations in the Community Earth System Model (CESM1.2.2, see Methods). Briefly, atmospheric emissions and transport simulations were performed using nudging[41] to recreate the observed meteorology of 2014-2015. Particulate sodium content was estimated by considering 70% of fungal spores to be sodium-rich (represents an upper bound), with sodium-rich fungal spores containing 13% sodium by mass, and 30% sodium in sea salt[42](see Methods). We also performed a sensitivity analysis by assuming either 30% or 50% of fungal spores to be sodium-rich.

Model-estimated and observed fungal spore concentrations in various ecosystems and geographic locations[9] (Supplementary Fig. 8) are compared in Fig. 3a. Estimated concentrations are typically higher than observed values but at the measurement site, model-estimated fungal spore concentrations are similar to the observed concentrations. Comparison between model-

estimated and measured sodium mass concentrations from sea salt emissions from various geographical locations at the coastal and island sites (Supplementary Fig. 9) are illustrated in Supplementary Fig. 10. The model estimates of sodium mass concentrations are approximately a factor of two higher than observations at many of the measurement sites; the measurements used in this analysis were all collected at coastal and island locations in the Southern hemisphere, between 20 and 70 S. It is interesting to note that measurements at the remote sites (e.g., Antarctica) are in good agreement with the estimated mass. The time series of both simulated fungal spore and sea salt concentrations (Supplementary Fig. 11) exhibited strong variability. Simulated sea salt concentrations, driven by the long-range transport of marine air, are higher during the dry season compared to the wet season. This may be due to stronger wet removal processes hindering long-range transport, and changes in wind patterns during the wet season.

In the model, during periods of low sea salt aerosol concentration, fungal spores typically contribute the majority of the estimated sodium mass. These conditions occur more frequently during the wet season (Fig. 3b) than the dry season (Supplementary Fig. 12). On average, fungal spores are estimated to contribute 69% of sodium during the wet season. We calculate approximate upper and lower bounds for the fungal spore contribution to the total sodium mass by varying fungal spore mass concentrations by a factor of ten and sea salt mass concentrations by a factor of two. On average, fungal spores are estimated to contribute 69% (31-95%) of sodium during the wet season. However, the level of confidence in fungal spore estimates is arguably higher for the Amazon, since the emissions parameterization was originally developed based primarily on measurements from the Amazon and other tropical rainforests, and simulated fungal spore number concentrations agree with measurements at the Amazonia site within a factor of two. Assuming a

factor of two uncertainty bounds for fungal spore concentrations, their estimated contribution to sodium mass would range between 51-84%. We performed a sensitivity analysis by assuming either 30% or 50% of fungal spores to be sodium-rich. When applying these different assumptions to the model simulations, fungal spores account for 58% (21-90%) or 65% (27-93%) of the total simulated sodium mass during the wet season (Supplementary Fig. 13). The model suggests that the fractional contribution of fungal spores to sodium will increase during nighttime and decrease during daytime (Fig. 3b). This may be the combined result of diurnal cycles in emissions, boundary-layer dynamics, and removal processes (e.g., precipitation). Fig. 3c presents a regional map of the percentage of days when fungal spores contribute at least 50% of estimated total sodium during the wet season.

Discussion

Overall, fungal spores dominated the number fraction of coarse mode in the Amazonia during nighttime and in below-canopy samples, and half of the coarse mode particles are sodium-rich. Previously, sodium-rich particles have been solely attributed to marine aerosol from the Atlantic Ocean. Previous studies in different atmospheric environment show the presence of sodium in fine mode and suggest potassium as a tracer for anthropogenic sodium[6-8,43,44]. Observations of our study are fundamentally different. First, our study focuses on the coarse mode particles. Second, the potassium observed in our study mostly originates from biological particles[22] confirmed by their unique and distinguishable morphology. Those particles were especially abundant during the wet season when biomass burning is not a dominant source of particles in the basin. Remarkably, our experimental and modeling results demonstrate that fungal spores emitted from the rainforest biosphere contribute a major fraction to the sodium budget during wet season, while most of

marine aerosol particles would be removed by wet deposition during transport. Fig. 4 illustrates the sources and atmospheric processing of fungal spores. Hygroscopicity experiments indicate that during cloud processing or high-humidity spores rupture and release submicrometer-to-micrometer size salt fragments[18], further contributing to the sodium budget in accumulation mode particles. This work highlights the potential importance of biological particles as a source of sodium-salt particles in widespread tropical forests, and motivates the necessity of studies investigating the chemistry of biological particles in other forested regions of the globe.

Acknowledgements

This research was supported by the Office of Science of the U.S. Department of Energy (DOE) as part of the Atmospheric System Research program and by the Environmental Molecular Sciences Laboratory (EMSL), a national scientific user facility sponsored by the DOE's Office of Biological and Environmental Research at Pacific Northwest National Laboratory (PNNL). The Pacific Northwest National Laboratory (PNNL) group acknowledges additional support from the Chemical Imaging Initiative of the Laboratory Directed Research and Development program at PNNL. The CCSEM/EDX, ESEM and NanoSIMS analyses, and the Community Earth System Model (CESM) simulations were performed at EMSL/PNNL. PNNL is operated for the U.S. DOE by Battelle Memorial Institute under contract DE-AC06-76RL0. STXM/NEXAFS analysis was performed at beamline 11.0.2.2 of the Advanced Light Source at Lawrence Berkeley National Laboratory. The beamline is supported by the Director, Office of Science, Office of Basic Energy Sciences of the U.S. DOE under Contract No. DE-AC02- 05CH11231. Office of Basic Energy Sciences Division of Chemical Sciences, Geosciences, and Biosciences by the Condensed Phase and Interfacial Molecular Sciences Program of the U.S. DOE are acknowledged for additional

support of the beamline. J. W. and T. H. acknowledge the student exchange program between the University of Würzburg and U.C. Berkeley (curator Professor A. Forchel, Würzburg and NSF IGERT program at UCB, DGE-0333455, Nanoscale Science and Engineering - From Building Blocks to Functional Systems). The Brazilian group acknowledges support from the FAPESP projects 08/58100-2, 2013/25058-1 and 2013/05014-0. We thank Simone R. da Silva and the LBA central office at the Instituto Nacional de Pesquisas da Amazônia (INPA) for assistance and logistical support during sampling at the field site. The CESM project is supported by the National Science Foundation and the Office of Science (BER) of the U.S. Department of Energy. We thank Prof. J. Prospero (U. Miami) and the AEROCOM project for collecting and making available the in situ aerosol measurement network dataset. The authors gratefully acknowledge the NOAA Air Resources Laboratory (ARL) for the provision of the HYSPLIT transport and dispersion model and READY website (http://www.ready.noaa.gov) used in this publication.

Author contributions

S.C., S.B., M.K.G., P.A., and A.L. designed the study. S.C., B.W., T.H., J.W., J.C., A.L., and M.K.G. performed the micro-spectroscopy experiments and analysis. A.L. and G.G.C. collected the samples. S.B., and P.M., performed the modeling work. M.T. gathered observed spore counts data. L.V.R performed backward trajectory analysis. L.V.R., and J.B. analyzed size distribution data. S.C. led the manuscript preparation with contributions from all co-authors.

Competing financial interests

The authors declare no competing financial interests.

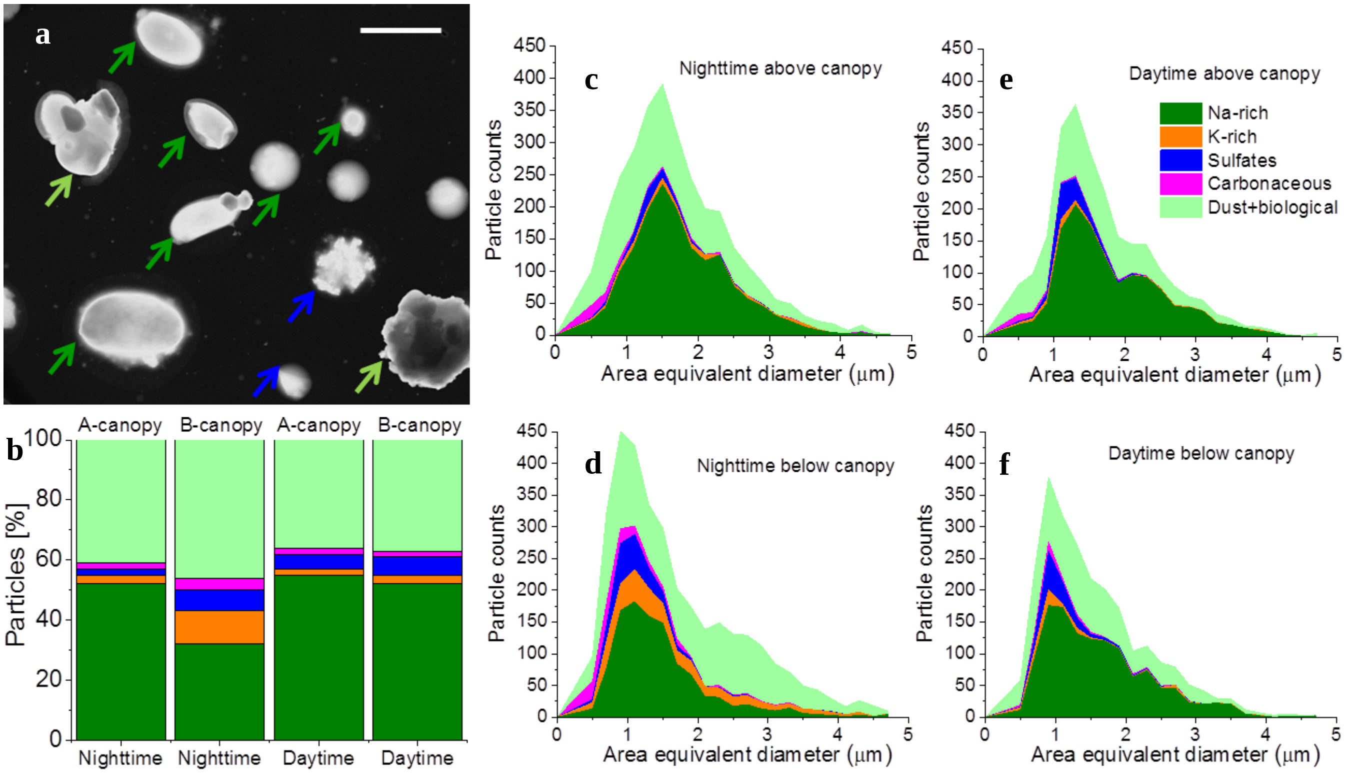

Figure 1| Size distribution and particle-type classes. a, Representative SEM image (forward scattered transmistted electron imaging mode) of particles collected at the ZF2 site in Amazonia. Dark green arrows indicate Na-rich particles, blue arrow indicates a mixed sulfate and Na containing particle with minor contributions of Na, and light green color arrows indicate other types of particles, mostly internally mixed dust and biological particles. Scale bar is 5 μm. **b**, Nightime and daytime number fraction of different particle classes for particles collected above canopy (A-canopy) and below canopy (B-canopy). **c-f** Size distribution of different particle classes.

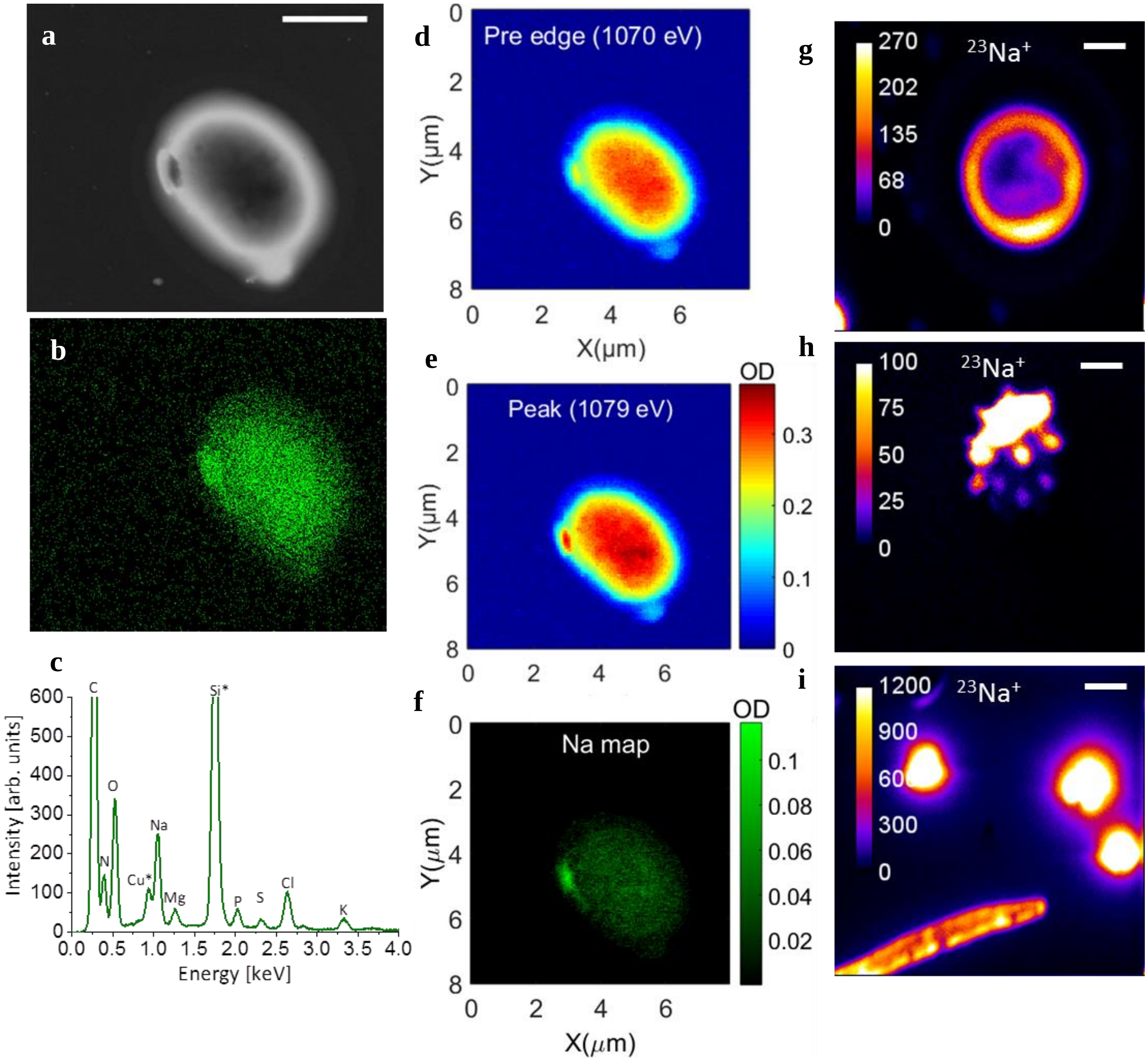

Figure 2| Chemical imaging of Na-containing biological particles. a, SEM image **b**, elemental Na map **c**, EDX spectra of the biological particle. The Cu and Si in the EDX spectra are background peaks originating from the substrate and various instrument parts inside the SEM chamber. STXM images of the same biological particle: **d**, pre-edge (1070 eV), **e**, Na peak (1079 eV) and **f**, Na optical density map. **g-i**, Representative NanoSIMS images of $^{23}Na^{+}$ of selected biological particles showing various distributions of sodium within particles contours. Scale bars are 2 μm.

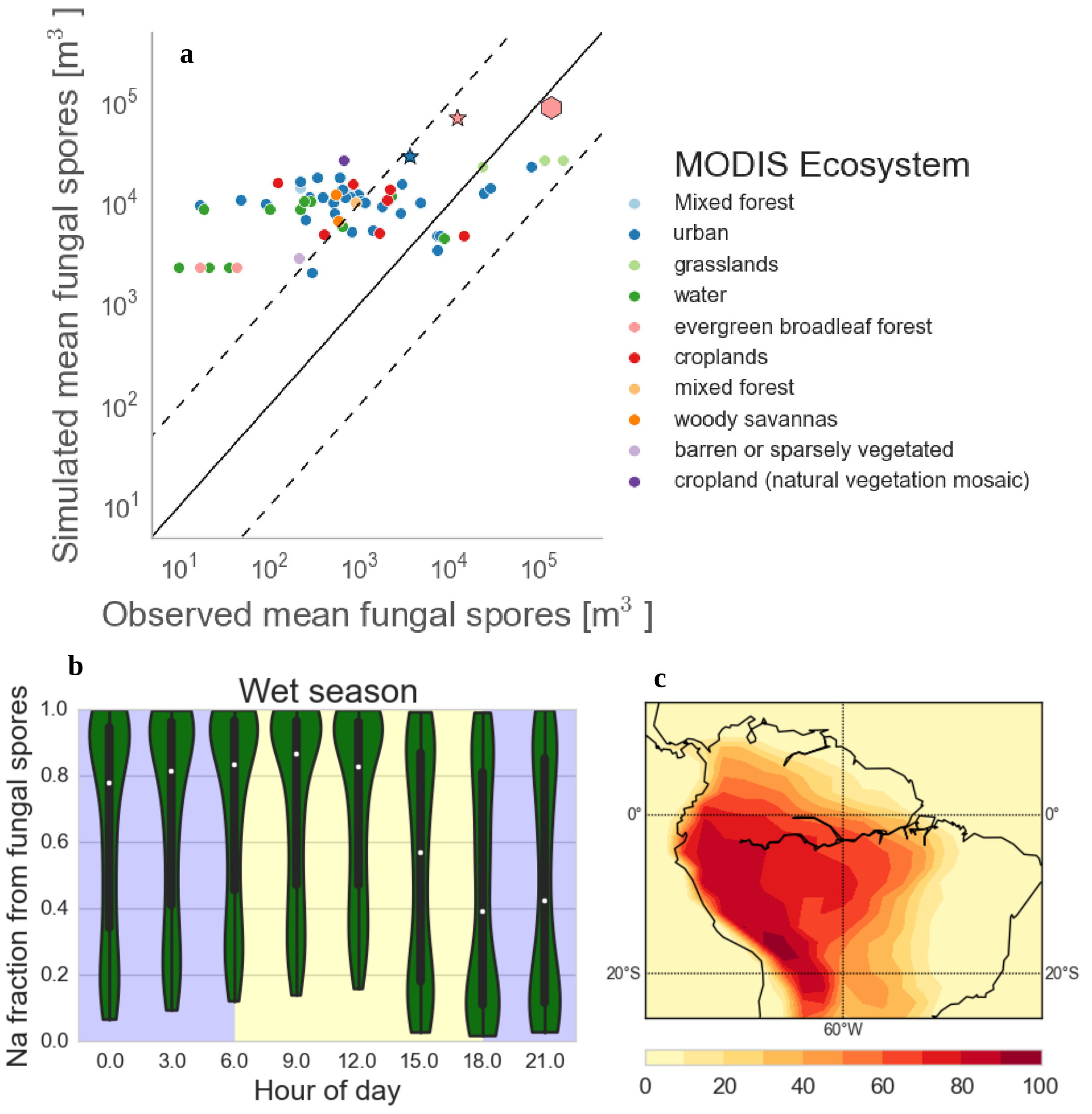

Figure 3| Simulation of sodium contribution from biological particles to total sodium budget in the Amazon area. a, Comparison of model-simulated and observed mean fungal spore concentration at various ecosystem and geographical locations. All model-simulated values are annual means; while experimental observations are mostly mean values over periods from 2 months up to 3 years, as previously reported in the literature (full reference list is provided in Supplementary Table 2). Dotted lines represent an over-or under-estimate of a factor of ten (10:1

and 1:10). The pink hexagonal point represents the measurement campaign (mean simulated fungal spore concentration, Jan 26 – Feb 8, 2015, compared with observed fungal spore concentrations during the same time period). The stars indicate points from literature measurements from tropical rainforests. **b,** Sodium fraction from fungal spores in wet and dry season at the model's nearest grid point to the ZF2 tower, located at 2.84° S 60.0° W, in the model's lowest (near-surface) layer. White dots represent the median, thick lines represent 25-75 percentile, and thin vertical lines represent the range of minimum and maximum value; violin plot represents a kernel density estimate distribution of the sodium fraction values. Background shading indicates time of day, blue represents night [1800-0600] and yellow represents day [0600-1800]. **c,** Percentage of days when fungal spores contributed at least 50% of estimated total sodium in wet season, assuming that 70% of fungal spores are sodium-rich, containing 13% sodium by mass, and sea salt aerosol is composed of 30% Na by mass. Results for dry season are shown in Supplementary Fig. 12.

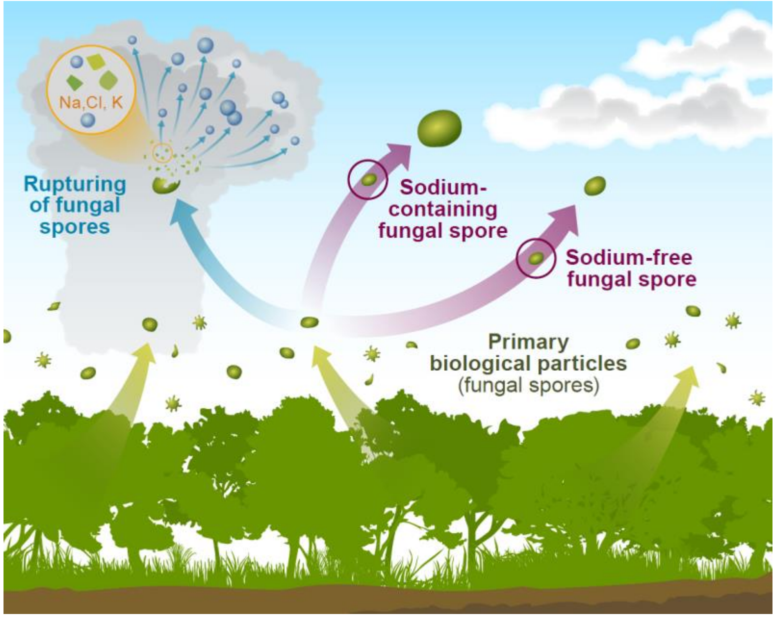

Figure 4| Sources and atmospheric processing of fungal spore particles in the Amazon rainforest. Sodium-containing and sodium-free fungal spore particles are emitted from the Amazon rainforest. Sodium-containing fungal spores exhibit higher hygroscopic growth compared to sodium-free fungal spores. When they are exposed to high humidity conditions, or through cloud processing, fungal spore particles rupture and release submicrometer-to-micrometer size fragments. A substantial fraction of the fragments which contains Na, Cl, and K, and appear morphologically similar to dry sea salt particles. These hygroscopic salt fragments can participate in cloud formation.

Data Availability

All relevant data are available from the authors.

References

1. Martin, S. T. *et al.* Sources and properties of Amazonian aerosol particles. *Reviews of Geophysics* **48**, RG2002, doi:10.1029/2008RG000280 (2010).
2. Roberts, G. C., Andreae, M. O., Zhou, J. & Artaxo, P. Cloud condensation nuclei in the Amazon Basin:"Marine" conditions over a continent? *Geophysical research letters* **28**, 2807-2810 (2001).
3. Williams, E. *et al.* Contrasting convective regimes over the Amazon: Implications for cloud electrification. *Journal of Geophysical Research: Atmospheres* **107**, 8082, doi:10.1029/2001JD000380 (2002).
4. Artaxo, P. *et al.* Atmospheric aerosols in Amazonia and land use change: from natural biogenic to biomass burning conditions. *Faraday discussions* **165**, 203-235 (2013).
5. Talbot, R. W. *et al.* Aerosol chemistry during the wet season in central Amazonia: The influence of long-range transport. *Journal of Geophysical Research: Atmospheres* **95**, 16955-16969, doi:10.1029/JD095iD10p16955 (1990).
6. Ooki, A., Uematsu, M., Miura, K. & Nakae, S. Sources of sodium in atmospheric fine particles. *Atmospheric Environment* **36**, 4367-4374, doi:https://doi.org/10.1016/S1352-2310(02)00341-2 (2002).
7. Mamane, Y. Estimate of municipal refuse incinerator contribution to philadelphia aerosol-I. Source analysis. *Atmospheric Environment (1967)* **22**, 2411-2418, doi:https://doi.org/10.1016/0004-6981(88)90473-8 (1988).
8. Bourotte, C. *et al.* Association between ionic composition of fine and coarse aerosol soluble fraction and peak expiratory flow of asthmatic patients in São Paulo city (Brazil). *Atmospheric Environment* **41**, 2036-2048, doi:https://doi.org/10.1016/j.atmosenv.2006.11.004 (2007).
9. Sesartic, A. & Dallafior, T. N. Global fungal spore emissions, review and synthesis of literature data. *Biogeosciences* **8**, 1181-1192, doi:10.5194/bg-8-1181-2011 (2011).
10. Jacobson, M. Z. & Streets, D. G. Influence of future anthropogenic emissions on climate, natural emissions, and air quality. *Journal of Geophysical Research: Atmospheres* **114**, n/a-n/a, doi:10.1029/2008JD011476 (2009).
11. Després, V. R. *et al.* Primary biological aerosol particles in the atmosphere: a review. *Tellus B* **64**, doi:10.3402/tellusb.v64i0.15598 (2012).
12. Pringle, A., Patek, S. N., Fischer, M., Stolze, J. & Money, N. P. The captured launch of a ballistospore. *Mycologia* **97**, 866-871 (2005).
13. Elbert, W., Taylor, P. E., Andreae, M. O. & Pöschl, U. Contribution of fungi to primary biogenic aerosols in the atmosphere: wet and dry discharged spores, carbohydrates, and inorganic ions. *Atmos. Chem. Phys.* **7**, 4569-4588, doi:10.5194/acp-7-4569-2007 (2007).
14. Twohy, C. H. *et al.* Abundance of fluorescent biological aerosol particles at temperatures conducive to the formation of mixed-phase and cirrus clouds. *Atmos. Chem. Phys.* **16**, 8205-8225, doi:10.5194/acp-16-8205-2016 (2016).

15 Griffin, D. W. Terrestrial Microorganisms at an Altitude of 20,000 m in Earth's Atmosphere. *Aerobiologia* **20**, 135-140, doi:10.1023/b:aero.0000032948.84077.12 (2004).

16 Huffman, J. A. *et al.* Size distributions and temporal variations of biological aerosol particles in the Amazon rainforest characterized by microscopy and real-time UV-APS fluorescence techniques during AMAZE-08. *Atmos. Chem. Phys.* **12**, 11997-12019, doi:10.5194/acp-12-11997-2012 (2012).

17 Haga, D. I. *et al.* Ice nucleation by fungal spores from the classes Agaricomycetes, Ustilaginomycetes, and Eurotiomycetes, and the effect on the atmospheric transport of these spores. *Atmos. Chem. Phys.* **14**, 8611-8630, doi:10.5194/acp-14-8611-2014 (2014).

18 China, S. *et al.* Rupturing of Biological Spores As a Source of Secondary Particles in Amazonia. *Environmental Science & Technology* **50**, 12179-12186, doi:10.1021/acs.est.6b02896 (2016).

19 Steiner, A. L. *et al.* Pollen as atmospheric cloud condensation nuclei. *Geophysical Research Letters* (2015).

20 Ebert, M., Weinbruch, S., Hoffmann, P. & Ortner, H. M. The chemical composition and complex refractive index of rural and urban influenced aerosols determined by individual particle analysis. *Atmospheric Environment* **38**, 6531-6545, doi:http://dx.doi.org/10.1016/j.atmosenv.2004.08.048 (2004).

21 Zhisheng, Z. *et al.* Significant influence of fungi on coarse carbonaceous and potassium aerosols in a tropical rainforest. *Environmental Research Letters* **10**, 034015 (2015).

22 Pöhlker, C. *et al.* Biogenic potassium salt particles as seeds for secondary organic aerosol in the Amazon. *Science* **337**, 1075-1078 (2012).

23 Guyon, P. *et al.* In-canopy gradients, composition, sources, and optical properties of aerosol over the Amazon forest. *Journal of Geophysical Research: Atmospheres* **108**, 4591, doi:10.1029/2003JD003465 (2003).

24 Gabey, A. M. *et al.* Measurements and comparison of primary biological aerosol above and below a tropical forest canopy using a dual channel fluorescence spectrometer. *Atmos. Chem. Phys.* **10**, 4453-4466, doi:10.5194/acp-10-4453-2010 (2010).

25 Rizzo, L. V., Artaxo, P., Karl, T., Guenther, A. B. & Greenberg, J. Aerosol properties, in-canopy gradients, turbulent fluxes and VOC concentrations at a pristine forest site in Amazonia. *Atmospheric Environment* **44**, 503-511, doi:https://doi.org/10.1016/j.atmosenv.2009.11.002 (2010).

26 Boxer, S. G., Kraft, M. L. & Weber, P. K. Advances in imaging secondary ion mass spectrometry for biological samples. *Annual review of biophysics* **38**, 53-74 (2009).

27 Piens, D. S. *et al.* Measuring Mass-Based Hygroscopicity of Atmospheric Particles through in Situ Imaging. *Environmental Science & Technology* **50**, 5172-5180, doi:10.1021/acs.est.6b00793 (2016).

28 Hybl, J. D., Lithgow, G. A. & Buckley, S. G. Laser-induced breakdown spectroscopy detection and classification of biological aerosols. *Appl. Spectrosc.* **57**, 1207-1215 (2003).

29 Krejci, R., Ström, J., de Reus, M. & Sahle, W. Single particle analysis of the accumulation mode aerosol over the northeast Amazonian tropical rain forest, Surinam, South America. *Atmos. Chem. Phys.* **5**, 3331-3344, doi:10.5194/acp-5-3331-2005 (2005).

30 Plemenitaš, A. *et al.* Adaptation to high salt concentrations in halotolerant/halophilic fungi: a molecular perspective. *Frontiers in Microbiology* **5**, doi:10.3389/fmicb.2014.00199 (2014).

31 Webster, J. *et al.* Mannitol and hexoses are components of Buller's drop. *Mycological Research* **99**, 833-838, doi:http://dx.doi.org/10.1016/S0953-7562(09)80737-5 (1995).

32 Rodríguez-Navarro, A. Potassium transport in fungi and plants. *Biochimica et Biophysica Acta (BBA) - Reviews on Biomembranes* **1469**, 1-30, doi:https://doi.org/10.1016/S0304-4157(99)00013-1 (2000).

33 Camacho, M., Ramos, J. & Rodríguez-Navarro, A. Potassium requirements ofSaccharomyces cerevisiae. *Current Microbiology* **6**, 295-299, doi:10.1007/bf01566880 (1981).

34 Subbarao, G. V., Wheeler, R. M., Stutte, G. W. & Levine, L. H. How far can sodium substitute for potassium in red beet? *Journal of Plant Nutrition* **22**, 1745-1761, doi:10.1080/01904169909365751 (1999).

35 Benito, B., Garciadeblás, B., Schreier, P. & Rodríguez-Navarro, A. Novel P-type ATPases mediate high-affinity potassium or sodium uptake in fungi. *Eukaryotic Cell* **3**, 359-368 (2004).

36 Benito, B., Garciadeblás, B. & Rodríguez-Navarro, A. Potassium- or sodium-efflux ATPase, a key enzyme in the evolution of fungi. *Microbiology* **148**, 933-941, doi:doi:10.1099/00221287-148-4-933 (2002).

37 Womack, A. M. *et al.* Characterization of active and total fungal communities in the atmosphere over the Amazon rainforest. *Biogeosciences* **12**, 6337-6349, doi:10.5194/bg-12-6337-2015 (2015).

38 Fröhlich-Nowoisky, J. *et al.* Biogeography in the air: fungal diversity over land and oceans. *Biogeosciences* **9**, 1125-1136, doi:10.5194/bg-9-1125-2012 (2012).

39 Nemeryuk, G. Migration of salts into the atmosphere during transpiration. *Soviet Plant Physiol* (1970).

40 Lawson, I. T., Jones, T. D., Kelly, T. J., Coronado, E. N. H. & Roucoux, K. H. The geochemistry of Amazonian peats. *Wetlands* **34**, 905-915 (2014).

41 Zhang, K. *et al.* Technical Note: On the use of nudging for aerosol–climate model intercomparison studies. *Atmos. Chem. Phys.* **14**, 8631-8645, doi:10.5194/acp-14-8631-2014 (2014).

42 Pilson, M. An Introduction to the Chemistry of the Sea Prentice-Hall. *Inc. Upper Saddle River, NJ. 431p* (1998).

43 Kitto, M. E. Trace-Element Patterns in Fuel Oils and Gasolines for Use in Source Apportionment. *Air & Waste* **43**, 1381-1388, doi:10.1080/1073161X.1993.10467213 (1993).

44 Mizohata, A. & Mamuro, T. Chemical element balances and identification of air pollution sources in Sakai, Osaka (I). *Journal of Japan Society of Air Pollution* **15**, 198-206 (1980).

45 Bauer, H. *et al.* Determination of the carbon content of airborne fungal spores. *Analytical Chemistry* **74**, 91-95 (2002).

46 Moffet, R., AV, T. & Gilles, M. in *Fundamentals and Applications in Aerosol Spectroscopy* (eds R Signorell & JP Reid) 419–462 (Taylor & Francis, 2010).

47 Heald, C. L. & Spracklen, D. V. Atmospheric budget of primary biological aerosol particles from fungal spores. *Geophysical Research Letters* **36**, L09806, doi:10.1029/2009GL037493 (2009).
48 Neale, R. B. *et al.* Description of the NCAR community atmosphere model (CAM 5.0). *NCAR Tech. Note NCAR/TN-486+ STR* (2010).
49 Hurrell, J. W. *et al.* The Community Earth System Model: A Framework for Collaborative Research. *Bulletin of the American Meteorological Society* **94**, 1339-1360, doi:10.1175/bams-d-12-00121.1 (2013).
50 Ma, P.-L. *et al.* The role of circulation features on black carbon transport into the Arctic in the Community Atmosphere Model version 5 (CAM5). *Journal of Geophysical Research: Atmospheres* **118**, 4657-4669, doi:10.1002/jgrd.50411 (2013).
51 Rienecker, M. M. *et al.* MERRA: NASA's Modern-Era Retrospective Analysis for Research and Applications. *Journal of Climate* **24**, 3624-3648, doi:10.1175/jcli-d-11-00015.1 (2011).
52 Liu, X. *et al.* Toward a minimal representation of aerosols in climate models: description and evaluation in the Community Atmosphere Model CAM5. *Geosci. Model Dev.* **5**, 709-739, doi:10.5194/gmd-5-709-2012 (2012).
53 Mårtensson, E. M., Nilsson, E. D., de Leeuw, G., Cohen, L. H. & Hansson, H. C. Laboratory simulations and parameterization of the primary marine aerosol production. *Journal of Geophysical Research: Atmospheres* **108**, 4297, doi:10.1029/2002JD002263 (2003).
54 Monahan, E. C. in *The Role of Air-Sea Exchange in Geochemical Cycling* 129-163 (Springer, 1986).
55 Textor, C. *et al.* Analysis and quantification of the diversities of aerosol life cycles within AeroCom. *Atmos. Chem. Phys.* **6**, 1777-1813, doi:10.5194/acp-6-1777-2006 (2006).
56 Pope, F. D. Pollen grains are efficient cloud condensation nuclei. *Environmental Research Letters* **5**, 044015 (2010).
57 Pöhlker, M. L. *et al.* Long-term observations of cloud condensation nuclei in the Amazon rain forest – Part 1: Aerosol size distribution, hygroscopicity, and new model parametrizations for CCN prediction. *Atmos. Chem. Phys.* **16**, 15709-15740, doi:10.5194/acp-16-15709-2016 (2016).

Methods

Sampling Site and Sample Collection. Samples were collected during the wet season (January and February, 2015) at the ZF2 Tower (02°35.3517′ S, 60 06.8333′ W), a pristine rainforest site in Central Amazonia located 40 km North of Manaus margins. Samples were collected through a sampling inlet located at a height of 2 meters (below canopy) and 39 m above ground (above canopy), during day and night with 30 L/min sampling rate. Particles were collected onto 400 mesh

transmission electron microscopy (TEM) grids coated with Carbon Type-B films (Ted Pella, Inc.), silicon nitride membrane substrates (0.5 × 0.5 mm^2 Si_3N_4 window size, 100 nm membrane thickness, 5 × 5 mm^2 Si frame size; Silson, Inc.). 10-stage Micro-Orifice Uniform Deposition Impactors™ (MOUDI™; model 110-R, MSP, Inc.) were used for sampling. This study focuses on the samples from stages 4 and 5 (size range: 1.0-3.2 µm) where the relative abundance of biological particles is high. All the experiments were performed with diligently. Samples were handled with caution. For examples, tweezers were cleaned with solvents prior to handling the samples; new gloves were used for handling of samples after each experiments. Examination of blank substrate showed negligible contamination on the sample substrate (Supplementary Figure 14). Investigation of particles from stages 6 and 7 (size range: 0.32-1.0 µm) showed Na-containing particles in stages 6 and 7 are significantly lower (Na-containing particle <15%) than particles on stages 4 and (Supplementary Figure 15). This result suggest that high fraction of sodium-salt particles are indeed from spores that were deposited on stages 4 and 5. Furthermore, sodium content in spores was observed irrespective of different sample preparation and techniques (e.g., SEM/EDX, STXM and NanoSIMS).

Chemical imaging of particles. An environmental scanning electron microscope (ESEM, Quanta 3D model, FEI, Inc.) with an EDAX™ energy dispersive X-ray (EDX) spectrometer and a Si(Li) detector with a 10 mm^2 active area and an ATW2 window was used under vacuum conditions for imaging and X-ray microanalysis. Particles were imaged using secondary electron (SE) and forward scattered transmitted electron (STE) signals. STE imaging mode was used for particle identification and computer-controlled (CC) SEM/EDX analysis. X-ray spectrum for each identified particle was acquired at an acceleration voltage of 20 kV and at a beam current of 430 pA for 10 seconds. The computer-controlled CCSEM/EDX analysis automatically investigates the

specified scanning area and detects particles in the specified fields of view. In this way, we can detect ~90% of the particles deposited on the grid. Particles collected on the edges (~5-10%) of the Cu mesh were excluded from the results based on their high Cu background signal. We analyzed ~80% of the particles that were collected onto TEM B-film grids placed on stages 4 and 5 of the impactor. Area equivalent diameters (AED) were calculated from the 2D projected area recorded for each individual particle. Particle elemental composition was quantified and is reported in units of atomic fractions. The percentage of sodium content in fungal spores was estimated using the known mass fraction of spores[13] and carbon/sodium wt% from X-ray microanalysis. Carbon content in spores ranges from 42-66%, with an average of 51%[45]. The average carbon/sodium wt% in fungal spores is 4:1. The mass of the spores is estimated from the size distribution of microscopy analysis and assuming a density of 1 gm cm^{-3})[13,16].

STXM utilizes a focused soft X-ray beam generated from the synchrotron light source to probe chemical bonding of specific elements of interest within individual particles. STXM images are obtained by raster scanning the sample at fixed photon energy and recording intensities of the transmitted X-rays at each pixel. The optical density $(-\ln(I_{(d)}/I_0)$ is calculated based on the measured intensity (I_d) using Beer-Lambert's law [46]. Transmission intensity through a particle free region of the substrate is used to obtain I_0.

The hygroscopicity of biological particles was investigated using a temperature-controlled cooling stage in the environmental SEM to estimate the area growth factor (ratio of wet-to-dry diameters) and a microreactor for in situ STXM hydration experiments[27] to estimate the mass growth factor (ratio of wet-to-dry oxygen mass).

Nano secondary ion mass spectrometry (NanoSIMS, CAMECA 50L, Gennevilliers Cedex, France) was used to perform imaging of Na+ ions. NanoSIMS provides high lateral resolution. Samples were coated with a thin layer of Au to minimize charging and non-equilibrium sputtering effects during analysis. A 16 keV O^- primary ion beam was used for analysis. Prior to data collection, samples were pre-sputtered with about 10^{16} O^- ions cm^{-2}. Analysis of 15 μm × 15 μm and 256 px × 256 px were performed with a dwell time of 13.5 ms px^{-1}. Beam diameter was estimated to be about 250 nm.

Community Atmosphere Model Description. Fungal spore emissions were calculated according to the global model parameterization of Heald and Spracklen[47]. While this parameterization has substantial uncertainties, it was developed on the basis of observed mannitol concentrations from various continental locations and seasons; about half of these observations were from PM2.5 aerosol collected in the tropical Brazilian rainforest during the wet season, where concentrations were 2-3 times higher than those reported at extratropical locations[13]. Therefore, we believe the parameterization can be used with greater confidence in the Amazonian rainforest, particularly during the wet season, than at other times and locations.

A simulation for present-day conditions was performed using the Community Atmosphere Model (CAM5)[48], which is the atmosphere component of the Community Earth System Model (CESM, version 1.2.1)[49]. The model was configured at a horizontal resolution of 1.9° latitude by 2.5° longitude, with 30 vertical layers ranging from the surface to 2.26 hPa. The simulation was performed from March 2014 to March 2015 with constrained meteorology [50],where model winds are nudged toward the MERRA reanalysis[51] with a relaxation time of 6 hours[41].

The model tracks the emissions and concentrations of six chemical species of aerosol: sea salt, dust, sulfate, secondary organic aerosol (SOA), black carbon (BC) and continental primary organic aerosol (POA). Aerosol transport and microphysics, including nucleation, condensation, and coagulation, is calculated within a modal aerosol module with three size classes (MAM3)[52], including the Aitken mode (dry diameter size range of 0.02–0.08 μm), accumulation mode (0.08–1.0 μm), and coarse mode (1.0–10.0 μm). Sea salt aerosol emission follows Mårtensson et al[53] for small particles (diameter < 2.8 μm), while emissions of larger particles (diameter > 2.8 μm) follow Monahan et al[54]. The simulated sea salt budget was evaluated in Liu et al[52] and falls within the AeroCom values[55]; the modeled global sea salt emission flux is within the range of 2200 ~ 118,000 Tg yr^{-1} using different sea salt source functions. A detailed description of the model's aerosol removal processes can be found elsewhere[48].

Fungal spores were emitted into the model's coarse mode, and are removed by wet and dry deposition in the same manner as other aerosol species. Upon emission both fungal spore mass and coarse mode number are increased, with the mass-to-number ratio for emissions determined by assuming that fungal spores have 4 μm diameter upon emission. The model's coarse mode particle number concentration also includes number contributions from other aerosol species, which are treated as internally mixed within the coarse mode, and the particle diameter is calculated dynamically by the aerosol microphysics routines from the total coarse mode aerosol mass and number, and a fixed geometric width. Fungal spores were assigned the same optical properties as the model's organic carbon tracer, which is close to the observed refractive indices of fungal spores (1.4-0i)[20]. Fungal spores were assigned a material density of 1 g cm^{-3}, which is commonly used in literature[13,16]. Fungal spores were assigned a hygroscopicity parameter (κ) of

0.1, similar to that of pollen grains[56]. The observed overall κ value for ambient below-canopy aerosol in the Amazon is 0.22±0.05 in the accumulation mode, with an overall mean value of κ=0.17±0.06[57].

Reviewers' comments:

Reviewer #1 (Remarks to the Author):

This paper reports that fungal spores are major sources of particle sodium, with size distributions and properties that are similar to that from sea salt particles. This is a new finding that will be of broad interest and certainly justifies publication in Nature Communications. Given the importance of understanding the role of particles in cloud formation and particles, which impacts climate, this will definitely influence thinking in the field.

The paper is very well-written and documented, and this reviewer has essentially no substantiative comments or suggestions to make. A very (!!) minor suggestion is to add to the description of Supplementary Figure 9 the same description as for Figure 3b, since it is an unusual (but effective) way to present data.

Reviewer #2 (Remarks to the Author):

1. The major claim of the paper is that sodium salts present in atmospheric aerosols arise from fungal spores. Fungal spores represent a salt source that has previously not been reported. This is a fascinating source. However, the manuscript's assessment of the importance of this finding is not supported by previous literature or clear explanations. Regarding previous literature, the abstract states, "In sharp contrast with the assumption that sodium-containing aerosols arise solely from marine sources,..." This statement is simply not true. There is a wealth of papers on non-marine sources of sodium salts in aerosols.

2. More problematically, while the observation of salts arises in fungal spores in interesting in its own right, the manuscript in its present form does not provide an adequate explanation or even compelling suggestions for how/when/under what circumstances or via what mechanism the salt in incorporated into some, but not all, spores. If an insightful discussion were included, I would rate this manuscript much more positively. As written, it is lacking.

3. A second finding is that spores which contain salt are more hygroscopic than those which do not. While I'm not aware of any previous results on precisely this topic, it is very well known that salts are deliquescent materials whereas organic macromolecules and other organic compounds found in spores are not. So, this result is very obvious.

A minor point –

4. According to Figure 3, the Na fraction for fungal spores is estimated to be between 0.1 and 1.0 (i.e. nearly any value in the early hours, and later in the day transitions to an even wider spread in

possible fractions. I may be missing something, but I don't find this result to be a significant finding.

5. In summary, the authors have one potentially interesting surprising field observation in the Amazon. However, additional consideration of how this may arise is needed before I could recommend publication.

Reviewer #3 (Remarks to the Author):

China et al. present new multi-instrument observations of the sodium content of fungal spores in the Amazon basin, and use those in combination with backward trajectories and earth system model simulations to suggest that the fungal spores are the main source of sodium containing particles in the wet season.

The major claims of the paper are as follows:

1. Fungal spores contribute significantly to particle-sodium in the Amazon, contributing at least 30% by number of sodium salt containing particles

2. Sodium-containing fungal spores show higher hygroscopic growth than sodium-free spores
3. Fungal spores account for ~62% of sodium mass during the wet season, according to model simulations
4. Therefore, the role of fungal spores in cloud formation and salt cycles in the Amazon deserves further consideration

The claims in the MS are significant and novel. The two closest papers known to me, one from (partly) the same research group, discuss that 1) K-rich bio-aerosols are seeds for SOA formation, but the paper does not mention Na-containing particles (Poehlker et al. 2010) and 2) that when spores rupture at high RH, fungal fragments that contain Na salts are released, but the paper does not quantify Na-content of the particles nor their contribution to the particle Na-budget over Amazonia (China et al. 2016).

The results are of interest to others in the community, since they give 1) new insight into Na-budget of the Amazon from a source that was not considered previously and 2) they add a new line of thought to the debate on sources of cloud-active particles in Amazon, which is a long-standing problem.

The results are of interest to the wider field, because this work adds a new aspect to the understanding of biogeochemical cycles in the Amazon rainforest, and how the forest itself sustains biogeochemical and hydrological recycling. Especially the role of fungal spores therein is one that has not received much attention so far, and this work is likely to start a further debate on this topic.

The MS is clearly structured and generally convincing, but I think it could profit from some further clarifications:

1) Since I am not familiar with the instruments that are used, I cannot judge the quality of the experimental work. However, I think the MS would benefit from a short explanation of the sampling procedure: are all coarse particles identified that were sampled? How is it determined that a particle is a fungal spore and not another kind of PBA (e.g. bacterium or pollen)? Is this done based on size, morphology or otherwise? A brief description could be added, for instance in L107.

2. The estimate of the contribution of spores to the Na-budget, depends on two factors which are very uncertain: local emissions of fungal spores and transport of sea salt aerosol into the Amazon basin. I think the fungal spores emissions are constrained reasonably well by the observations of their concentration, which the model seems to underestimate somewhat. However, if global emission estimates of sea salt span 2 orders of magnitude, how reliable is the estimate of sea salt aerosol transported into the Amazon basin, with no direct observations available for validation? Can the authors provide an estimate of the uncertainty in the sea salt contribution to the particle sodium budget? This could provide a bit more context to the number of 62% of total sodium mass that is now assigned to fungal spores.

Minor comments:

1. Title: Since the main finding is that fungal spores contribute significantly to sodium salt particles, why not mention this in the title, for instance: ‘Fungal spores as source of sodium salt particles in the Amazon basin’

2. L29-30: pls add that this is the case for the wet season

3. In L52-53, you state that marine aerosol is considered the main source of coarse aerosol, whereas in L213 you state that fungal spores dominate the coarse mode. It would be good to clarify what is actually meant by dominate: mass, number? And to briefly include the evidence for the statement in L213.

4. L107: how many particles were sampled and how was it ascertained that they are representative for the wet-season coarse aerosol? And how was a particle identified as a fungal spore? Was every collected particle in the sampling periods in Supp. Table 2 classified?

5. L180: since the back trajectories and rain records are based on reanalysis data, I would not refer to them by ‘observations’ here, but rather by ‘simulations’

6. L190-191: could you clarify here where the percentages of 70 and 13% for sodium rich-spores and spore sodium mass, respectively, are based on?

7. L208-209: how would nighttime emissions or a shallow nocturnal boundary layer explain that the Na fraction from spores drops only after noon, and not in the early morning at the onset of

turbulence? Besides, the violin plots in figure 3 seem to show bimodal distributions for the Na fraction from fungal spores for each time of the day. What is the reason for that?

8. L217: ‘a major fraction to the sodium budget’: please add here that this is for the wet season

9. L533-534: which size distribution was assumed for the spores? Within the range of the model coarse mode (1-10 micron), the assumed size can make large differences for the efficiency of the wet and dry removal

10. Supp. Fig. 9: It seems that the same violin plot is show twice: the plot looks very similar to that in Fig. 3 and the title says ‘wet season’ while the figure is for the dry season. Please include the right plot for the right season.

11. Technical corrections/suggestions:- L32: pls remove ‘we’

12. L50: similar to concentration of marine air -> similar to concentrations of coarse mode particles in marine air

13. L57: has -> have and is -> are

14. L94: modeling studies -> model simulations

15. L94: ‘potential contributions of spores’: contributions to what?

16. Fig. 1, caption: representative is misspelled

17. Supp. Table 1: Samples 3A and 3B have an end time prior to the start time

18. Supp. Fig. 3: C/Na -> Cl/Na

Reply to the Reviewer’s comments (NCOMMS-17-33311-T)

<u>Reviewer #1 (Remarks to the Author):</u>

This paper reports that fungal spores are major sources of particle sodium, with size distributions and properties that are similar to that from sea salt particles. This is a new finding that will be of broad interest and certainly justifies publication in Nature Communications. Given the importance of understanding the role of particles in cloud formation and particles, which impacts climate, this will definitely influence thinking in the field.

The paper is very well-written and documented, and this reviewer has essentially no substantiative comments or suggestions to make. A very (!!) minor suggestion is to add to the description of Supplementary Figure 9 the same description as for Figure 3b, since it is an unusual (but effective) way to present data.

We appreciate the positive response of the reviewer regarding the novelty and importance of the findings reported in the manuscript and for recommending the manuscript for publication. As suggested, we added more detailed description of Supplementary Figure 9 in the caption.

"*White dots represent the median, thick lines represent 25-75 percentile, and thin vertical lines represent the range of minimum-to-maximum values; violin plot represents a kernel density distribution of the sodium fraction values. Background shading indicates time of day, blue represents night [1800-0600] and yellow represents day [0600-1800].*"

Reviewer #2 (Remarks to the Author):

1. The major claim of the paper is that sodium salts present in atmospheric aerosols arise from fungal spores. Fungal spores represent a salt source that has previously not been reported. This is a fascinating source. However, the manuscript's assessment of the importance of this finding is not supported by previous literature or clear explanations. Regarding previous literature, the abstract states, "In sharp contrast with the assumption that sodium-containing aerosols arise solely from marine sources,..." This statement is simply not true. There is a wealth of papers on non-marine sources of sodium salts in aerosols.

We agree that fungal spores are a fascinating source of sodium salt particles in Amazonia. Sodium content in atmospheric aerosol is primarily attributed to sea water, especially the coarse mode particles. However, as the reviewer pointed out, there are also non-marine sources of sodium salts in atmospheric aerosols. For example, Ooki et al[1] observed the existence of anthropogenic sodium in fine mode aerosol particles in urban air. Ooki et al[1] suggested potassium as a tracer for anthropogenic sodium. Mamane[2] found presence of sodium and potassium in submicron particles from waste incinerators emissions. Another study conducted in the metropolitan area of São Paulo by Bourotte et al.[3] found K/Na ratio of 1.4 and 0.9 for coarse and fine particles respectively and they attributed this particles to waste incineration and vehicles. Most of these studies show the presence of sodium in fine mode and suggest potassium as a tracer for anthropogenic sodium. Observations of our study are fundamentally different. First, our study focuses on coarse mode particles. Second, the potassium observed in our study mostly originates from biological particles[4], especially during the wet season when biomass burning is not a dominant source in the basin.

To clarify these details, we modified the sentence and the manuscript (as suggested by Reviewer 3) to reflect that our main finding is that fungal spores contribute to sodium salt particles in the Amazon basin.

"In contrast to the common assumption that sodium-containing aerosols are primarily arise from marine sources, our results suggest that locally-emitted fungal spores containing sodium contribute substantially to the number and mass of coarse particles containing Na and Cl (similar to sea salt)."

We adopted the title proposed by reviewer 3.

"Fungal spores as a source of sodium salt particles in the Amazon basin"

2. More problematically, while the observation of salts arises in fungal spores in interesting in its own right, the manuscript in its present form does not provide an adequate explanation or even compelling suggestions for how/when/under what circumstances or via what mechanism the salt in incorporated into some, but not all, spores. If an insightful discussion were included, I would rate this manuscript much more positively. As written, it is lacking.

In the original manuscript we provided basic information (lines: 167-172) regarding the nature for sodium content in fungal spores. To address the reviewers concerns, in the revised manuscript we provided more detailed discussion on this topic.

The following text was added in the revised manuscript (lines: 172-190).

"The presence of sodium is common in so called halophilic fungi which cannot grow without NaCl[5]. Active discharge, uptake, and efflux processes are likely responsible for the sodium content in the spores and sodium content may vary with different classes and genera of fungi. During active discharge, fungi forcibly eject spores into the atmosphere, together with osmotic fluid containing hexoses, mannitol, phosphate, sodium, and potassium[4,6]. Fungi require K^+ for electrical and osmotic equilibria of the cells and presence of K^+ in several fungal spores is well understood[7]. Previous studies suggested that K^+ can be partially replaced by Na^+ and Na^+ can enhance the growth of fungi[8] and plants[9] under K^+ deficiency conditions. The growth of fungi and uptake of Na^+ varies with their physiological conditions and uptake rates depend on the species and their genes[10]. For example, specific genes (e.g., acu1 and acu2) are responsible for high-affinity Na^+ uptake of Ustilago maydis. When fungi contain excess Na^+, they activate Na^+-efflux ATPase (Adenosinetriphosphatase), which acts as a key enzyme for the biological evolution of fungi[11]. We suggest that uptake and efflux of Na^+ varies with different classes and genera of fungal community. For example, previous studies in Amazonia shows several genera (e.g., Agaricus; Amanita; Aspergillus; Boletus; Cladonia; Mortierella; Puccinia; Lepsita; and Rhizopus) within one class of fungi (Lecanonomycetes)[12,13]. Furthermore, the transpiration of plants[14] and nutrient uptake[15] can also influence the sodium content of spores. Further studies are needed to better comprehend the sodium content in fungal spores by linking chemical composition, molecular biology and diversity of fungal spores."

3. A second finding is that spores which contain salt are more hygroscopic than those which do not. While I'm not aware of any previous results on precisely this topic, it is very well known that salts are deliquescent materials whereas organic macromolecules and other organic compounds found in spores are not. So, this result is very obvious.

Indeed, it is expected that sodium-containing spores are more hygroscopic that those without sodium. Even if the growth factors are similar for sodium chloride particles and sodium containing spores, it is interesting to note that growth factors of NaCl and sodium-containing spores are different at relative humidity between 70 and 96%. Notably, variations in the hygroscopicity are

associated with variations in the chemistry of these spores. We believe this finding is important and worth discussing in the manuscript.

A minor point –

4. According to Figure 3, the Na fraction for fungal spores is estimated to be between 0.1 and 1.0 (i.e. nearly any value in the early hours, and later in the day transitions to an even wider spread in possible fractions. I may be missing something, but I don't find this result to be a significant finding.

We believe Figure 3b provides important contextual information to help readers understand the temporal variability in the simulated fungal spore contribution to sodium, near the measurement site. Considering the high temporal variability of aerosol particle concentrations, including the simulated sea salt and fungal spores in this study (Supplementary Figure 11), it is not surprising that the relative contribution of fungal spores to total sodium mass spans a wide range of values. Figure 3b shows that from midnight to noon, the wet season median contribution of fungal spores to Na ranges from about 0.5 to 0.6. This implies that fungal spores are the dominant contributors of Na on the majority of days at these times. By contrast, from 3 pm to 9 pm, however, the median ranges from 0.15 to 0.3, and the dry season median fungal spore fraction ranges between only about 0.1 and 0.2 (Supplementary Figure 11). In other words, the model helps contextualize the limited number of observations, by identifying the circumstances under which the fungal spores are predicted to contribute the majority to simulated boundary-layer Na at the site: specifically, during the late night and early morning hours of the wet season.

5. In summary, the authors have one potentially interesting surprising field observation in the Amazon. However, additional consideration of how this may arise is needed before I could recommend publication.

We appreciate the comments and suggestions provided by the reviewer. As outlined in the response to the comments 2, in the revised manuscript we expanded the discussion substantially regarding the presence of sodium in fungal spores. In addition, the observation of sodium in fungal spores is on samples from a range of different days.

<u>Reviewer #3 (Remarks to the Author):</u>

China et al. present new multi-instrument observations of the sodium content of fungal spores in the Amazon basin, and use those in combination with backward trajectories and earth system model simulations to suggest that the fungal spores are the main source of sodium containing particles in the wet season.

The major claims of the paper are as follows:

1. Fungal spores contribute significantly to particle-sodium in the Amazon, contributing at least 30% by number of sodium salt containing particles

2. Sodium-containing fungal spores show higher hygroscopic growth than sodium-free spores
3. Fungal spores account for ~62% of sodium mass during the wet season, according to model simulations
4. Therefore, the role of fungal spores in cloud formation and salt cycles in the Amazon deserves further consideration

The claims in the MS are significant and novel. The two closest papers known to me, one from (partly) the same research group, discuss that 1) K-rich bio-aerosols are seeds for SOA formation, but the paper does not mention Na-containing particles (Poehlker et al. 2010) and 2) that when spores rupture at high RH, fungal fragments that contain Na salts are released, but the paper does not quantify Na-content of the particles nor their contribution to the particle Na-budget over Amazonia (China et al. 2016).

The results are of interest to others in the community, since they give 1) new insight into Na-budget of the Amazon from a source that was not considered previously and 2) they add a new line of thought to the debate on sources of cloud-active particles in Amazon, which is a long-standing problem.

The results are of interest to the wider field, because this work adds a new aspect to the understanding of biogeochemical cycles in the Amazon rainforest, and how the forest itself sustains biogeochemical and hydrological recycling. Especially the role of fungal spores therein is one that has not received much attention so far, and this work is likely to start a further debate on this topic.

We appreciate the reviewers' careful reading of our manuscript and providing us positive feedbacks.

The MS is clearly structured and generally convincing, but I think it could profit from some further clarifications:

1) Since I am not familiar with the instruments that are used, I cannot judge the quality of the experimental work. However, I think the MS would benefit from a short explanation of the sampling procedure: are all coarse particles identified that were sampled? How is it determined that a particle is a fungal spore and not another kind of PBA (e.g. bacterium or pollen)? Is this done based on size, morphology or otherwise? A brief description could be added, for instance in L107.

Initially, due to space constraints, we couldn't provide details on the methods used for identifying the fungal spores in the manuscript. Typically fungal spores are 1-6μm, while pollen grains are larger in size (5−150 μm), bacteria are smaller (<1μm) and typically elongated in shape. As we selected only impactor stages 4 and 5 (aerodynamic diameter range: 1.0-3.2 μm) for this study, the fraction of bacteria and pollen is almost negligible. We also refer to our previous publication (China et al., 2016) where we described identification of fungal spores.

To address reviewer's comment, we moved portion of text from the methods section to the main manuscript and provided additional explanations

"The fraction of fungal spores was quantified based on their unique characteristic morphologies (spherical, rod-like or spheroidal in shape), size (1-6 µm), and chemical composition (mostly carbonaceous and containing phosphorous) by electron microscopy imaging and X-ray microanalysis of over 3500 individual particles. Details of the fungal spore identification method are provided elsewhere[16]."

We added the following text in the method section

"The computer-controlled CCSEM/EDX analysis automatically investigates the specified scanning area and detects particles in the specified fields of view. In this way, we can detect ~90% of the particles deposited on the grid. Particles collected on the edges (~5-10%) of the Cu mesh were excluded from the results based on their high Cu background signal. We analyzed ~80% of the particles that were collected onto TEM B-film grids placed on stages 4 and 5 of the impactor."

2. The estimate of the contribution of spores to the Na-budget, depends on two factors which are very uncertain: local emissions of fungal spores and transport of sea salt aerosol into the Amazon basin. I think the fungal spores emissions are constrained reasonably well by the observations of their concentration, which the model seems to underestimate somewhat. However, if global emission estimates of sea salt span 2 orders of magnitude, how reliable is the estimate of sea salt aerosol transported into the Amazon basin, with no direct observations available for validation? Can the authors provide an estimate of the uncertainty in the sea salt contribution to the particle sodium budget? This could provide a bit more context to the number of 62% of total sodium mass that is now assigned to fungal spores.

The reviewer is correct that the model emission estimates of the sea salt are uncertain.

To address this, we compared model estimates of atmospheric sea salt concentration with observed values at few coastal and remote island sites to investigate the fidelity of the model estimates of sodium from sea-salt. We have added a scatterplot comparing observed sodium mass concentration with model-simulated mass sodium mass concentration from sea-salt, together with a global map (Supplementary figure 9) of measurement sites.

We added the comparison of model-simulated and measured sodium mass (Supplementary figure 10) and added the following text.

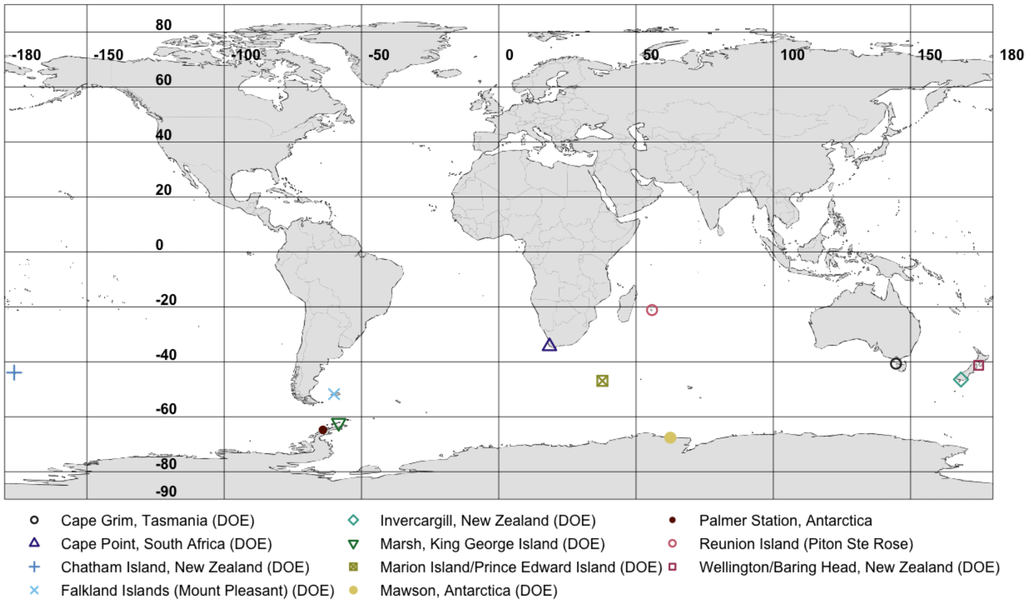

Supplementary Figure 8. Global map of sodium measurement sites, from the AEROCE/SEAREX. *Symbols represent locations where measurements of the atmospheric aerosol sodium mass concentration are available and are compared with model-estimated sodium mass concentration. Note that many sites in this network sampled aerosol only for certain wind directions (sector sampling); sector-sampled sites were excluded from this comparison to remove impacts of sampling bias.*

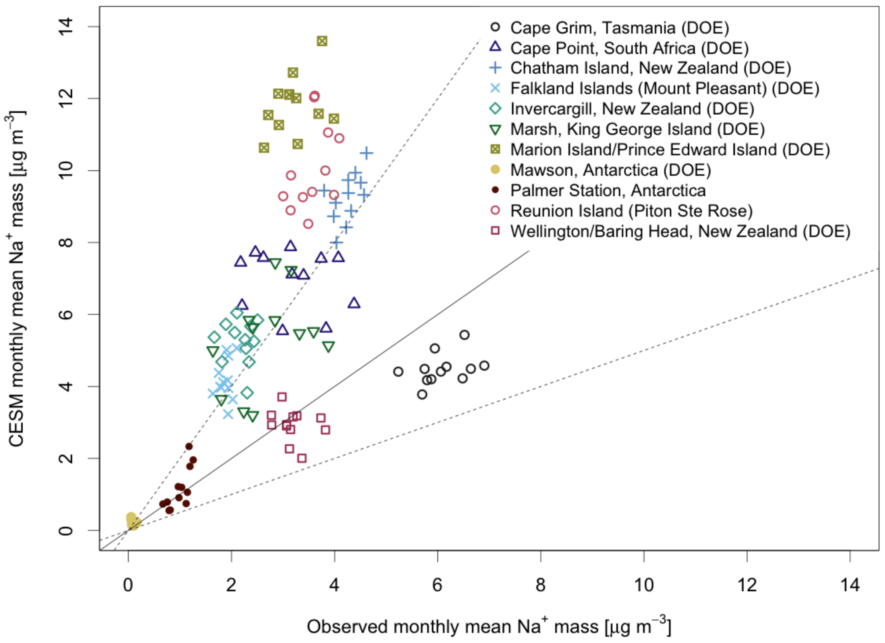

Supplementary Figure 9. Comparison of climatological monthly mean model-simulated and observed sodium mass from sea-salt. *Observations are multi-year climatological monthly*

means of all available years from the AEROCE/SEAREX observational dataset (http://aerocom.met.no/databenchmarks.html)[17-19], collected at sampling sites shown in Supplementary Figure 8. Note that to ensure a representative comparison, this analysis has excluded sites in the measurement network that sampled aerosol only for certain wind directions (sectored sampling). The solid line represents the 1:1 line, and the dashed lines represent an over-or under-estimate of a factor of two (2:1 and 1:2).

"Comparison between model-estimated and measured sodium mass concentrations from sea salt emissions from various geographical locations at the coastal and island sites (Supplementary Figure 9) are illustrated in Supplementary Figure 10. The model estimates of sodium mass concentrations are approximately a factor of two higher than observations at many of the measurement sites; the measurements used in this analysis were all collected at coastal and island locations in the Southern hemisphere, between 20 and 70 S. It is interesting to note that measurements at the remote sites (e.g., Antarctica) are in good agreement with the estimated mass."

"We calculate approximate upper and lower bounds for the fungal spore contribution to the total sodium mass by varying fungal spore mass concentrations by a factor of ten and sea salt mass concentrations by a factor of two. On average, fungal spores are estimated to contribute 69% (31-95%) of sodium during the wet season. However, the level of confidence in fungal spore estimates is arguably higher for the Amazon, since the emissions parameterization was originally developed based primarily on measurements from the Amazon and other tropical rainforests, and simulated fungal spore number concentrations agree with measurements at the Amazonia site within a factor of two. Assuming a factor of two uncertainty bounds for fungal spore concentrations, their estimated contribution to sodium mass would range between 51-84%."

We would also like to note that the sodium mass fraction in sea salt in the original manuscript was underestimated. The sodium mass has been corrected (30% sodium in sea-salt)[20] and all values recalculated. The revised model estimate of fungal spore contribution to the total sodium mass is 69% during wet season.

Minor comments:

1. Title: Since the main finding is that fungal spores contribute significantly to sodium salt particles, why not mention this in the title, for instance: 'Fungal spores as source of sodium salt particles in the Amazon basin'

Based upon the reviewer's suggestion the title is changed to

"Fungal spores as a source of sodium salt particles in the Amazon basin"

2. L29-30: pls add that this is the case for the wet season

We modified the sentence to clarify that the finding is valid for wet season.

"..we show that fungal spores emitted by the forest biosphere contribute to at least 30% (by number) of sodium salt particles observed in the central Amazon basin during the wet season."

3. In L52-53, you state that marine aerosol is considered the main source of coarse aerosol, whereas in L213 you state that fungal spores dominate the coarse mode. It would be good to clarify what is actually meant by dominate: mass, number? And to briefly include the evidence for the statement in L213.

When we state that marine aerosol is considered the main source of coarse aerosol, we meant this was a common assumption, in contrast to our measurements. We meant that fungal spores dominate the coarse mode by number in below-canopy air, and at night, based on our microscopy observations. Fractions of fungal spores are provided in lines 113-116:

"During sampling, the number fraction of fungal spores was higher below the canopy (~60%) than above the canopy (~38%) and higher during night (~52%) than day (~37%)." Figure 1b demonstrate the number fraction of Na-rich particles.

We modified the sentence as follows (lines: 254-255).

"Overall, fungal spores dominated the number fraction of coarse particles in the Amazonia during nighttime and in below-canopy samples, and half of the coarse particles are sodium-rich."

4. L107: how many particles were sampled and how was it ascertained that they are representative for the wet-season coarse aerosol? And how was a particle identified as a fungal spore? Was every collected particle in the sampling periods in Supp. Table 2 classified?

As explained in the reply to the comment 4, we added the information of total number of particles in the main text (line: 111).

"The fraction of fungal spores was quantified .....by electron microscopy imaging and X-ray microanalysis of over 3500 individual particles."

Approximately 80% of the particles that were collected in the sampling period mentioned in the SI Table 2 were classified (see detailed reply in comment 1). We added the number of particles analyzed per sample in the supplementary table. We sampled particles for different durations (~2.5-8 hours) with varying duty cycles to get representative samples both during day- and night-time. We also performed back-trajectory analysis throughout the wet season and overall transport is similar to the transport pattern of airmass that was sampled during our sampling time. In addition, to better understand the representativeness of the analyzed particles with respect to the total particle population, we performed statistical analysis (applying binomial distribution)[21] to estimate the particle sample size of the particle population with given confidence interval (99%) and margin of error. We revised supplementary table 1 to reflect that samples particles are representative of the particle population with 99% confidence interval, and added the margin of error during each of the sampling periods.

As explained in the reply to the comment 1, we have provided additional text (lines: 108-112) in the main manuscript explaining the methods used for identification of fungal spores.

5. L180: since the back trajectories and rain records are based on reanalysis data, I would not refer to them by ‘observations’ here, but rather by ‘simulations’

We changed the sentence to make it clear.

“Hence, these findings support the dominance of locally-emitted spores in the Amazon basin during this study.”

6. L190-191: could you clarify here where the percentages of 70 and 13% for sodium rich-spores and spore sodium mass, respectively, are based on?

We added the following sentences in the revised manuscript to clarify our results (lines: 536-540)

“The percentage of sodium content in fungal spores was estimated using the known mass fraction of spores[22] *and carbon/sodium wt% from X-ray microanalysis. Carbon content in spores ranges from 42-66%, with an average of 51%*[23]*. The average carbon/sodium wt% in fungal spores is 4:1. The mass of the spores is estimated from the size distribution of microscopy analysis and assuming a density of* 1 gm cm^{-3})[22,24].”

Our estimation of 70% represents an upper bound. In the revised manuscript we provide a sensitivity analysis where we use different fractions (e.g., 30% and 50%) of sodium-containing fungal spores estimated from the samples. We add a modeled time series plot of sodium mass contributed by fungal spores assuming 30% and 50% sodium-rich spores in the sample (Supplementary Figure 13). We added few lines (242-245) to present this sensitivity analysis.

“We performed a sensitivity analysis by assuming either 30% or 50% of fungal spores to be sodium-rich. When applying these different assumptions to the model simulations, fungal spores account for 58% (21-90%) or 65% (27-93%) of the total simulated sodium mass during the wet season (Supplementary Fig. 13).”

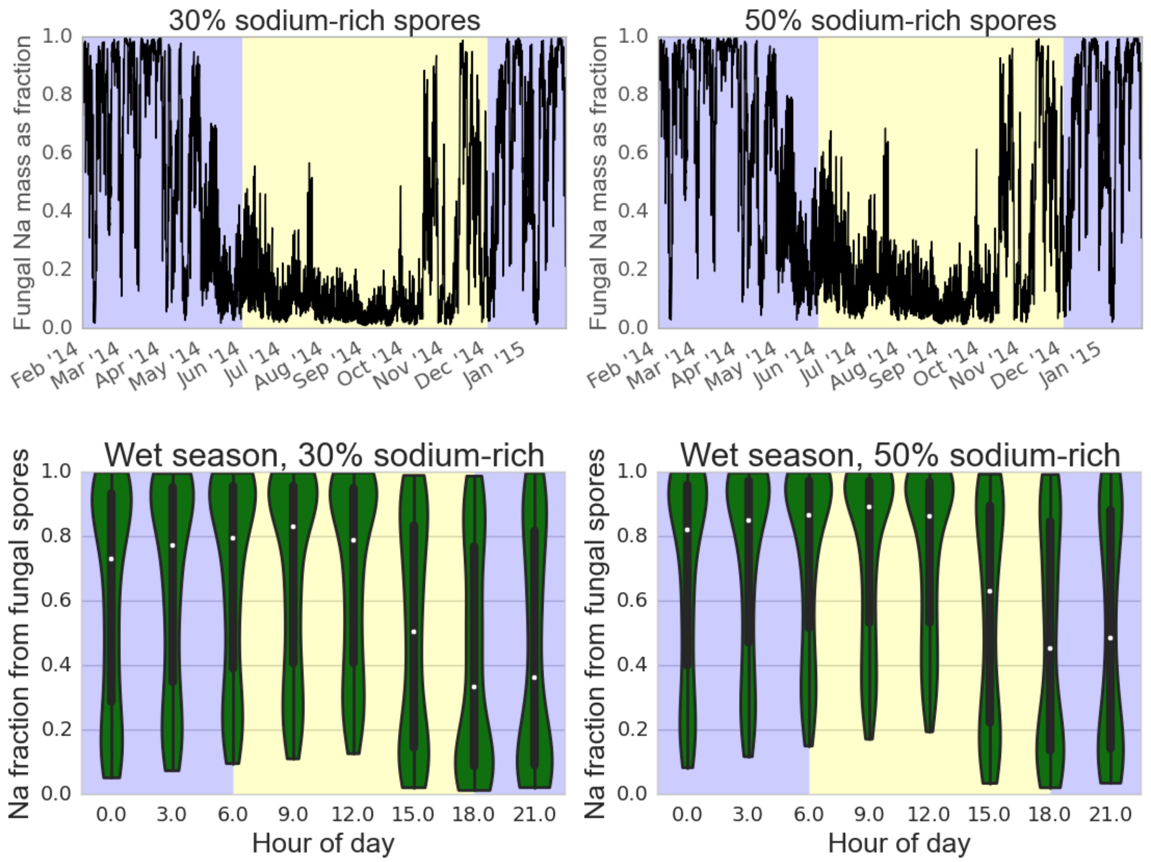

Supplementary Figure 9. Modeled time series of sodium mass contribution from fungal spores assuming different number fractions of sodium-rich spores. *Modeled time series (March 2014 – Feb 2015) of sodium mass percentage contributed by fungal spores assuming a) 30% and b) 50% of spores are sodium-rich.*

7. L208-209: how would nighttime emissions or a shallow nocturnal boundary layer explain that the Na fraction from spores drops only after noon, and not in the early morning at the onset of turbulence? Besides, the violin plots in figure 3 seem to show bimodal distributions for the Na fraction from fungal spores for each time of the day. What is the reason for that?

We suggest that there may be residual particles from the night time emissions that can contribute to the sodium fraction from fungal spores beyond the onset turbulence. It is also possible that high relative humidity throughout the day may be responsible for the higher fraction of fungal spores in the late morning. We added the following senescence in the manuscript.

"This may be the combined result of diurnal cycles in emissions, boundary-layer dynamics, and removal processes (e.g., precipitation)."

The bimodal distribution of the fractional contribution (as seen on the violin plots) is a reflection of the strong variability in simulated sea salt mass concentrations at the site. As a consequence of this variability, most of the time, simulated values of sea salt sodium mass are either much greater or much less than fungal spore sodium mass. Since fractional contributions are presented here, the values cluster in the upper and lower portions of the range 0-1.

8. L217: 'a major fraction to the sodium budget': please add here that this is for the wet season

We clarified that the statement is for the wet season.

"Remarkably, our experimental and modeling results demonstrate that fungal spores emitted from the rainforest biosphere contribute a major fraction to the sodium budget during wet season…"

9. L533-534: which size distribution was assumed for the spores? Within the range of the model coarse mode (1-10 micron), the assumed size can make large differences for the efficiency of the wet and dry removal

We added the following lines to clarify the size distribution used in the model.

"Upon emission both fungal spore mass and coarse mode number are increased, with the mass-to-number ratio for emissions determined by assuming that fungal spores have 4 μm diameter upon emission. The model's coarse mode particle number concentration also includes number contributions from other aerosol species, which are treated as internally mixed within the coarse mode, and the particle diameter is calculated dynamically by the aerosol microphysics routines from the total coarse mode aerosol mass and number, and a fixed geometric width."

10. Supp. Fig. 9: It seems that the same violin plot is show twice: the plot looks very similar to that in Fig. 3 and the title says 'wet season' while the figure is for the dry season. Please include the right plot for the right season.

By mistake we placed the wet season plot. We incorporated the correct dry season plot in the revised manuscript.

11. Technical corrections/suggestions:- L32: pls remove 'we'

Done.

12. L50: similar to concentration of marine air -> similar to concentrations of coarse mode particles in marine air

Corrected.

13. L57: has -> have and is -> are

Corrected.

14. L94: modeling studies -> model simulations

Done.

15. L94: ‘potential contributions of spores’: contributions to what?

Corrected.

16. Fig. 1, caption: representative is misspelled

Corrected.

17. Supp. Table 1: Samples 3A and 3B have an end time prior to the start time

Corrected

18. Supp. Fig. 3: C/Na -> Cl/Na

Corrected.

References

1 Ooki, A., Uematsu, M., Miura, K. & Nakae, S. Sources of sodium in atmospheric fine particles. *Atmospheric Environment* **36**, 4367-4374, doi:https://doi.org/10.1016/S1352-2310(02)00341-2 (2002).

2 Mamane, Y. Estimate of municipal refuse incinerator contribution to philadelphia aerosol-I. Source analysis. *Atmospheric Environment (1967)* **22**, 2411-2418, doi:https://doi.org/10.1016/0004-6981(88)90473-8 (1988).

3 Bourotte, C. *et al.* Association between ionic composition of fine and coarse aerosol soluble fraction and peak expiratory flow of asthmatic patients in São Paulo city (Brazil). *Atmospheric Environment* **41**, 2036-2048, doi:https://doi.org/10.1016/j.atmosenv.2006.11.004 (2007).

4 Pöhlker, C. *et al.* Biogenic potassium salt particles as seeds for secondary organic aerosol in the Amazon. *Science* **337**, 1075-1078 (2012).

5 Plemenitaš, A. *et al.* Adaptation to high salt concentrations in halotolerant/halophilic fungi: a molecular perspective. *Frontiers in Microbiology* **5**, doi:10.3389/fmicb.2014.00199 (2014).

6 Webster, J. *et al.* Mannitol and hexoses are components of Buller's drop. *Mycological Research* **99**, 833-838, doi:http://dx.doi.org/10.1016/S0953-7562(09)80737-5 (1995).

7 Rodríguez-Navarro, A. Potassium transport in fungi and plants. *Biochimica et Biophysica Acta (BBA) - Reviews on Biomembranes* **1469**, 1-30, doi:https://doi.org/10.1016/S0304-4157(99)00013-1 (2000).

8 Camacho, M., Ramos, J. & Rodríguez-Navarro, A. Potassium requirements ofSaccharomyces cerevisiae. *Current Microbiology* **6**, 295-299, doi:10.1007/bf01566880 (1981).

9 Subbarao, G. V., Wheeler, R. M., Stutte, G. W. & Levine, L. H. How far can sodium substitute for potassium in red beet? *Journal of Plant Nutrition* **22**, 1745-1761, doi:10.1080/01904169909365751 (1999).

10 Benito, B., Garciadeblás, B., Schreier, P. & Rodríguez-Navarro, A. Novel P-type ATPases mediate high-affinity potassium or sodium uptake in fungi. *Eukaryotic Cell* **3**, 359-368 (2004).

11 Benito, B., Garciadeblás, B. & Rodríguez-Navarro, A. Potassium- or sodium-efflux ATPase, a key enzyme in the evolution of fungi. *Microbiology* **148**, 933-941, doi:doi:10.1099/00221287-148-4-933 (2002).

12 Womack, A. M. *et al.* Characterization of active and total fungal communities in the atmosphere over the Amazon rainforest. *Biogeosciences* **12**, 6337-6349, doi:10.5194/bg-12-6337-2015 (2015).

13 Fröhlich-Nowoisky, J. *et al.* Biogeography in the air: fungal diversity over land and oceans. *Biogeosciences* **9**, 1125-1136, doi:10.5194/bg-9-1125-2012 (2012).

14 Nemeryuk, G. Migration of salts into the atmosphere during transpiration. *Soviet Plant Physiol* (1970).

15 Lawson, I. T., Jones, T. D., Kelly, T. J., Coronado, E. N. H. & Roucoux, K. H. The geochemistry of Amazonian peats. *Wetlands* **34**, 905-915 (2014).

16 China, S. *et al.* Rupturing of Biological Spores As a Source of Secondary Particles in Amazonia. *Environmental Science & Technology* **50**, 12179-12186, doi:10.1021/acs.est.6b02896 (2016).

17 L., S. D. *et al.* Marine biogenic and anthropogenic contributions to non-sea-salt sulfate in the marine boundary layer over the North Atlantic Ocean. *Journal of Geophysical Research: Atmospheres* **107**, AAC 3-1-AAC 3-21, doi:doi:10.1029/2001JD000970 (2002).

18 Gong, S. *et al.* Modeling sea-salt aerosols in the atmosphere: 2. Atmospheric concentrations and fluxes. *Journal of Geophysical Research: Atmospheres* **102**, 3819-3830 (1997).

19 Li-Jones, X. & Prospero, J. Variations in the size distribution of non-sea-salt sulfate aerosol in the marine boundary layer at Barbados: Impact of African dust. *Journal of Geophysical Research: Atmospheres* **103**, 16073-16084 (1998).

20 Pilson, M. An Introduction to the Chemistry of the Sea Prentice-Hall. *Inc. Upper Saddle River, NJ. 431p* (1998).

21 Levy, P. S. & Lemeshow, S. *Sampling of populations: methods and applications*. (John Wiley & Sons, 2013).

22 Elbert, W., Taylor, P. E., Andreae, M. O. & Pöschl, U. Contribution of fungi to primary biogenic aerosols in the atmosphere: wet and dry discharged spores, carbohydrates, and inorganic ions. *Atmos. Chem. Phys.* **7**, 4569-4588, doi:10.5194/acp-7-4569-2007 (2007).

23 Bauer, H. *et al.* Determination of the carbon content of airborne fungal spores. *Analytical Chemistry* **74**, 91-95 (2002).

24 Huffman, J. A. *et al.* Size distributions and temporal variations of biological aerosol particles in the Amazon rainforest characterized by microscopy and real-time UV-APS fluorescence techniques during AMAZE-08. *Atmos. Chem. Phys.* **12**, 11997-12019, doi:10.5194/acp-12-11997-2012 (2012).

Reviewer #2 (Remarks to the Author):

As I pointed out in my initial review, there are many non-marine sources of sodium salts, which the manuscript failed to discuss. It is frustrating to me that the authors acknowledge this fact in their response and yet refuse to improve the text accordingly. A number of relevant references are missing. In fact, the authors discuss a number of these in their rebuttal but don't include them in the revised text. I find the manuscript seriously lacking in this regard. It is a serious problem in the discussion and introduction. It would seem that the authors are concerned that once the proper references are cited, the novelty of their manuscript will fall beneath the high standards of Nature. Additionally, failure to acknowledge and discuss Na sources is a weakness in the estimates of how much salt in the Amazon comes from marine sources. I appreciate the effort in revising and adding the modeled estimates of sources. However, that section is very simplistic and I question the merit of conclusions drawn from it. Also, the authors state in their response that other Na sources in aerosol are "completely different" because they are small sized aerosols, whereas fungal spores are in the coarse mode. This statement is contradictory to the discussion in the manuscript text that describes the bursting of fungal spores and generation of smaller aerosol as a major reason that fungal spores have climatic relevance. What is known in the literature about the concentration of NaCl in spores? I suspect that a lot more work has been conducted that is not cited here.

The experimental details are sparse. My concern that the highly variable present of salt may simply be a function of operator error (and the occasional contamination of some samples through contain with human skin or contaminated (or re-used) laboratory gloves. I note that another reviewer requested additional details, and the authors chose not to include them, but rather to refer readers to a previous manuscript. I appreciate the limitation of space, but this does not help to eleviate my concern that the experimental results are artifacts rather than new findings.

Reviewer #3 (Remarks to the Author):

The authors have appropriately addressed the comments of me and the other two reviewers. I only have one (very minor) comment on the revised version of the MS: in the main text (lines 236-256), the authors discuss the uncertainty in the 69% contribution of fungal spores to sodium mass, due to assumptions on spore sodium content and sea salt contribution. I would like to see this uncertainty reflected in the number that is given in the abstract, so could you please indicate the uncertainty range there (line 36)?

My recommendation is therefore to publish the paper after this minor revision.

Reply to the Reviewer's comments (NCOMMS-17-33311A)

Reviewer #2 (Remarks to the Author):

As I pointed out in my initial review, there are many non-marine sources of sodium salts, which the manuscript failed to discuss. It is frustrating to me that the authors acknowledge this fact in their response and yet refuse to improve the text accordingly. A number of relevant references are missing. In fact, the authors discuss a number of these in their rebuttal but don't include them in the revised text. I find the manuscript seriously lacking in this regard. It is a serious problem in the discussion and introduction. It would seem that the authors are concerned that once the proper references are cited, the novelty of their manuscript will fall beneath the high standards of Nature. Additionally, failure to acknowledge and discuss Na sources is a weakness in the estimates of how much salt in the Amazon comes from marine sources. I appreciate the effort in revising and adding the modeled estimates of sources. However, that section is very simplistic and I question the merit of conclusions drawn from it. Also, the authors state in their response that other Na sources in aerosol are "completely different" because they are small sized aerosols, whereas fungal spores are in the coarse mode. This statement is contradictory to the discussion in the manuscript text that describes the bursting of fungal spores and generation of smaller aerosol as a major reason that fungal spores have climatic relevance. What is known in the literature about the concentration of NaCl in spores? I suspect that a lot more work has been conducted that is not cited here.

In response to the reviewer's request, we added relevant text in the introduction as noted below (lines: 58-68). Regarding the bursting of fungal spores and generation of smaller particles, we suggest that total contribution of sodium budget from fungal spores can be even lager because we didn't take into account the smaller particles in this study. This study focuses solely on the coarse mode particles and reported results from samples collected on stages 4 and 5 (size range: 1.0-3.2 µm) where the relative abundance of biological particles is high.

"*This study shows locally emitted fungal spores of coarse super-micrometer size contribute considerably to sodium salt particles in the Amazon basin during wet season. While previous studies reported non-marine sources of sodium salts in fine particles, sodium-containing coarse particles have been attributed typically to sea-salt. Specifically, Ooki et al*[1] *observed the existence of anthropogenic sodium in fine mode (<1.1 µm) aerosol particles in urban air. Ooki et al*[1] *suggested potassium as a tracer for anthropogenic sodium in submicron size (0.43µm) particles. Mamane*[2] *found presence of sodium and potassium in <1 µm particles from waste incinerators emissions. Another study conducted in the metropolitan area of São Paulo found K/Na ratio of 1.4 and 0.9 for coarse (>2.5 µm) and fine (<2.5µm) particles respectively and they attributed this particles to waste incineration and vehicles*[3]."

We added the following text in the discussion (lines: 268-273)

"Previous studies in different atmospheric environment show the presence of sodium in fine mode and suggest potassium as a tracer for anthropogenic sodium[1-5]*. Observations of our study are fundamentally different. First, our study focuses on the coarse mode particles. Second, the potassium observed in our study mostly originates from biological particles*[6] *confirmed by their*

unique and distinguishable morphology. Those particles were especially abundant during the wet season when biomass burning is not a dominant source of particles in the basin."

The experimental details are sparse. My concern that the highly variable present of salt may simply be a function of operator error (and the occasional contamination of some samples through contain with human skin or contaminated (or re-used) laboratory gloves. I note that another reviewer requested additional details, and the authors chose not to include them, but rather to refer readers to a previous manuscript. I appreciate the limitation of space, but this does not help to eleviate my concern that the experimental results are artifacts rather than new findings.

As we discussed in our revised manuscript, we suggest that variable present of sodium content may depend on different classes and genera of fungi. Sample collection and experiments were conducted with cautions. In our previous revised version as requested by the reviewer 3, we provided details of the method (please see the notes of the reviewer 3 who found our arguments convincing). In addition to that, we provide following discussions, which suggest that presence of sodium salt in our sample indeed from spores rather than contamination.

i) We routinely investigate quality of our blank substrates from different batches (total 5 in this case). SEM images at different field of view show that blank sample are with negligible contamination (see Supplementary Figure 14). We found 1 particle per 0.0005 cm^2 area, where as field collected particles, on average, consist of ~500 individual particles on stages 4 and 5. Particle loading is higher for smaller size particles (~2100 particles per 0.0005 cm^2 area on stages 6 and 7, see Supplementary Figure 15a, b).

ii) As we mentioned earlier, this study reports samples from stages 4 and 5 (size range: 1.0-3.2 µm). However, we also investigate particles from lower stages 6 and 7, size range: 0.32-1.0 µm) for a separate study. Fractions of Na-containing particles in stages 6 and 7 are significantly lower (Na-containing particle <15%) than particles on stages 4 and 5 (~50%). Please see Supplementary Figure 15c. This result suggest that high fraction of sodium-salt particles are indeed from spores that were deposited on stages 4 and 5.

iii) Na-content in spores were investigated using different techniques (electron microscopy and Nano- secondary ion mass spectrometry) with different operator, sample preparation and sample handling procedures. Irrespective of that, both methods show presence of sodium in spores.

To elaborate more on this note, we added two new Supplementary Figures (14 and 15) and the following text (lines: 555-564) in the method section of the revised manuscript.

"Samples were handled with caution. For examples, tweezers were cleaned with solvents prior to handling the samples; new gloves were used for handling of samples after each experiments. Examination of blank substrate showed negligible contamination on the sample substrate (Supplementary Figure 14). Investigation of particles from stages 6 and 7 (size range: 0.32-1.0 µm) showed Na-containing particles in stages 6 and 7 are significantly lower (Na-containing

particle <15%) than particles on stages 4 and (Supplementary Figure 15). This result suggest that high fraction of sodium-salt particles are indeed from spores that were deposited on stages 4 and 5. Furthermore, sodium content in spores was observed irrespective of different sample preparation and techniques (e.g., SEM/EDX, STXM and NanoSIMS)"

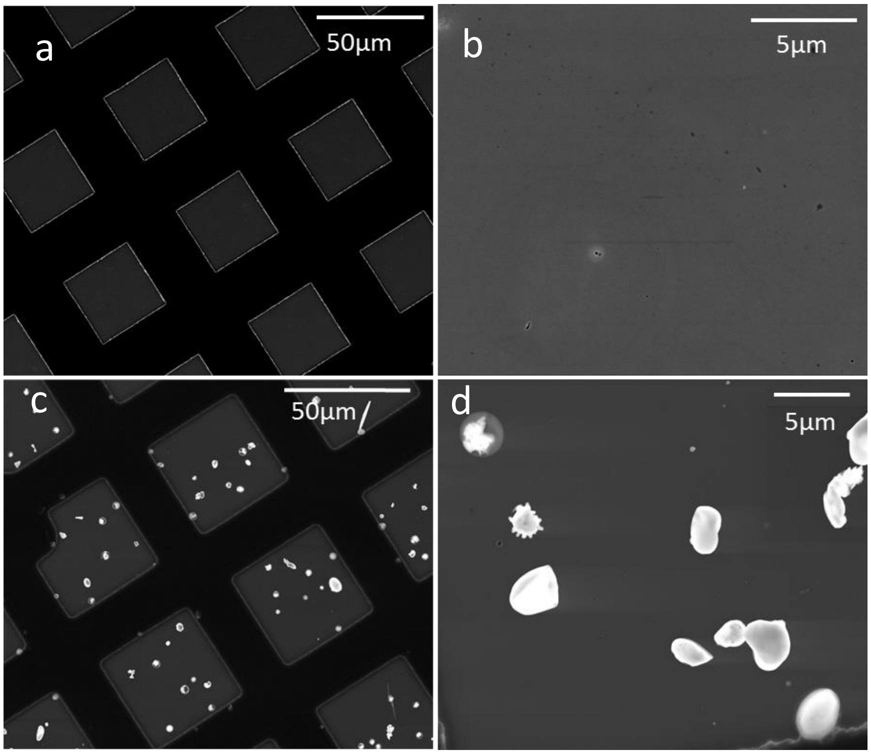

Supplementary Figure 14. Investigation of particle contamination. *SEM images show a) low magnification and b) high magnification image of blank sample; c) low magnification and d) high magnification image of collected sample.*

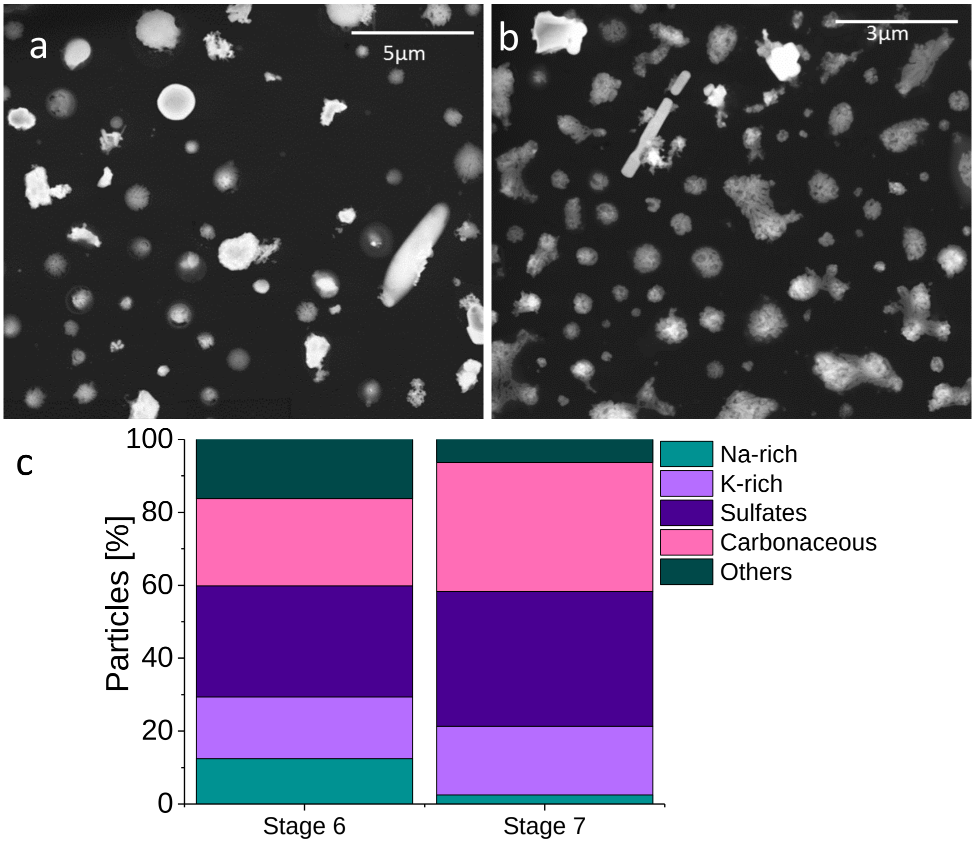

Supplementary Figure 15. Fraction of Na-rich particles in smaller size (stages 6 and 7) particles. *Representative SEM images of particles collected on a) stage 6 (0.56-1.0 µm), b) stage 7 (0.32-0.56 µm), c) number fraction of different particle classes for particles collected on stages 6 and 7. Fraction of Na-rich particles is <15%.*

Reviewer #3 (Remarks to the Author):

The authors have appropriately addressed the comments of me and the other two reviewers. I only have one (very minor) comment on the revised version of the MS: in the main text (lines 236-256), the authors discuss the uncertainty in the 69% contribution of fungal spores to sodium mass, due to assumptions on spore sodium content and sea salt contribution. I would like to see this uncertainty reflected in the number that is given in the abstract, so could you please indicate the uncertainty range there (line 36)?

My recommendation is therefore to publish the paper after this minor revision.

As suggested, we provided the uncertainty range in the abstract as well.

"Modeling results suggest that fungal spores account for ~69% (31-95%) of the total sodium mass during the wet season and that their fractional contribution increases during nighttime."

References

1 Ooki, A., Uematsu, M., Miura, K. & Nakae, S. Sources of sodium in atmospheric fine particles. *Atmospheric Environment* **36**, 4367-4374, doi:https://doi.org/10.1016/S1352-2310(02)00341-2 (2002).

2 Mamane, Y. Estimate of municipal refuse incinerator contribution to philadelphia aerosol-I. Source analysis. *Atmospheric Environment (1967)* **22**, 2411-2418, doi:https://doi.org/10.1016/0004-6981(88)90473-8 (1988).

3 Bourotte, C. *et al.* Association between ionic composition of fine and coarse aerosol soluble fraction and peak expiratory flow of asthmatic patients in São Paulo city (Brazil). *Atmospheric Environment* **41**, 2036-2048, doi:https://doi.org/10.1016/j.atmosenv.2006.11.004 (2007).

4 Kitto, M. E. Trace-Element Patterns in Fuel Oils and Gasolines for Use in Source Apportionment. *Air & Waste* **43**, 1381-1388, doi:10.1080/1073161X.1993.10467213 (1993).

5 Mizohata, A. & Mamuro, T. Chemical element balances and identification of air pollution sources in Sakai, Osaka (I). *Journal of Japan Society of Air Pollution* **15**, 198-206 (1980).

6 Pöhlker, C. *et al.* Biogenic potassium salt particles as seeds for secondary organic aerosol in the Amazon. *Science* **337**, 1075-1078 (2012).
